# Optimizing Hospital Discharge Planning: Empirical Insights and Requirements of AI-Based Technologies From an Explorative Mixed Methods Field Study

**DOI:** 10.2196/81824

**Published:** 2026-03-24

**Authors:** Johanna Sadel, Natalie Victoria Grant, Heinrich Burkhardt, Christophe Kunze

**Affiliations:** 1 Care & Technology Lab, Furtwangen University, Robert-Gerwig-Platz 1, Furtwangen, 78120, Germany, 49 7723 920 2960; 2 Medical Faculty Mannheim, Heidelberg University, Heidelberg, Germany

**Keywords:** care transitions, artificial intelligence, human-centered design, health technology design, discharge planning, mixed methods

## Abstract

**Background:**

Discharge planning (DP) is crucial for care continuity after a hospital stay but remains complex due to organizational constraints, interprofessional coordination, and administrative demands. Despite ongoing digitalization efforts, many health technologies overlook the sociotechnical nature of discharge processes, limiting acceptance and integration into clinical workflows.

**Objective:**

This study aimed to examine real-world DP practices in 2 German university hospitals, identify user-centered needs, and derive design implications for responsibly integrating artificial intelligence (AI)–based systems into DP.

**Methods:**

A mixed methods field study was conducted combining qualitative and quantitative approaches. In the qualitative phase, DP employees participated in workshops (n=33). Additionally, expert interviews were conducted with 2 physicians and 3 nurses (n=5). Activities explored understanding of AI, challenges in DP workflows, and best-case process scenarios; existing processes were collaboratively modeled to identify potential intervention points for technological support. Transcripts were analyzed inductively following Mayring’s qualitative content analysis. Quantitative data were collected through a standardized questionnaire (n=23), focusing on workload distribution, process inefficiencies, and openness to using AI in the DP context. Descriptive statistics were used to identify high-burden segments. Findings were integrated through methodological triangulation.

**Results:**

Persistent challenges emerged in interdisciplinary communication, documentation practices, and information continuity. Participants expressed uncertainty about the value of AI in DP, emphasizing the need for transparency, explainability, and role alignment. Questionnaire data confirmed bottlenecks in information transfer and high administrative workload. Design requirements for future systems include process transparency, support for coordination tasks, and adaptability to clinical roles.

**Conclusions:**

DP is a sociotechnical process in which human expertise and organizational context must guide system design. Participatory, context-aware design approaches are essential for integrating AI into clinical practice. Aligning technology with everyday workflows can increase acceptance and yield more effective digital interventions.

## Introduction

### Overview

Western health care systems are facing increasing pressure due to demographic change, rising number of patients with multimorbidity, workforce shortages, and growing cost burdens [1,2]. One area particularly affected by these developments is the transition from inpatient care to aftercare. In this context, discharge planning (DP) plays a central role. DP refers to the structured assessment of posthospital care needs and the coordination of appropriate medical, nursing, rehabilitative, and psychosocial services to ensure continuity and safety across care sectors [3-5]. As treatment pathways become more complex and patient needs more individualized, effective DP requires close interprofessional collaboration and timely access to accurate information [6].

When DP is insufficient or delayed, the consequences can be significant. For patients (especially older adults and those with cognitive or functional limitations), this may result in fragmented care, deterioration of health status, or avoidable rehospitalizations. At the system level, poor DP contributes to increased readmission rates, higher caregiver burden, and additional strain on outpatient and community-based services [7,8]. A structured and well-coordinated approach to DP is therefore essential for ensuring integrated and patient-centered care.

In response to these challenges, Germany has introduced a comprehensive legal framework mandating DP as a core component of inpatient care. The foundation for this was established in § 39(1a) of the Fifth Book of the German Social Code (SGB V), which governs statutory health insurance and health care provision in Germany, and strengthened through subsequent reforms, including the Act to Strengthen Care in Statutory Health Insurance, German health care reform law enacted in 2015 to improve care continuity. A national framework agreement further operationalizes these requirements by defining procedural standards, early needs assessment, and patient involvement across the hospitalization process. Hospitals are thus obligated to ensure timely coordination of follow-up services and continuity of care, with compliance regularly reviewed through quality assurance mechanisms.

Despite these regulatory advances, DP in practice remains highly variable and shaped by organizational constraints, staffing limitations, and heterogeneous documentation and communication practices. In many cases, workflows rely heavily on professional experience, informal coordination, and manual information transfer. Against this backdrop, digital tools and artificial intelligence (AI)–based systems are increasingly discussed as potential means to support DP by improving documentation efficiency, data availability, communication, and planning accuracy [9-11]. However, although digital technologies are gaining attention in DP, research on AI-based tools in this context remains limited, particularly regarding their meaningful integration into everyday clinical practice. This gap has led to targeted funding initiatives from the Federal Ministry of Health (Federal Ministry in Germany responsible for health care policy and public health regulation) and the Federal Ministry of Research, Technology and Space (Federal Ministry in Germany responsible for supporting research, technology, and aerospace initiatives) promoting research on AI in clinical practice [12].

User-centered and participatory approaches are considered essential for the successful development and adoption of care technologies [13]. Yet, only a minority of AI projects meaningfully involve clinical end users in early design phases [14], in part because effective participation requires both technological transparency and context-sensitive design processes [15]. This study addresses these gaps by examining DP as experienced by health care professionals in 2 German university hospitals. We analyze how discharge processes unfold in practice, which organizational and communicative challenges shape them, and which forms of digital or AI-based support could meaningfully address these challenges. Based on a mixed methods approach, we derive user-centered design requirements for the responsible and context-aware integration of AI into DP.

### Background and Related Work

Research on DP has used a wide range of methodological approaches to understand its clinical and organizational complexity. Quantitative studies have primarily evaluated the effectiveness of DP interventions in terms of measurable outcomes such as readmission rates, length of stay, and mortality [16]. Meta-analyses indicate that structured communication interventions at discharge can reduce 30-day readmissions [17], and systematic DP is associated with reductions in length of stay and readmission rates for certain patient groups [16].

Complementing these findings, qualitative studies have highlighted the social and organizational challenges of implementing DP in daily practice. Reported barriers include limited standardization, inconsistent communication across professional groups, and time pressure [18]. Across multidisciplinary teams, unclear roles and fragmented information flows are frequently cited as central constraints [19].

In parallel, technological approaches such as machine learning and AI are increasingly being explored to support DP. Machine learning models have been developed to predict discharge destinations [20,21], and neural network–based tools are being tested to support discharge decision-making [22,23]. Large language models are also being evaluated for automating or simplifying clinical documentation [24,25]. While these tools show potential for improving patient flow and reducing administrative burden, robust evidence for their clinical efficacy remains limited. Only a small number of systems currently achieve reliable, automated discharge prediction at scale [22].

A notable gap in the literature is that most existing work remains primarily quantitative and outcome-oriented, focusing on predictive accuracy rather than everyday implementation contexts. Studies incorporating the perspectives of clinical staff are comparatively rare, despite the fact that DP operates across social, organizational, and bureaucratic interfaces. Mixed methods research that integrates process understanding with performance outcomes therefore remains limited [26]. Furthermore, while patient experience has received increasing attention, less is known about how digital and AI-supported tools can be designed to meet the needs of the professionals who carry out DP [16].

Given that DP is a cross-sectoral, relational, and dynamically negotiated process, user-centered research is essential for developing digital systems that support, rather than disrupt, clinical workflows. This study addresses this gap by examining DP as practiced in daily work and deriving design implications for AI-supported tools directly from the perspectives of DP professionals.

## Methods

### Study Design

This study used a mixed methods design with participatory elements to identify user-centered requirements for AI-supported tools in DP. The mixed methods approach was chosen to capture both the sociotechnical and procedural dimensions of DP, recognizing that digitalization efforts often foreground technical implementation while the organizational and social contexts of work remain underexamined.

The study design followed the GRAMMS (Good Reporting of a Mixed Methods Study) framework for mixed methods research [27] and drew on selected MMR-RHS (Mixed Methods Reporting in Rehabilitation & Health Sciences) criteria [28]. The qualitative and quantitative strands addressed complementary aspects of the same research question. The qualitative strand (priority strand) focused on the organizational realities of DP, including communication structures, professional roles, and perceived challenges in daily practice. The quantitative strand extended these insights by examining (1) the distribution of administrative, coordinative, and documentation-related tasks; (2) satisfaction with procedural aspects of DP; and (3) expectations and concerns regarding the integration of AI-based systems into clinical workflows.

Data collection proceeded in a partly sequential and partly concurrent manner. Participatory workshops and interviews were conducted first to surface practice-relevant themes, followed by a standardized questionnaire administered immediately afterward to confirm and broaden the emerging patterns. This sequencing allowed the quantitative data to extend the qualitative insights while ensuring that both strands addressed shared conceptual domains. The integration of both strands occurred during the interpretation stage to develop metainferences about DP practices and potential technological support. Details of the analytic procedures and integration approach are described in the “Data Analysis” section.

In accordance with current reporting guidelines, we disclose that generative AI was used to assist in creating a flowchart illustrating the DP process. All prompts and AI outputs related to the flowchart creation are documented in Multimedia Appendix 1 for transparency.

### Setting

Both sites are maximum care providers (*Maximalversorger*). This designation identifies institutions that can provide the highest level of care available in Germany. The classification is based on factors including bed capacity and scope of services offered.

- *Hospital A* (*2023*): A total of 1352 beds; >30 clinics and institutes; and 44,176 inpatient, 5489 day-case, and 318,097 outpatient cases. DP is organized under the nursing directorate with 2 units (social services and care transition).
- *Hospital B* (*2023*): A total of 1306 beds; 40 clinics and 34 institutes; and 54,757 inpatient, 2040 day-case, and 459,752 outpatient cases. DP is organized under the medical directorate with 2 professional groups (social service and case management) working in clinic-assigned teams.

Both hospitals operate heterogeneous, multivendor IT environments spanning administration, imaging, documentation, patient flow, and DP. Each site uses specialized software for case management and for identifying or arranging postacute providers, reflecting the fragmented and interoperability-constrained IT landscape typical of German hospitals (see Textboxes1  2 for DP consulting areas) [29].

Textbox 1. **Consulting areas of the departments in discharge planning of hospital A.**

**Department social service**

- Medical and other rehabilitation measures.
- Social law matters.
- Inpatient care.
- Financial aid options.
- Counseling centers—therapeutic services.
- Discussion opportunities in the areas of coping with illness, future prospects, partnership and family, and social and professional environment.

**Department care transition**

- Advice on long-term care insurance.
- Advice on medical aids and support in their provision.
- Advice on the use of an outpatient care provider.
- Handover of necessary activities to an outpatient care provider.
- Provision of specialized outpatient palliative care and hospice registration.
- Neighborhood assistance.
- Meals and Wheels.
- Emergency call system.
- Home Care Tuesday.
- Referral to other counseling centers or care centers.

Textbox 2. **Consulting areas of the departments in discharge planning of hospital B.**

**Department social service**

- Support in initiating rehabilitation measures.
- Support in arranging assistance in everyday life.
- Social law matters.
- Financial aid options.

**Department case management**

- Coordination in interdisciplinary service provision.
- Application for a care level.
- Advice and mediation of rehabilitation measures.
- Organization of nursing services to care for patients at home.

### Participants and Recruitment

Participants were professionals involved in DP at 2 German university hospitals. A total of 33 nonmanagerial DP staff members took part in the workshops, and 23 of them completed a standardized questionnaire administered immediately afterward (69% response rate). Recruitment for the workshops and questionnaire was coordinated by the on-site researcher (NVG). Managers were contacted via email, introduced to the project through a brief presentation, and asked to indicate interest. Workshops were scheduled during working hours based on staff availability.

To incorporate additional clinical perspectives, semistructured interviews were conducted at hospital A with physicians and nursing staff using convenience sampling within the on-site researcher’s professional network. Five physicians and 5 nurses were invited, of whom 2 physicians and 3 nurses participated (response rates: 40% and 60%). No interviews were conducted at hospital B due to the absence of an on-site researcher during the data collection period.

Demographic details (eg, age, gender, and specialty) were not collected to maintain confidentiality, given the small size of DP teams (approximately 15‐20 staff per site), in accordance with ethics committee and works council requirements. Table 1 provides an overview of data collection methods, duration, and participant groups.

**Table 1.** Overview of data collection methods, duration, and number of participants according to university hospital.

Method	Duration	Participants, n
Workshop (hospital A)	2-hour sessions, 4 hours in total	14
Workshop (hospital B)	4 hours	19
		Total = 33
Questionnaire (hospital A)	15 minutes	8
Questionnaire (hospital B)	N/A^a^	15
		Total = 23
Interviews (hospital A)	Range 30-55 minutes; average 44 minutes	2 physicians; 3 nurses
		Total = 5

a N/A: not applicable.

### Data Collection

A multimethods design was used, combining qualitative (workshops and semistructured interviews) and quantitative (standardized questionnaires) data collection.

#### Qualitative Data Collection

Qualitative data were gathered through participatory workshops with DP staff to identify challenges, needs, and expectations in everyday practice. Each workshop followed a structured, 3-phase format guided by open-ended questions to promote discussion and reflection. The sessions aimed to (1) explore participants’ understanding of AI in health care, (2) assess perceived opportunities and limitations of AI in DP, and (3) collaboratively map both the current and an ideal (best-case) discharge process.

At hospital A, eight 2-hour sessions were conducted over 4 weeks (July-August 2023), with each participant attending 2 consecutive sessions. At hospital B, two 4-hour sessions were held on consecutive days in July 2024, adjusted to staff availability. Each workshop included (1) an introduction and warm-up task exploring participants’ experience with AI, (2) a collaborative stakeholder and process mapping exercise, and (3) a discussion on potential technological entry points and influencing factors. Discussions were moderated by researchers and documented using flip charts, Post-it notes, and photographs.

Semistructured interviews with physicians and nurses complemented the workshops to capture additional clinical perspectives. Interviews lasted 30‐55 minutes and followed a predefined guide. Sample questionnaires are provided in Multimedia Appendices 2-4. All workshops and interviews were audio-recorded using a ZOOM H5 device and transcribed verbatim for analysis.

#### Quantitative Data Collection

A standardized questionnaire was administered at both hospitals, covering domains related to workload distribution, satisfaction with procedural aspects of DP, and attitudes toward AI-based support. Items were rated on a 5-point Likert scale (1=strongly disagree to 5=strongly agree) and included open-ended questions. The questionnaire was identical across both sites and distributed in paper form immediately after the workshops. In line with the exploratory and iterative design of this study, the initial questionnaires (developed in 2023) were intentionally broad to capture a wide range of aspects related to DP. At that early stage, the aim was to map the field rather than to test predefined hypotheses. A sample questionnaire is provided in Multimedia Appendix 5.

### Data Analysis

Qualitative data from the workshops and semistructured interviews were fully transcribed and analyzed using the software MAXQDA (VERBI). Following Mayring’s [30] approach to qualitative content analysis, we applied inductive category development to derive themes grounded in the data. Coding proceeded iteratively, with categories refined through comparison across both hospital sites and professional roles. The coding system is included in Multimedia Appendix 6. A tabular overview of coded segments can be viewed in Multimedia Appendix 7. An overview of statements sorted by workshops is provided in Multimedia Appendix 8. The analysis focused on identifying recurrent patterns related to communication structures, role distribution, workload organization, and perceived opportunities and limitations for AI-supported tools. All qualitative data were collected in German. For publication, excerpts were translated into English by bilingual members of the research team, prioritizing conceptual accuracy over literal phrasing.

Questionnaire responses were analyzed descriptively (proportions and percentages) to identify salient issues. For this paper, only those items that conceptually aligned with themes emerging from the qualitative strand were retained for the integrated content analysis (workload distribution and satisfaction with procedural aspects of DP). Items that were unrelated to these integrated findings are not reported here, as they fall outside the analytical scope of this publication but remain part of the broader dataset.

The qualitative and quantitative strands were merged during the interpretation phase through methodological triangulation [31]. This integration enabled comparison of qualitative themes with quantitative indicators to assess convergence and divergence, supporting the development of context-sensitive design requirements for AI-supported systems in DP.

### Ethical Considerations

This study was reviewed and approved by the Ethics Commission II (Heidelberg University, Medical Faculty of Mannheim) under reference number 2023‐523. A subsequent review was obtained from the Ethics Commission for the second participating clinic (University Clinic Bonn) under reference number 246/23-EP. In addition, approval was granted by the local Works Council of both participating institutions. All procedures were conducted in accordance with the ethical standards of the participating universities, the Declaration of Helsinki, and relevant national legislation. Written informed consent was obtained from all participants prior to their inclusion in the study. Participants did not receive any financial or material compensation for their participation. No patient data were collected in this study. Data collection was limited to health care professionals participating in workshops, interviews, and questionnaires. No personally identifying clinical or patient-related information was recorded. All study data were handled in accordance with applicable data protection regulations. Written informed consent forms were stored separately from the research data in locked cabinets at the respective study sites. Audio recordings and transcripts were anonymized prior to analysis, and all digital data were stored in anonymized form only. Access to the data was restricted to members of the research team.

## Results

### Quantitative Data

Questionnaire items that conceptually corresponded to the qualitative themes were analyzed and are reported here. Data are reported descriptively without inferential testing owing to the small sample size and the exploratory character of the study. Items outside this analytical focus are not presented, as they did not contribute to the integrated interpretation. Tables2  3 provide an overview of the use of working time spent on documentation and coordination as well as satisfaction with procedural aspects of DP, respectively.

**Table 2.** Reported proportion of working time spent on documentation and coordination activities (n=22 for item 5, n=23 for item 6).

Item ID	Question (English translation)	0%‐25%	26%‐50%	51%‐75%	76%‐100%
5	What percentage of your working time do you spend on documenting information in order to determine the need for aftercare?	6	8	4	4
6	What percentage of your working time do you spend consulting with other staff members to coordinate patient treatment and aftercare?	5	13	4	1

**Table 3.** Response distribution for selected items on time, communication, and coordination in discharge planning (N=8).

Item ID	Question (English translation)	Strongly agree, %	Agree, %	Neutral, %	Disagree, %	Strongly disagree, %
1	Is the time you have for discharge planning often too short?	25	75	—^a^	—	—
2	Do you think that the discharge planning usually runs smoothly?	—	—	—	75	25
9	Does communication with other staff members (physicians and nursing staff) work well during discharge planning?	—	25	—	75%	—
10	Do you receive timely feedback from other staff members (physicians and nursing staff) when something changes in the transition planning?	—	12.5	—	75	12.5
12	Do you have enough time to plan the transition to aftercare?	—	12.5	—	87.5	—
13	Do you receive discharge notifications too late?	25	50	—	12.5	12.5

a Not available.

Response options indicate the estimated proportion of working time spent on the respective activities (0%‐25%, 26%‐50%, 51%‐75%, and 76%‐100%). Data are reported as counts (n) to avoid confusion with percentage-based response categories. Responses are from hospital A and hospital B. Questions are originally in German. Most respondents reported spending between 26% and 50% of their working time on documentation related to determining aftercare needs (8/22, 36%) and a similar share on coordination with other staff (13/23, 56%). Only a few indicated spending more than 75% of their time on either activity. The percentages represent the distribution of responses on a 5-point Likert scale (1=strongly disagree, 5=strongly agree). Responses are from hospital A only. Questions are originally in German. Descriptive frequencies showed agreement that the time available for DP was insufficient (100% agreement; item 1) and disagreement that DP runs smoothly (100% disagreement; item 2). Similar patterns of disagreement were observed for items on communication and feedback (items 9 and 10).

### Integrated Content Analysis

Three thematic areas emerged from the analysis: (1) aspects describing the structure of the DP process (process description), (2) factors influencing its success (success factors), and (3) derived implications for the design of AI-supported tools in DP (core requirements). These 3 areas will be outlined in detail in the following section. Textbox 3 provides an overview of the main findings.

Textbox 3. **Overview of main themes derived from the integrated content analysis.**

**Process description**

- Initiating discharge planning (DP).
- Needs assessment and initial contact.
- Counseling and psychosocial intervention.
- Information from patients and clinical staff.
- Discharge planning.
- Implementation of DP measures.
- Final coordination and discharge preparation.

**Success factors**

- Staffing and personnel availability.
- Completeness and timeliness of information.
- Departmental and professional practice.
- Time constraints and deadlines.
- Documentation and consent processes.
- Changing individual care needs.
- Availability of aftercare services.

**Core requirements**

- Communication and notification.
- DP and aftercare planning.
- Data management and documentation.
- Needs assessment and responsibility clarification.

### Process Description

Participants described the DP process as a reactive and highly individualized response to the situational demands of daily hospital operations. According to their accounts, the implementation of DP often depends heavily on the expertise, experience, and judgment of the individual staff members involved.

*There is a process description for how discharge planning is supposed to work in theory, but in practice it deviates quite a lot. There is no single standard that everyone follows, and nothing like a ‘light’ version either. Much of the procedure is passed on orally. It lives in our heads and you have to learn it over time. I have a sort of process map in my mind: when a case comes in, I know at which points certain decisions need to be made and what comes next. But that depends very much on the information we receive, and often, that information is incomplete. So, we frequently have to fill in gaps or cover tasks from other departments before we can even begin our part of the process*.

[Participant, Workshop 1, Hospital A]

Although a standard for DP is provided by the German Network for Quality Development in Nursing (DNQP), it primarily defines general phases and criteria rather than prescribing a concrete procedural framework. DNQP is a German network that develops expert standards to promote quality improvement in nursing practice [32]. While widely recognized and used as a reference in quality assurance, the DNQP standard remains advisory rather than legally binding, offering guidance rather than strict regulation. Moreover, neither the legal frameworks defined in the introduction nor the internal organizational guidelines provide a detailed, binding sequence of steps. Instead, certain key objectives such as timely DP, interdisciplinary coordination, and continuity of care must be achieved. These pillars aim to ensure legal compliance while allowing for flexible adaptation to the individual needs of patients.

In light of this flexibility, and in agreement with participants, the decision was made to present a best-case scenario for DP as there is no universally standardized process across departments. This model is grounded in shared structural elements and clearly defined steps, forming a general process framework that supports effective and patient-centered DP. Beyond its descriptive function, the model serves as a design-oriented artifact: it captures practitioners’ normative expectations of how DP should function under optimal conditions. As such, it provides a valuable reference point for identifying friction points in current practices and for mapping potential entry points for AI-supported interventions. This best-case framing allows for a participatory, practice-based foundation to guide system design in line with real-world workflows and professional logics. The following sections outline the key components of this idealized model. A best-case process model can be found in Multimedia Appendix 9.

#### Initiating DP

The DP process typically begins with a clinical referral issued by medical staff. This involves formally requesting the services of the hospital’s DP team to coordinate necessary postdischarge care. A referral is made when a medical, therapeutic, or nursing need for aftercare has been identified. According to the DNQP standard, a criteria-based initial assessment must occur within 24 hours of assuming responsibility for the patient’s nursing care. This is usually performed by medical or nursing staff and serves as the formal prerequisite for initiating DP. Referrals may be issued by nursing staff, physicians, or directly by patients or their legal representatives. In practice, however, this process often deviates from the standard due to time constraints, limited systematic assessments during admission, and insufficient interdisciplinary communication.

*At this point, we’re simply relieved when we receive a notification from the nursing team at all. We no longer focus on what is written in the referral. We just hope it comes in on time. Too often we find out only the day before discharge, when we’re told, “Oh, we forgot. The patient is leaving tomorrow and still needs several things arranged*.”

[Participant, Workshop 1, Hospital A]

Once a need for aftercare is identified and a referral is made, patients or their legal representatives are informed and receive counseling. Written consent is required to proceed, and no further steps may be taken without it. Even in cases of objective need, not all patients choose to participate. Once consent is provided, a detailed needs assessment follows.

#### Needs Assessment and Initial Contact

At this stage, the social services and nursing transition teams assess the patient’s individual aftercare needs. This involves reviewing the medical record and conducting a personal conversation with the patient or relatives to gather contextual information on the home environment, care situation, available resources, and support systems. To plan appropriate aftercare, DP staff require comprehensive insights into the patient’s broader living context. Textbox 4 outlines key categories of discharge-relevant information.

Textbox 4. **Key types of discharge-relevant information as reported by participants during workshops and interviews.**

- Contact details for patients and their relatives.
- Information on legal capacity and cognitive impairments.
- Existing levels of care and current care needs.
- Language or communication barriers.
- Living and care arrangements prior to hospital admission.

Among these, contact information is particularly crucial for enabling communication. Early clarification of legal competence and cognitive capacity is also important. Ideally, communication challenges such as hearing or speech impairments are identified early. If a care dependency level was previously established, it should be documented at admission to streamline planning. The DP team also consults with physicians and nursing staff to estimate the discharge date and assess the need for follow-up services. A commonly used assessment tool is the Barthel Index, which evaluates a patient’s ability to perform basic activities of daily living and supports care planning. The Barthel Index is often applied in rehabilitation and geriatric care settings [33].

#### Counseling and Psychosocial Intervention

Once initial needs are identified, patients and, if applicable, their relatives receive comprehensive counseling about planned measures and available support services. This includes both medical or nursing and psychosocial considerations. The goal is to jointly develop a realistic and sustainable care plan. If necessary, psychosocial interventions such as crisis counseling or referrals to support services are initiated.

#### Information From Patients and Clinical Staff

Close coordination with the medical and nursing teams is essential, especially when assessing therapeutic needs and defining the discharge timeline. The Barthel Index continues to play a supporting role in this phase. For planned interventions, patients may be contacted prior to admission to identify support needs early.

*Having the correct contact details is really important. Sometimes we end up playing detective, searching for phone numbers in other places. We may call someone listed as a relative and then realize it’s an ex-partner or someone completely different. That can lead to very uncomfortable situations*.

[Participant, Workshop 5, Hospital B]

#### Discharge Planning

Based on the data gathered, an individualized discharge plan is developed. This includes the patient’s care needs, living situation, and available resources. On average, plan preparation takes 6 hours per patient, although this varies with case complexity. The objective is to ensure a seamless transition and initiate necessary follow-up measures in a timely manner.

#### Implementation of DP Measures

In this phase, the planned measures are implemented in collaboration with other stakeholders. Textbox 5 outlines the key practical steps.

Textbox 5. **Key steps in implementing discharge planning as reported by participants during workshops and interviews.**

- Completing and submitting applications to cost bearers.
- Forwarding relevant documents to physicians.
- Sending requests to aftercare providers and other service organizations (eg, rehabilitation clinics and outpatient nursing services).
- Procuring medical equipment and supplies.
- Requesting and processing insurance approvals.

Timelines for these steps depend on insurance type, case complexity, and local care infrastructure. For patients residing farther from the hospital, planning often starts earlier. Ultimately, the discharge process is influenced not only by procedural tasks but also by the patient’s health status and personal preferences, requiring a high degree of coordination and adaptability.

#### Final Coordination and Discharge Preparation

Once all measures are initiated, a final coordination is conducted among stakeholders. At this point, inquiries to care providers have been sent, necessary applications submitted, and, where possible, cost approvals obtained. Patients and families are thoroughly briefed on the discharge date, aftercare arrangements, and the next steps. This ensures a well-coordinated and needs-oriented transition and minimizes uncertainty at the point of discharge.

### Factors Influencing the Success of DP

Multiple factors shape the success of DP in practice. Table 4 outlines these factors with examples.

**Table 4.** Factors influencing the success of discharge planning and implications for technology design.

Factor	Example/description	Technological implication
Staffing and personnel availability.	Staff shortages and high caseloads require workflow prioritization and can lead to placeholders (eg, “1234”) in software systems, necessitating retrospective data collection and additional coordination.	Systems should support workload balancing, task automation, and asynchronous collaboration.
Completeness and timeliness of information.	Needs assessments are not always systematic. Incomplete or delayed data require follow-up, prolong processing times, and may trigger care plan revisions.	Data validation and completeness checks should be embedded early in the workflow.
Departmental and professional practice variations.	Planning practices differ across departments. While some units begin early with standardized procedures, others (eg, surgical oncology) engage in planning only postdischarge. This heterogeneity complicates coordination.	Adaptive interfaces and modular workflows can accommodate heterogeneity while promoting best practices.
Time constraints and deadlines.	Legal and contractual obligations impose strict timing. Short notice discharges or complex cases require particularly proactive coordination.	Artificial intelligence–driven scheduling and prioritization tools can support deadline compliance.
Documentation and consent processes.	While patient consent is a formal prerequisite, it is not always consistently documented. Some patients are registered for follow-up care without a thorough needs assessment, leading to potential plan adjustments later.	Intelligent workflows should include reminders, status tracking, and plausibility checks.
Changing individual care needs.	Patient needs, especially in geriatric care, can shift during hospitalization. Continuous reassessment and flexible coordination are necessary.	Systems must support flexible updates and real-time documentation to reflect patient trajectories.
Availability of aftercare services.	Regional capacity limitations, particularly in rehabilitation, pose significant planning challenges. Long wait times and lack of preregistration options often require last-minute adjustments.	Integration with real-time availability directories and decision support for service matching is needed.

### Core Requirements for Technologies

The primary goal of DP professionals is to provide personalized, needs-based aftercare. Achieving this requires a comprehensive, up-to-date, and easily accessible information base. The entire discharge process hinges on the availability, quality, and timeliness of data. In this context, digital documentation and communication systems, especially when enhanced with AI, can play a crucial role in supporting this goal. Based on our findings, the following section outlines specific requirements for the design and implementation of such technologies in DP.

#### Communication and Notification

In terms of communication and notifications, hospital staff expressed a strong interest in AI-based support in general. One central need is automated alerts about critical developments in a patient’s treatment journey. This includes transfers, death, unplanned surgeries, or changes in surgical schedules. To tailor information flows to individual roles, staff should be able to personalize notification settings. AI could also be used to remind physicians of outstanding test results and automatically send those results, as well as other key documents such as prescriptions, to the relevant stakeholders. Our participants reported the particular importance of an automatic alert when a patient’s Barthel Index changes, helping ensure that shifts in care needs are promptly addressed.

*Once patients are discharged, they are no longer actively tracked in the system, even though we are often still working on their rehabilitation planning. For that, we frequently need medical reports. But physicians may not see these requests because they no longer reopen the digital record after discharge. An AI system could help by flagging this, for example, by notifying physicians: “Attention, reports still need to be completed.”*

[Participant, Workshop 5, Hospital B]

Patients could also benefit from automated reminders, both for initiating DP early and for upcoming appointments. A particularly innovative idea was the conversion of voicemail messages into text, delivered directly to staff email inboxes. This could significantly reduce time spent fielding routine inquiries via phone. AI could also sort incoming phone calls and DP requests by topic, enabling staff to respond more efficiently. Another promising feature would be the automatic routing of DP requests to the appropriate team or department, including forwarding relevant documents following contact with family members.

Staff also highlighted the need for automated alerts in cases where cost approvals for rehabilitation are still pending, a common bottleneck in the process. AI could also enhance transparency for patients and families by summarizing consultation discussions in writing, providing easy-to-understand takeaways. Similarly, sending automated introductory information could help reassure patients early in their stay. AI-generated reminders for appointments and tasks could also bring more structure to daily workflows. Additionally, AI systems could ensure that required documents are always current and help prepare consultation materials based on individual patient risk profiles.

#### Discharge and Aftercare Planning

Staff see significant potential for AI to improve discharge and aftercare planning by streamlining and targeting key processes. One major function would be the automated search for appropriate and available home care providers or nursing home placements. While software catering to this need exists, it is often dependent on licensing so that not all providers are listed. An automatically generated, continuously updated overview of available providers from freely available internet data would save staff time and effort both in research and in outreach.

Identifying aftercare needs early, especially for planned surgeries, was seen as vital for ensuring smooth transitions from inpatient treatment to posthospital care. AI could analyze individual needs from the data in electronic health records, suggest appropriate providers, show their availability, and even contact the relevant insurers. Ideally, this process begins automatically when the patient is first admitted, with AI refining its recommendations as clinical documentation progresses. This kind of autonomous planning could significantly reduce the administrative burden on staff. AI could also generate clinical orders automatically, either based on predefined criteria or upon staff request, provided all necessary information is available and there is a clear medical justification.

Error prevention was another important focus reported by our participants. AI systems should be able to detect and prevent duplicate discharge registrations for the same patient. Similarly, they should limit multiple or conflicting selections when specifying aftercare providers to avoid confusion and delays. The system should also flag unusual case types, for example, when patients younger than 65 years are registered for long-term care facilities. It could also ensure that document requirements for different stakeholders (such as payers or doctors) are kept up to date.

#### Data Management and Documentation

Staff also saw major opportunities for AI in improving data handling and documentation, ensuring that all required information is complete before initiating that DP is a key concern. AI could validate this automatically before allowing the process to proceed. To reduce repetitive work, the system should be able to transfer previously collected data when patients are readmitted. This would not only save time but also minimize errors from manual data entry. Similarly, AI could prefill or complete forms and applications using existing patient data.

*With rehabilitation applications, I usually don’t carry the forms with me because I don’t know yet whether the patient actually wants or needs them and I don't want to waste paper unnecessarily. It would be very practical to have a solution that allows me to access and complete those forms directly on the ward, as needed*.

[Participant, Workshop 6, Hospital B]

Another helpful feature would be automatic documentation such as logging when documents are issued. This would ensure a complete and traceable discharge process across different team members. Standardized text templates could also speed up routine documentation tasks such as report writing or formal communication. A concrete example is the automated generation of the Barthel Index when a DP order is initiated. To improve process quality, access to the patient record could be restricted unless the Barthel Index is present. This would help ensure that essential information is not missing. Staff also advocated for plausibility checks, particularly for the discharge report. AI could detect implausible entries and prompt users to review them. Likewise, the system should be able to verify whether cases marked as “urgent” actually meet the defined urgency criteria.

#### Needs Assessment and Clarification of Responsibilities

In the area of needs assessment and responsibility allocation, staff emphasized the value of AI in improving both efficiency and the quality of care. One core function would be the automatic identification of needs for medical aids. Based on patient data, AI could generate tailored suggestions and show which items are covered by insurance and available at local suppliers.

Automatically identifying the responsible payer would also reduce administrative effort and ensure a smooth flow of information. When planning rehabilitation, AI should also flag potential issues, such as homelessness or addiction, that might complicate discharge or exclude patients from certain programs. “If a physician selects both inpatient and outpatient care, the system should immediately block that. You should only be able to choose one or the other” (Participant, Workshop 6, Hospital B). Similarly, the system could enforce required isolation protocols and detect communication barriers, such as hearing loss or cognitive impairments. Automated reminders could help ensure that no essential information (eg, sensory or cognitive status) is overlooked.

Clarifying responsibilities within the hospital itself was another key area. AI could use existing data to assign cases to the right professional group or department and register patients accordingly. This would promote interdisciplinary collaboration and timely action. To further improve communication, AI could support needs assessment and automatically share relevant updates between social workers, patients, and their families via email. This would help ensure that everyone stays informed, while fostering transparency, participation, and mutual commitment.

Table 5 summarizes the key technological requirements identified in this study, directly addressing the main challenges influencing the success of DP. The requirements are organized to reflect how specific technological solutions can mitigate obstacles in communication, planning, data handling, and responsibility allocation. Requirements are derived from the integrated content analysis and categorized by the researchers.

**Table 5.** Derived categories from technological requirements addressing key discharge planning challenges.

Category	Core requirements for technology support
Communication and notification	Automated alerts for patient events (eg, transfers, deaths, and surgery changes). Personalized notification settings for staff. Reminders for outstanding test results and cost approvals. AI^a^-based sorting of phone calls and DP^b^ requests. Automated patient communications (eg, appointment reminders and consultation summaries).
Discharge planning of aftercare	Early identification of follow-up care needs. AI-assisted search for home care providers and nursing facilities. Automated creation of clinical orders based on defined criteria. Error prevention (eg, duplicate registrations and conflicting follow-up provider selections).
Data management and documentation	Validation of data completeness before initiating DP. Automatic transfer of existing patient data for readmissions. Prefilling forms and applications. Logging document issuance and using standardized text templates. Automatic generation and validation of Barthel Index.
Needs assessment and responsibility clarification	Automated identification of medical aid needs. Detection of barriers (eg, homelessness, addiction, and communication impairments). Automatic assignment of cases to appropriate departments or professional groups. AI-supported communication between social workers, patients, and families.

a AI: artificial intelligence.

b DP: discharge planning.

### Integration of Findings

The integration of qualitative and quantitative findings provided a more comprehensive understanding of DP practices. The qualitative data offered detailed insights into contextual mechanisms such as communication barriers, unclear responsibilities, and workflow interruptions. The quantitative results quantified the extent of these issues within the participating hospitals. For example, the high administrative workload frequently mentioned in workshops was reflected in the survey, where most respondents reported spending over half of their time on documentation and coordination tasks. Similarly, the qualitative emphasis on time pressure and limited standardization corresponded with lower satisfaction scores in the questionnaire regarding the adequacy of time and clarity of procedures. Together, these converging findings strengthen the interpretation that the challenges in DP are both systemic and measurable, and that improving information flow and responsibility structures should be a central focus in the design of AI-supported solutions.

## Discussion

### Principal Findings

This study aimed to explore structural challenges in DP and to identify context-specific requirements for AI-supported systems from the perspectives of health care professionals, patients, and relatives. The findings show that current DP practices are characterized by limited standardization, fragmented responsibility structures, and disrupted information flows. These challenges are primarily systemic rather than individual in nature and shape both the perceived need for digital support and the conditions under which AI-based solutions may be meaningfully integrated into clinical practice.

This study examined the structure and success factors of DP as well as the potential of AI-supported systems to assist this process. The results indicate that current DP practices rely heavily on individual expertise and situational decision-making and show limited standardization in the collection and documentation of patient data. At the core of the identified challenges are deficits in responsibility structures, documentation, and information flow—findings that indicate systemic weaknesses and align with previous studies [5,34].

### Systemic and Structural Challenges

Interpreting these findings, the data suggest that the observed difficulties in DP are not attributable to individual shortcomings but reflect broader structural fragmentation and insufficient interoperability within the German health care system. These findings are consistent with previous studies that describe DP as being hindered by fragmented responsibilities, insufficient information flow, and limited interoperability despite existing regulatory frameworks [5,34]. The multitude of involved stakeholders (hospital staff, external care providers, insurers, and patients or relatives) makes consistent information flow difficult. Despite existing regulatory frameworks (eg, DNQP), concrete procedural guidelines are lacking, resulting in uncertainty regarding responsibilities and coordination between the professions involved. This structural ambiguity means that the quality of DP largely depends on individual competence and experience instead of standardized, technology-supported procedures.

### Role and Requirements of AI-Based Technologies

Against this background, it becomes evident that AI-supported systems are not merely technological additions but can potentially assume a structural function within DP. The application areas identified in the study (communication and notification, planning, data management, and needs assessment) show that AI is suitable for addressing key bottlenecks at the microlevel. The discussion differentiates the specific functional requirements in the following sections.

### Technological Distinction (AI Versus Classical IT Systems)

Many of the proposed functions, such as automated alerts, configurable notification systems, or the forwarding of documents, do not necessarily require AI. They can also be implemented through rule-based logic in existing hospital information systems, electronic health records, or middleware solutions (“if A, then B”). However, the study shows that the practical reality in the examined health care institutions is characterized by fragmentation and limited interoperability: multiple parallel software solutions (nursing documentation, case management, billing, aftercare coordination, etc) rarely communicate seamlessly with one another [29]. Even when individual systems theoretically offer notification features, their usefulness remains limited without integration. Against this backdrop, the identified problem of insufficient digital documentation of patient data further exacerbates the issue and suggests that a solution should target the level of existing software systems.

AI becomes particularly relevant where pattern recognition, prediction, or context-dependent decisions are required, for example: (1) prioritization of alerts (“Which events are truly critical?”), (2) adaptive notification systems (“Who should receive information, depending on the case context?”), and (3) semantic data integration (“Recognition of similar cases or relevant aftercare providers across heterogeneous systems”).

In this sense, AI is not primarily the base function but a mechanism for intelligent networking, prioritization, and integration of existing systems.

### Communication and Notification

A recurring problem lies in delayed or interrupted information flows. Automated alerts about relevant changes in patient status, personalized notification systems, and the integration of voice-to-text functions could directly address these deficits. In particular, the automatic forwarding of relevant documents or reminders about pending test results to the responsible person was described by respondents as practical reliefs. Intelligent integration into existing systems could increase responsiveness of the DP team and close information gaps between professional groups.

### DP of Aftercare

Automating the search for aftercare providers and generating suggestions based on clinical documentation illustrate the potential to simplify complex coordination tasks. Adaptive systems that continuously integrate relevant data during treatment can prepare the discharge process early. This can bring efficiency gains and support error reduction through standardized plausibility checks and provide cognitive relief for staff involved in the process.

### Data Management and Documentation

Redundant data entry and incomplete documentation burden staff and delay processes. This knowledge has been confirmed in other literature [21]. AI-supported mechanisms such as automatic completeness checks, prefilling of forms, or standardized report texts can save time and improve documentation quality. The ability to accelerate readmissions through data transfer highlights this potential. At the same time, the results emphasize that technological systems must be integrated into existing hospital IT infrastructures to be effective. These observations reinforce existing evidence that redundant documentation and incomplete data entry are major contributors to inefficiencies in discharge processes [21], while further illustrating how automation and AI-supported checks could mitigate these issues in practice.

### Needs Assessment and Responsibilities

AI can identify risks or needs at an early stage and automatically assign cases to the appropriate professional groups. Automatic detection of rehabilitation needs, medical aids, or social risk factors could support in preventing bottlenecks and clarify responsibilities while also increasing transparency.

### Social and Cultural Prerequisites

Despite the potential, the introduction of AI-based systems and their acceptance by staff remain challenging. Employees expressed both openness and skepticism toward digital support, particularly regarding learning effort, the adaptation of work routines, and possible loss of control. This ambivalence points to cultural dynamics: a strongly profession-oriented work culture, high demands for autonomy, and limited management strategies for digital transformation [29,35]. AI systems must therefore be not only technically compatible but also socially integrative, meaning that they must respect and support existing work logics. By empirically grounding these dynamics in the context of DP, the study contributes to existing discussions on why digital transformation efforts in hospitals often encounter resistance despite perceived usefulness.

### Implications for the Design of Digital Systems

Participatory design processes are crucial: end users, especially nurses, social services, and physicians, must be involved early in the development process. Systems must be intuitive to use, transparent, and operable with minimal training effort. Technical prerequisites such as fully digitalized hospital infrastructure and interoperable data systems form the foundation for successful implementation.

In line with prior research on digital transformation in health care, the findings underscore that AI-based systems unfold their potential only when aligned with organizational structures, professional cultures, and interoperable infrastructures. This study demonstrates that technological innovation in DP can be effective only when embedded within organizational, infrastructural, and cultural contexts. Beyond identifying specific functional requirements, the findings underscore the importance of sociotechnical alignment as a prerequisite for sustainable digital transformation in health care. AI can standardize processes, improve information flows, and automate routine tasks, but it does not replace professional judgment or interpersonal care. Rather, it enables professionals to focus more on patient-centered tasks. Without simultaneous consideration of infrastructural, organizational, and cultural prerequisites, even technically advanced AI systems risk failing to unfold their potential in DP. These aspects are central to the successful implementation of AI-supported systems in DP.

### Limitations

This study has several limitations. Participation among health care professionals was constrained by staffing pressures and clinical workload, which limited the number of interview and workshop participants. While common in applied hospital research, these conditions also reflect the environments in which AI-supported DP tools must ultimately operate. Additionally, a limited sample size and single-site interview data restrict representativeness across professional roles and institutions. The findings should therefore be interpreted as context-specific, offering in-depth, practice-based insights rather than generalizable results. The quantitative survey primarily complemented the qualitative data; 1 domain (satisfaction with procedural aspects of DP) was collected at only 1 site due to ethical constraints. The study prioritized contextual understanding over statistical breadth. While the findings are not generalizable to all hospital environments, they extend sociotechnical and technology acceptance perspectives by empirically linking organizational context, workload structures, and attitudes toward AI-enabled DP.

### Future Work

Future research should differentiate between AI functionalities that are technically feasible within current hospital IT infrastructures and those requiring broader organizational or regulatory development. While features such as automated alerts, structured documentation, and notification systems can be implemented under existing conditions, more advanced applications such as predictive matching or adaptive prioritization depend on improved data interoperability, AI maturity, and clear governance frameworks. Additionally, investigations into operational outcomes such as time-to-discharge or readmission rates would provide important evidence of real-world impact. Future evaluation studies, ideally linked to pilot implementations, should include such indicators to measure the effectiveness of AI-supported systems. Although this research was conducted in the German health care context, challenges such as fragmented communication and limited digital integration are common across Western health care systems. Comparative studies could examine how these findings translate to other national and organizational settings, supporting the development of context-sensitive strategies for integrating AI into discharge management.

### Conclusions

This study highlights the complex challenges health care professionals face in coordinating seamless transitions from hospital to aftercare. Despite established standards and regulatory frameworks, DP remains dependent on individual expertise, ad hoc coordination, and the timely availability of information. The findings identify clear opportunities for AI-supported tools, particularly in improving communication, data integration, documentation, and individualized care planning. However, success depends on robust digital infrastructures and high-quality, interoperable data. AI systems that automate administrative tasks and improve access to case-relevant information can alleviate workload and enhance the efficiency and accuracy of discharge processes. Crucially, this allows health care professionals to focus more on patient-centered tasks that require empathy, judgment, and interpersonal care. Future development should emphasize participatory design and real-world evaluation to ensure that digital innovations genuinely enhance continuity of care, staff experience, and patient outcomes.

## Supplementary material

10.2196/81824 Multimedia Appendix 1 Disclosure of artificial intelligence usage.

10.2196/81824 Multimedia Appendix 2 Interview guide for physicians.

10.2196/81824 Multimedia Appendix 3 Interview guide for nurses.

10.2196/81824 Multimedia Appendix 4 Interview guide for senior physician.

10.2196/81824 Multimedia Appendix 5 Questionnaire used.

10.2196/81824 Multimedia Appendix 6 Coding system.

10.2196/81824 Multimedia Appendix 7 Tabular overview of coded segments.

10.2196/81824 Multimedia Appendix 8 Overview of statements sorted by workshops.

10.2196/81824 Multimedia Appendix 9 Flowchart of the discharge planning process.

10.2196/81824 Checklist 1 Checklist for GRAMMS (Good Reporting of a Mixed Methods Study).

10.2196/81824 Checklist 2 Checklist for MMR-RHS (Mixed Methods Reporting in Rehabilitation & Health Sciences).

## Abbreviations

AI: artificial intelligence
DNQP: German Network for Quality Development in Nursing
DP: discharge planning
GRAMMS: Good Reporting of a Mixed Methods Study
MMR-RHS: Mixed Methods Reporting in Rehabilitation & Health Sciences
SGB V: Fifth Book of the German Social Code
