# Supplementary material for: Optimizing Hospital Discharge Planning: Empirical Insights and Requirements of AI-Based Technologies From an Explorative Mixed Methods Field Study
Source: JMIR Form Res. 2026 Mar 24;10:e81824. doi: 10.2196/81824 (PMC13012232; doi:10.2196/81824)

Disclosure AI usage

Using ChatGPt to create a prompt for generating a flowchart

PROMPT:

Erstelle anhand des von mir eingegebenen Textes einer Prozessbeschreibung einen Prompt für eine generative KI. Dieser Prompt soll die wesentlichen Schritte, Meilensteine und Schlüsselereignisse aus der Prozessbeschreibung enthalten, sodass die KI daraus einen Entwurf einer Flowchart erstellen kann. Hier ist der Text:

"Participants described the DP process as a reactive and highly individualized response to the situational demands of daily hospital operations. According to their accounts, the implementation of DP often depends heavily on the expertise, experience, and judgment of the individual staff members involved. Although a standard for DP is provided by the German Network for Quality Development in Nursing (DNQP), it primarily defines general phases and criteria rather than prescribing a concrete procedural framework. DNQP is a German network that develops expert standards to promote quality improvement in nursing practice [32]. While widely recognized and used as a reference in quality assurance, the DNQP standard remains advisory rather than legally binding, offering guidance rather than strict regulation. Moreover, neither the legal frameworks defined in the introduction nor internal organizational guidelines provide a detailed, binding sequence of steps. Instead, certain key objectives such as timely DP, interdisciplinary coordination, and continuity of care must be achieved. These pillars aim to ensure legal compliance while allowing for flexible adaptation to the individual needs of patients.

In light of this flexibility, and in agreement with participants, the decision was made to present a best-case scenario for DP as there is no universally standardized process across departments. This model is grounded in shared structural elements and clearly defined steps, forming a general process framework that supports effective and patient-centered DP. Beyond its descriptive function, the model serves as a design-oriented artifact: it captures practitioners' normative expectations of how DP should function under optimal conditions. As such, it provides a valuable reference point for identifying friction points in current practices and for mapping potential entry points for AI-supported interventions. This best case framing allows for a participatory, practice-based foundation to guide system design in line with real-world workflows and professional logics.

The following sections outline the key components of this idealized model. A best-case process model can be found in the multimedia appendix.

Initiating discharge planning

The DP process typically begins with a clinical referral issued by medical staff. This involves formally requesting the services of the hospital's DP team to coordinate necessary post-discharge care. A referral is made when a medical, therapeutic, or nursing need for aftercare has been identified. According to the DNQP standard, a criteria-based initial assessment must occur within 24 hours of assuming responsibility for the patient's nursing care. This is usually performed by medical or nursing staff and serves as the formal prerequisite for initiating DP. Referrals may be issued by nursing staff, physicians, or directly by patients or their legal representatives. In practice, however, this process often deviates from the standard due to time constraints, limited systematic assessments during admission, and insufficient interdisciplinary communication. Once a need for aftercare is identified and a referral is made, patients or their legal representatives are informed and receive counseling. Written consent is required to proceed, and no further steps may be taken without it. Even in cases of objective need, not all patients choose to participate. Once consent is provided, a detailed needs assessment follows.

Needs assessment and initial contact

At this stage, the social services and nursing transition teams assess the patient's individual aftercare needs. This involves reviewing the medical record and conducting a personal conversation with the patient and/or relatives to gather contextual information on the home environment, care situation, available resources, and support systems.

To plan appropriate aftercare, DP staff require comprehensive insights into the patient's broader living context. Table 4 outlines key categories of discharge-relevant information. Among these, contact information is particularly crucial for enabling communication. Early clarification of legal competence and cognitive capacity is also important. Ideally, communication challenges such as hearing or speech impairments are identified early. If a care dependency level was previously established, it should be documented at admission to streamline planning. The DP team also consults with physicians and nursing staff to estimate the discharge date and assess the need for follow-up services. A commonly used assessment tool is the Barthel Index, which evaluates a patient's ability to perform basic activities of daily living and supports care planning. The Barthel Index is often applied in rehabilitation and geriatric care settings [33].

Counseling and psychosocial intervention

Once initial needs are identified, patients and, if applicable, their relatives receive comprehensive counseling about planned measures and available support services. This includes both medical/nursing and psychosocial considerations. The goal is to jointly develop a realistic and sustainable care plan. If necessary, psychosocial interventions such as crisis counseling or referrals to support services are initiated.

Information from patients and clinical staff

Close coordination with the medical and nursing teams is essential, especially when assessing therapeutic needs and defining the discharge timeline. The Barthel Index continues to play a supporting role in this phase. For planned interventions, patients may be contacted prior to admission to identify support needs early.

Discharge planning

Based on the data gathered, an individualized discharge plan is developed. This includes the patient’s care needs, living situation, and available resources. On average, plan preparation takes six hours per patient, though this varies with case complexity. The objective is to ensure a seamless transition and initiate necessary follow-up measures in a timely manner.

Implementation of discharge planning measures

In this phase, the planned measures are implemented in collaboration with other stakeholders. Table 5 outlines the key practical steps.

Timelines for these steps depend on insurance type, case complexity, and local care infrastructure. For patients residing farther from the hospital, planning often starts earlier. Ultimately, the discharge process is influenced not only by procedural tasks but also by the patient’s health status and personal preferences, requiring a high degree of coordination and adaptability.

Final coordination and discharge preparation

Once all measures are initiated, a final coordination is conducted among stakeholders. At this point, inquiries to care providers have been sent, necessary applications submitted, and, where possible, cost approvals obtained. Patients and families are thoroughly briefed on the discharge date, aftercare arrangements, and the next steps. This ensures a well-coordinated and needs-oriented transition and minimizes uncertainty at the point of discharge.

ANSWER ChatGPT:

Unten findest du einen **formulierten Prompt**, der die wesentlichen Schritte, Meilensteine und Schlüsselereignisse der Prozessbeschreibung enthält und von einer generativen KI genutzt werden kann, um **einen Entwurf eines Flowcharts** zu erstellen.

Prompt für generative KI (Flowchart-Entwurf)

Erstelle ein Flowchart, das den idealisierten Best-Case-Prozess der klinischen Entlassungsplanung (Discharge Planning, DP) darstellt. Nutze die folgenden Prozessschritte, Meilensteine und Schlüsselereignisse:

1. Prozessstart: Initiierung der Entlassungsplanung

- Klinische Überweisung/Referral durch medizinisches oder pflegerisches Personal, Patienten oder rechtliche Vertreter.
- Anlass: Identifizierung eines medizinischen, therapeutischen oder pflegerischen Nachsorgebedarfs.
- **Meilenstein:** Kriterienbasierte Ersteinschätzung innerhalb von 24 Stunden nach Übernahme der Pflege (gemäß DNQP-Standard).
- Patienten/Angehörige werden informiert und beraten.
- **Schlüsselereignis:** Einholen der schriftlichen Einwilligung (ohne Consent keine Fortsetzung).

2. Bedarfserhebung und Erstkontakt

- Einschätzung individueller Nachsorgebedarfe durch Sozialdienst und Pflegetransitionsteams.
- Prüfung der Patientenakte; persönliches Gespräch mit Patient/Angehörigen zu Wohnsituation, Ressourcen, Unterstützungsnetzwerken.
- Frühe Klärung von:
    - Kontaktinformationen
    - rechtlicher Vertretung
    - kognitiver Fähigkeiten
    - Kommunikationsbarrieren (Hören/Sprechen)
    - vorhandener Pflegegrad

- Rücksprache mit Ärzten und Pflegepersonal zur Entlassungsprognose und zu erforderlichen Leistungen.
- Nutzung des **Barthel Index** zur Einschätzung der Alltagskompetenz.

3. Beratung und psychosoziale Intervention

- Umfassende Beratung von Patienten und ggf. Angehörigen zu geplanten Maßnahmen und verfügbaren Unterstützungsangeboten.
- Gemeinsame Entwicklung eines realistischen, nachhaltigen Nachsorgeplans.
- Bei Bedarf: psychosoziale Interventionen (z. B. Krisenberatung, Vermittlung von Unterstützungsdiensten).

4. Informationsaustausch mit Patient und klinischen Teams

- Enge Abstimmung mit medizinischem und pflegerischem Team (therapeutische Bedarfe, Entlassungstermin).
- Fortgesetzte Nutzung des Barthel Index zur Bedarfsklärung.
- Bei planbaren Eingriffen: mögliche frühzeitige Kontaktaufnahme vor stationärer Aufnahme.

5. Erstellung des Entlassungsplans

- Zusammenführung aller Informationen zur Entwicklung eines individuellen Entlassungsplans.
- Inhalte: Pflegebedarf, Wohnsituation, vorhandene Ressourcen, notwendige Leistungen.
- Durchschnittlicher Zeitaufwand: ca. 6 Stunden (variiert nach Fallkomplexität).
- Ziel: Sicherstellung eines reibungslosen Übergangs in die Nachsorge.

6. Umsetzung der Entlassungsmaßnahmen

- Umsetzung der geplanten Schritte in Kooperation mit internen und externen Akteuren.
- Wichtige Aufgaben (Beispiele aus Prozesslogik):
    - Kontaktaufnahme zu Pflegediensten
    - Organisation von Hilfsmitteln
    - Antragstellungen
    - Abstimmung mit Kostenträgern
- Zeitliche Abfolge variiert je nach Fall, Versicherungsart, regionaler Versorgungsstruktur.
- Flexibilität aufgrund von Gesundheitszustand und Präferenzen des Patienten erforderlich.

7. Abschlusskoordination und Entlassvorbereitung

- Finaler Abgleich aller eingeleiteten Maßnahmen zwischen allen Stakeholdern.
- Sicherstellung, dass:
    - Anfragen an Leistungserbringer verschickt sind
    - Anträge eingereicht wurden
    - Kostenübernahmen (soweit möglich) vorliegen

- Information des Patienten/der Angehörigen über Entlassdatum, Nachsorgeleistungen und nächste Schritte.
- Ziel: Sicherer, verständlicher und bedarfsgerechter Übergang.

8. Prozessende: Entlassung

Erstelle auf Basis dieser Schritte ein übersichtliches Flowchart, das die logische Reihenfolge, Abhängigkeiten, Entscheidungspunkte und Meilensteine des Best-Case-DP-Prozesses darstellt.

Using this prompt to generate a flowchart with lucid.ai (lucid.app)

PROMPT:

Erstelle ein Flowchart, das den idealisierten Best-Case-Prozess der klinischen Entlassungsplanung (Discharge Planning, DP) darstellt. Nutze die folgenden Prozessschritte, Meilensteine und Schlüsselereignisse:

1. Prozessstart: Initiierung der Entlassungsplanung

- Klinische Überweisung/Referral durch medizinisches oder pflegerisches Personal, Patienten oder rechtliche Vertreter.
- Anlass: Identifizierung eines medizinischen, therapeutischen oder pflegerischen Nachsorgebedarfs.
- Meilenstein: Kriterienbasierte Ersteinschätzung innerhalb von 24 Stunden nach Übernahme der Pflege (gemäß DNQP-Standard).
- Patienten/Angehörige werden informiert und beraten.
- Schlüsselereignis: Einholen der schriftlichen Einwilligung (ohne Consent keine Fortsetzung).

2. Bedarfserhebung und Erstkontakt

- Einschätzung individueller Nachsorgebedarfe durch Sozialdienst und Pflegetransitionsteams.
- Prüfung der Patientenakte; persönliches Gespräch mit Patient/Angehörigen zu Wohnsituation, Ressourcen, Unterstützungsnetzwerken.
- Frühe Klärung von:
    - o Kontaktinformationen
    - o rechtlicher Vertretung
    - o kognitiver Fähigkeiten
    - o Kommunikationsbarrieren (Hören/Sprechen)
    - o vorhandener Pflegegrad
- Rücksprache mit Ärzten und Pflegepersonal zur Entlassungsprognose und zu erforderlichen Leistungen.
- Nutzung des Barthel Index zur Einschätzung der Alltagskompetenz.

3. Beratung und psychosoziale Intervention

- Umfassende Beratung von Patienten und ggf. Angehörigen zu geplanten Maßnahmen und verfügbaren Unterstützungsangeboten.
- Gemeinsame Entwicklung eines realistischen, nachhaltigen Nachsorgeplans.
- Bei Bedarf: psychosoziale Interventionen (z. B. Krisenberatung, Vermittlung von Unterstützungsdiensten).

4. Informationsaustausch mit Patient und klinischen Teams

- Enge Abstimmung mit medizinischem und pflegerischem Team (therapeutische Bedarfe, Entlassungstermin).
- Fortgesetzte Nutzung des Barthel Index zur Bedarfsklärung.
- Bei planbaren Eingriffen: mögliche frühzeitige Kontaktaufnahme vor stationärer Aufnahme.

5. Erstellung des Entlassungsplans

- Zusammenführung aller Informationen zur Entwicklung eines individuellen Entlassungsplans.
- Inhalte: Pflegebedarf, Wohnsituation, vorhandene Ressourcen, notwendige Leistungen.
- Durchschnittlicher Zeitaufwand: ca. 6 Stunden (variiert nach Fallkomplexität).
- Ziel: Sicherstellung eines reibungslosen Übergangs in die Nachsorge.

6. Umsetzung der Entlassungsmaßnahmen

- Umsetzung der geplanten Schritte in Kooperation mit internen und externen Akteuren.
- Wichtige Aufgaben (Beispiele aus Prozesslogik):
  - Kontaktaufnahme zu Pflegediensten
  - Organisation von Hilfsmitteln
  - Antragstellungen
  - Abstimmung mit Kostenträgern
- Zeitliche Abfolge variiert je nach Fall, Versicherungsart, regionaler Versorgungsstruktur.

- Flexibilität aufgrund von Gesundheitszustand und Präferenzen des Patienten erforderlich.

7. Abschlusskoordination und Entlassvorbereitung

- Finaler Abgleich aller eingeleiteten Maßnahmen zwischen allen Stakeholdern.
- Sicherstellung, dass:
  - Anfragen an Leistungserbringer verschickt sind
  - Anträge eingereicht wurden
  - Kostenübernahmen (soweit möglich) vorliegen
- Information des Patienten/der Angehörigen über Entlassdatum, Nachsorgeleistungen und nächste Schritte.
- Ziel: Sicherer, verständlicher und bedarfsgerechter Übergang.

8. Prozessende: Entlassung

ANSWER lucid.app:

Das Flowchart für den idealisierten Best-Case-Prozess der klinischen Entlassungsplanung wurde erfolgreich erstellt. Es umfasst alle Prozessschritte, Meilensteine und Schlüsselereignisse, die Sie angegeben haben.

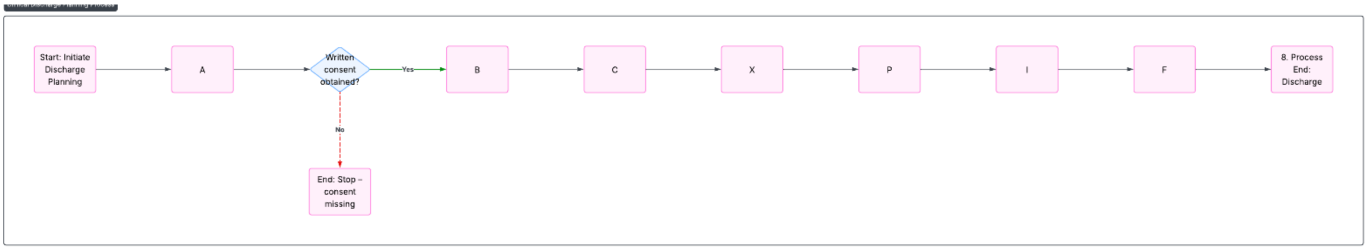

Supplement: Multimedia Appendix 1 [file formative-v10-e81824-s001.pdf]
