# Supplementary material for: Optimizing Hospital Discharge Planning: Empirical Insights and Requirements of AI-Based Technologies From an Explorative Mixed Methods Field Study
Source: JMIR Form Res. 2026 Mar 24;10:e81824. doi: 10.2196/81824 (PMC13012232; doi:10.2196/81824)
Supplement: Multimedia Appendix 2 [file formative-v10-e81824-s002.pdf]

Fragen Interview

1. Wie sieht der typische Ablauf auf Station bei der Nachsorgeplanung von Anfang bis Ende aus? (Was Sie das auf Station machen)

2. Auf welchen Informationen / Daten (in einer Patientenakte) stützen Sie Ihre Entscheidungen für die Entlassplanung?

3. Wie wird ein Auftrag für die Nachsorgeplanung aufgegeben? Wer erstellt den KLAU im System? Wer ordnet die Nachsorgeplanung an?

4. Inwiefern sind Sie mit dem PSZ in Kontakt oder wird nur ein KLAU aufgegeben?

5. Wie ist es, wenn es zu Änderungen bei der Entlassung kommt? Wann fallen diese auf?

6. Wie funktioniert in solchen Fällen die interne Kommunikation und die Kommunikation mit Patienten und Angehörigen?

7. Problemstellen: Wo kommt es zu Reibungspunkten? Wie reagieren Sie?
