# Supplementary material for: Optimizing Hospital Discharge Planning: Empirical Insights and Requirements of AI-Based Technologies From an Explorative Mixed Methods Field Study
Source: JMIR Form Res. 2026 Mar 24;10:e81824. doi: 10.2196/81824 (PMC13012232; doi:10.2196/81824)
Supplement: Multimedia Appendix 3 [file formative-v10-e81824-s003.pdf]

Frage Pflegeleitung (geplant für November, 2023)

1. Um die Nachsorgeplanung besser zu verstehen, würden wir gern darüber erfahren, wie die Prozesse aus Ihrer Sicht aussehen. Wie sieht der typische Ablauf auf Station bei der Nachsorgeplanung von Anfang bis Ende aus? Was passiert sobald das Entlassmanagement eingeschaltet wird? Wie sieht ein typisches Beispiel aus? Erzählen Sie erst mal!

2. Auf welchen Informationen / Daten stützen Sie Ihre Entscheidungen für die Nachsorgeplanung?

3. Wie wird ein Auftrag für die Nachsorgeplanung aufgegeben? Wer erstellt den KLAU im System? Wer ordnet die Nachsorgeplanung an?

4. Inwiefern sind Sie mit dem Pflegeservicezentrum in Kontakt?

5. Wie ist es, wenn es zu Änderungen bei der Entlassung kommt? Wann fallen diese auf? Wie wird mit Änderungen umgegangen?

6. Wo kommt es zu Reibungspunkten? Wie reagieren Sie? Wenn es Probleme gab: War etwas besonders hilfreich?

7. Wenn Sie an Ihre typischen Arbeitsabläufe zurückdenken, wo würde aus Ihrer Sicht ein KI-gestützten Assistenzsystem helfen, bzw. welche Abläufe können dadurch unterstützt werden?

8. Spezifische Fällen: Wie oft kommt ein Delir vor? Ist das entlassungsrelevant? Inwiefern sind die GKB Patienten im System markiert und wer trägt dies ein?
