# Supplementary material for: Optimizing Hospital Discharge Planning: Empirical Insights and Requirements of AI-Based Technologies From an Explorative Mixed Methods Field Study
Source: JMIR Form Res. 2026 Mar 24;10:e81824. doi: 10.2196/81824 (PMC13012232; doi:10.2196/81824)
Supplement: Multimedia Appendix 4 [file formative-v10-e81824-s004.pdf]

Fragen Oberarzt: Donnerstag 11.01.2024

- Um die Nachsorgeplanung besser zu verstehen, würden wir gerne mehrdarüber erfahren, wie die Prozesse aus Ihrer Sicht aussehen. Auf welche Weise sind Sie in den Entlassungsmanagementprozess eingebunden?

- Könnten Sie uns bitte mehr über die Rolle der Ärzte, des Pflegepersonals und der Sozialarbeiter im Entlassungsmanagementprozess erzählen? Was sind die Hauptaufgaben jeder dieser Gruppen bei der Entlassungsplanung?

- Auf welchen Informationen / Daten (in einer Patientenakte) stützen Sie Ihre Entscheidungen für die Entlassplanung?

- Was sind typische Probleme, die bei der Entlassungsplanung auftreten? Was sind die Ursachen für diese Probleme?

- Könnten Sie näher auf Probleme eingehen, die durch strukturelle/kommunikative Probleme zwischen dem Personal (Ärzte, Pflegepersonal und Sozialarbeitern) verursacht werden?

- Wenn Sie an die von Ihnen genannten Beispiele für Probleme in den welche en zurückdenken, wo würde Ihrer Meinung nach ein KI-gestütztes Assistenzsystem helfen bzw. welche Prozesse könnten dadurch unterstützt/vereinfacht werden?
