# Supplementary material for: Optimizing Hospital Discharge Planning: Empirical Insights and Requirements of AI-Based Technologies From an Explorative Mixed Methods Field Study
Source: JMIR Form Res. 2026 Mar 24;10:e81824. doi: 10.2196/81824 (PMC13012232; doi:10.2196/81824)
Supplement: Multimedia Appendix 5 [file formative-v10-e81824-s005.pdf]

Fragebogen für Mitarbeiter:innen

KIAFlex

Interaktive **KI-A**ssistenz zur prädiktiven und **flex**iblen Steuerung im Entlass- und Überleitungsmanagement: Erkundungsstudie
(KIAFlex-Explore)

In unserem Projekt **KIAFlex-Explore** wollen wir herausfinden, wie Sie als Mitarbeiter:innen zu einem künstliche Intelligenz (KI)-gestützten Entlassmanagement stehen. Mit dieser Basiserhebung wollen wir erste Einblicke in Ihre Arbeitsabläufe, Zufriedenheit und Perspektiven auf zukünftige KI-gestützte Lösungen sammeln, um diese von Anfang an in den Entwicklungsprozess einfließen zu lassen. Langfristig soll so die Kontinuität der Versorgung und die Kommunikation zwischen den verschiedenen involvierten Akteur:innen im Entlass- bzw. Überleitungsprozess verbessert werden.

Jetzt bitten wir Sie, als beteiligte Akteur:innen, die unten stehenden Fragen zu beantworten. Bei der Beantwortung kommt es allein auf Ihren persönlichen Eindruck an. Sie werden erst allgemein zu Ihrer Arbeit befragt. Anschließend folgen Fragen zum Thema KI und die Anwendung von KI-basierten Assistenzsytemen im Arbeitsalltag. Die Teilnahme ist freiwillig, Ihre Daten bleiben anonym und werden vertraulich behandelt.

Bitte füllen Sie den Fragebogen aus und schicken ihn bis **spätestens ***** mit dem beigefügten Hauspostumschlag an uns zurück. Alternativ können Sie den ausgefüllten Fragebogen auch per E-Mail an natalievictoria.grant@umm.de schicken.

Bei Fragen wenden Sie sich gerne an:

Frau Natalie Grant (natalievictoria.grant@umm.de, Telefon: 0621/383 3853)
Frau Tina Obenauer (tina.obenauer@umm.de, Telefon: 0621/383 3987)

Und los geht's mit der Befragung:

In welchem Bereich sind Sie tätig?

☐ Sozialdienst ☐ Pflegeüberleitung

☐ Abteilung Entlassmanagement ☐ Pflegestation

Bitte kreuzen ☒ Sie <u>eine</u> Antwort pro Frage an.
Sie können Ihre Antwort ändern, in dem Sie die falsche Antwort umranden.

**Arbeitsaufteilung**				
	**0-25%**	**26-50%**	**51-75%**	**76-100%**
Wie viel Prozent Ihrer Arbeitszeit verbringen Sie am Telefon?	☐	☐	☐	☐
Wie viel Prozent Ihrer Arbeitszeit verbringen Sie am Computer?	☐	☐	☐	☐
Wie viel Prozent Ihrer Arbeitszeit verbringen Sie mit Faxen?	☐	☐	☐	☐
Wie viel Prozent Ihrer Arbeitszeit verbringen Sie mit Verwaltungsaufgaben?	☐	☐	☐	☐
Wie viel Prozent Ihrer Arbeitszeit verbringen Sie mit der Erfassung von Dokumentationen, um den Nachsorgebedarf daraus abzuleiten?	☐	☐	☐	☐
Wie viel Prozent Ihrer Arbeitszeit verbringen Sie in der Rücksprache mit anderen Mitarbeiter:innen, um die Behandlung und Nachsorge von Patient:innen abzuklären?	☐	☐	☐	☐

**Zufriedenheit**					
	**trifft voll und ganz zu**	**trifft eher zu**	**trifft eher nicht zu**	**trifft über-haupt nicht zu**	**keine Mei-nung**
Ist die Zeit, die Sie für die Planung einer Entlassung habe, oft zu kurz?	☐	☐	☐	☐	☐
Denken Sie, dass die Überleitungsplanung unproblematisch läuft?	☐	☐	☐	☐	☐
Haben Sie das Gefühl, dass es einen Mangel an Kommunikation zwischen anderen Mitarbeiter:innen (Ärzt:innen, Pflegepersonal) während der Entlassungsplanung gibt?	☐	☐	☐	☐	☐
Fühlen Sie sich unter Druck von den behandelnden Ärtz:innen gesetzt, die Entlassung von Patient:innen in kurzer Zeit zu organisieren?	☐	☐	☐	☐	☐
Machen Sie viele Dinge im Arbeitsverlauf parallel?	☐	☐	☐	☐	☐
Würden Sie sagen, dass es zeitaufwändig ist, sich über die verfügbaren kommunalen Pflegdienste/Pflegeheime zu informieren?	☐	☐	☐	☐	☐
Erhalten Sie Entlassungsanmeldungen zeitnah?	☐	☐	☐	☐	☐
Denken Sie, dass die Entlassungsplanung unproblematisch läuft?	☐	☐	☐	☐	☐
Läuft die Kommunikation zwischen anderen Mitarbeiter:innen (Ärzt:innen, Pflegepersonal) während der Entlassungsplanung gut?	☐	☐	☐	☐	☐
Bekommen Sie zeitnahe Rückmeldung von anderen Mitarbeiter:innen (Ärzt:innen,	☐	☐	☐	☐	☐

Zufriedenheit					
Pflegepersonal), wenn sich etwas mit der Überleitungsplanung verändert?					
Haben Sie eine gute Kommunikation mit den behandelnden Ärzt:innen, wenn Sie die Überleitung in der Nachsorge von Patient:innen organisiere?	☐	☐	☐	☐	☐
Haben Sie genug Zeit, eine Überleitung in der Nachsorge zu planen?	☐	☐	☐	☐	☐
Erhalten Sie Entlassungsanmeldungen zu spät?	☐	☐	☐	☐	☐

Offene Fragen

Sie können auf die Rückseite des Fragebogen schreiben, wenn Sie mehr Platz brauchen.

Was würde Ihrer Meinung nach Ihre Arbeit erleichtern?

____

____

____

Was ist Ihrer Meinung nach die größte Barriere zur rechtzeitigen Entlassung?

____

____

____

Haben Sie andere Anregungen?

____

____

____

____

KI-basierte Assistenzsysteme tun genau das, sie „unterstützen“ den Arbeitsablauf, indem sie die Aufgabenbearbeitung anleiten oder immer wiederkehrende Aufgaben abnehmen. Was sie nicht tun, ist eine vollständige Erledigung aller Ihrer Arbeitsaufgaben.

**Erwartungen und Befürchtungen**					
	**ja**	**eher ja**	**eher nein**	**nein**	**keine Mei-nung**
Wären Sie bereit, ein KI-basiertes Assistenzsystem im Arbeitsverlauf auszuprobieren?	☐	☐	☐	☐	☐
Denken Sie, dass ein KI-basiertes Assistenzsystem Ihre Arbeit erleichtern könnte?	☐	☐	☐	☐	☐
Wenn man die Sicherheit der Patientendaten berücksichtigt, ist der Einsatz von KI im Entlassmanagement Ihrer Meinung nach ethisch vertretbar?	☐	☐	☐	☐	☐
Denken Sie, dass das Nutzen von einem KI-basierten Assistenzsystem Ihren Arbeitsaufwand erhöhen könnte?	☐	☐	☐	☐	☐
Befürchten Sie, dass durch die Nutzung von KI-basierten Assistenz-systemen Stellen eingespart werden könnten?	☐	☐	☐	☐	☐
Halten Sie den Einsatz von KI im Gesundheitswesen für ethisch problematisch?	☐	☐	☐	☐	☐
Denken Sie, dass KI eine nützliche Technologie ist?	☐	☐	☐	☐	☐
Denken Sie, dass ein KI-basiertes Assistenzsystem eine negative	☐	☐	☐	☐	☐

Erwartungen und Befürchtungen					
Auswirkung auf die Arzt-Patienten-Beziehung haben könnte?					
Denken Sie, dass ein KI-basiertes Assistenzsystem die Kommunikation zwischen Patient:innen und anderen Pflegegruppen verbessern könnte?	☐	☐	☐	☐	☐
Denken Sie, dass durch ein KI-basiertes Assistenzsystem Pflegeziele schneller erreicht werden könnten?	☐	☐	☐	☐	☐
Denken Sie, dass durch ein KI-basiertes Assistenzsystem Pflegeziele in einer höheren Qualität erreicht werden könnten?	☐	☐	☐	☐	☐

Offene Fragen

Welche Aufgaben sollten Ihrer Meinung nach außerhalb des KI-basierten Assistenzsystems verbleiben?

____

____

____

Was sind Ihre Ängste oder Unsicherheiten bezüglich der Anwendung eines KI-Systems in Ihrem Arbeitsalltag?

____

____

____

Haben Sie andere Anregungen?

____

____

____

____

Geschafft!

Vielen Dank für Ihre Teilnahme
