# Supplementary material for: Optimizing Hospital Discharge Planning: Empirical Insights and Requirements of AI-Based Technologies From an Explorative Mixed Methods Field Study
Source: JMIR Form Res. 2026 Mar 24;10:e81824. doi: 10.2196/81824 (PMC13012232; doi:10.2196/81824)
Supplement: Multimedia Appendix 6 [file formative-v10-e81824-s006.pdf]

Coding system				
**Upper category**	**Category**	**Definition**	**Subcategory**	**Manifestations/ Aspects**
**Artifical Intelligence**	**Expectations of AI**	Statements on the respondents' hopes and wishes regarding how AI can support them in their professional activities. In particular, expectations regarding increased efficiency, reduced workload, new opportunities for collaboration, and improved process flows in the application area studied are described.		
	**Limits of use**	Perceived or anticipated limitations and challenges in using AI in a professional context. This includes technical, ethical, organizational, and work-related constraints that could limit the useful or desired use of AI.	**Technical limitations -** describes limitations due to susceptibility to errors, lack of flexibility, or a lack of understanding of complex issues.<br>**Ethical and legal concerns -** captures data protection issues, liability questions, or moral challenges.<br>**Organizational hurdles -** documents obstacles within the company structure, such as lack of acceptance or insufficient resources.<br>**Human skills -** addresses activities that respondents consider irreplaceable by AI, such as creative or social tasks**.**	
	**Understanding and previous experience**	Respondents' ideas and definitions of what AI means, as well as their previous interactions with and experiences with AI systems. This survey does not capture opinions or attitudes toward the technology, but rather knowledge and practical experience.		
	**Mindset**	Explicit and implicit cognitive, affective, and behavioral attitudes.<br>These include, in particular, attitudes, evaluations, feelings, and behaviors related to AI or technology as such.	**Opinion –** personal view or belief<br>**Mindeset –** basic inner attitude that shapes thinking and actions<br>**User behavior –** individual application of technical systems in everyday professional and private life	• Interest<br>• Aversion

**Discharge Planning**	**Process flow**	The structure, sequence, and logic of the individual steps within the process. This category captures how the process begins, which phases are passed through, and how the individual steps are linked.	**Steps and phases -** individual steps and their sequence<br>**Timelines -** consideration of the duration of individual steps and the overall process flow	• Starting point for process<br>• Process step<br>• Process duration<br>• Critical point
	**Stakeholders and responsibilities**	All individuals, groups, or organizations involved in the process. It records their respective roles, tasks, and responsibilities, as well as the manner in which they collaborate. It also examines how responsibilities are distributed and the interactions between the stakeholders.	**Roles involved -** Individuals, groups, or institutions involved in the process<br>**Responsibilities -** Description of the tasks and responsibilities of the respective stakeholders<br>**Interdisciplinary collaboration -** Cooperation between stakeholders with different professional backgrounds, competencies, and responsibilities within the process. This includes the nature of the collaboration, communication between disciplines and institutions, and challenges that arise. Potential conflicts, misunderstandings, or success factors for effective collaboration are also analyzed.	**Stakeholders**<br>Service Recipients<br>• Patients<br>• Relatives<br>Service Providers<br>• Health Insurance Funds (Statutory and Private)<br>• Long-Term Care Insurance Funds (Statutory and Private)<br>• Pension Insurance<br>• Assistance Offices<br>Service Providers<br>• Third-Party Funding Department<br>• Physicians<br>• Nurses<br>• Social Services<br>• Rehabilitation<br>• Senior Citizens Advice Centers<br>• Care Support Centers<br>• Medical Supply Stores<br>• Social Welfare Offices<br><br>Communication Channels/Types
	**Patient-centered care needs**	This category captures the specific medical, nursing, and therapeutic needs of patients. The focus is on the requirements for needs-based, safe, and continuous care. Critical issues that could jeopardize seamless care are also considered. Furthermore, the health literacy of patients is taken into account, as varying levels of knowledge about the healthcare system can influence their need for advice and support.	**Medical and nursing needs -** records the necessary diagnostic, therapeutic, nursing, and rehabilitative measures that patients require during their hospital stay.<br>**Health literacy and counseling needs -** extent of knowledge about the German healthcare system, existing care options, and the possibilities for accessing them. This also takes into account deficits in health literacy and the resulting need for information and counseling.	

	**Challenges and potential for improvement**	Explicitly and implicitly communicated needs and optimization opportunities within the process. This also includes key challenges, bottlenecks, and problem areas within the process. It identifies points where the process is inefficient, error-prone, or blocked.	**Resource requirements -** lack of financial, human, or technical resources to improve the process<br>**Structural challenges -** fundamental organizational, financial, and systemic problems that affect the functioning of hospitals and the German healthcare system<br>**Potential for improvement**<br>**Challenges and barriers -** problem areas that slow down or block the process	**Structural challenges**<br>• Staff shortages/skilled worker shortages<br>• Bureaucracy<br>• Economic challenges<br>• Insufficient care / gaps in care Digitalization and technical infrastructure<br>**Resource requirements**<br>• Time resources<br>• Technical resources<br>• Human resources
	**Information and Documentation**	Description of the collection, transfer, and storage of information within the process. Highlighting the data that is particularly relevant to the process. Furthermore, the completeness, comprehensibility, availability, and traceability of the information are considered.		• Collection of information<br>• Documentation<br>• Discharge-relevant information
