# Supplementary material for: Optimizing Hospital Discharge Planning: Empirical Insights and Requirements of AI-Based Technologies From an Explorative Mixed Methods Field Study
Source: JMIR Form Res. 2026 Mar 24;10:e81824. doi: 10.2196/81824 (PMC13012232; doi:10.2196/81824)
Supplement: Multimedia Appendix 7 [file formative-v10-e81824-s007.pdf]

Tabellarische Auswertung codierter Segmente

Inhalt

Workshop 1 1
Workshop 2 13
Workshop 3 25
Workshop 4 34
Workshop 5 39
Workshop 6 55

Workshop 1			
**Interview**	**Zeilen-Nr.**	**Generalisierung**	**Reduktion (auf Kategorie)**
STE-005	78-79	KI wirkt durch Algorithmus, ist lernfähig	Verständnis von KI
STE-005	80-82	KI hat festen Aufgabenbereich	Verständnis von KI
STE-005	83	Charakteristika von KI	Verständnis von KI
STE-005	89-91	KI als Hoffnungsträger	Hoffnung in KI
STE-005	93	KI als Chance für Arbeitserleichterung	Hoffnung in KI
STE-005	93	KI als Chance für Arbeitserleichterung	Bedürfnisse<br>Hoffnung
STE-005	95-97	Beratungsgespräche sehr ressourcenintensiv	Herausforderung
STE-005	97-98	KI gibt Pfad vor<br>Wunsch nach transparenten Zuständigkeiten	Bedürfnisse<br>Prozess
STE-005	101-105	Wunsch nach zentraler Dokumentation von Kundenkontakt/-kommunikation<br>Hohes Arbeitsaufkommen durch Kundenkontakte	Dokumentation<br>Wünsche/Bedürfnisse<br>Herausforderung
STE-005	106	Informationsfluss erschwert	Herausforderung
STE-005	109-111	Wunsch nach Automatisierung<br>KI soll Vorschläge machen	Wünsche/Bedürfnisse
STE-005	113-115	Wunsch nach Plausibilitätsprüfung<br>Nicht gangbare Wege sollen nicht auswählbar sein	Wünsche/Bedürfnisse
STE-005	115-116	Falsche Planung führt zu Mehrarbeit<br>Regelmäßiges Vorkommen solcher Ereignisse	Herausforderung<br>Bedürfnisse<br>Interdisziplinäre Zusammenarbeit
STE-005	121-122	EM wird bei SD angemeldet	Prozess<br>Interdisziplinäre Zusammenarbeit
STE-005	120-123	Anmeldung zur Nachversorgung durch med. Personal unstrukturiert + ineffizient	Herausforderung<br>Prozess<br>Interdisziplinäre Zusammenarbeit
STE-005	128-130	Bedarf und pers. Situation des Patienten werden nicht durch med. P. erhoben	Prozess<br>Herausforderung

			Interdisziplinäre Zusammenarbeit
STE-005	133	System soll zur Erledigung dazugehöriger Tätigkeiten verpflichten	Prozess<br>Wünsche/Bedürfnisse an KI<br>Interdisziplinäre Zusammenarbeit
STE-005	133-136	Med. P. zieht sich nach Anmeldung zum EM aus Prozess zurück	Prozess<br>Herausforderung<br>Interdisziplinäre Zusammenarbeit
STE-005	137-138	Standard wird nicht eingehalten	Herausforderung<br>Prozess<br>Interdisziplinäre Zusammenarbeit
STE-005	139	Anmeldungen zum EM bleiben oft aus	Prozess<br>Herausforderung<br>Interdisziplinäre Zusammenarbeit
STE-005	139-142	Anmeldungen zum EM erfolgen oft zu spät	Prozess<br>Herausforderung<br>Kritischer Punkt innerhalb des Prozesses<br>Interdisziplinäre Zusammenarbeit
STE-005	137	Komplexität des Versorgungsbedarfs korreliert mit Anspruch an EM	Prozess<br>Nachversorgung<br>Interdisziplinäre Zusammenarbeit
STE-005	156	Großes Arbeitsaufkommen durch (manuelle) Bearbeitung der Anrufe	Herausforderung<br>Prozess<br>Kommunikationskanäle<br>Beratung
STE-005	207	KI hat maschinellen Sprachstil	Erfahrung mit KI
STE-005	210	Erfahrung mit LLM	Erfahrung mit KI
STE-005	217	Erfahrung mit LLM	Kenntnis zu KI<br>Grenzen von KI
STE-005	219-221	Erfahrung mit LLM<br>Grenzen von KI	Kenntnis zu KI<br>Herausforderung
STE-005	225	Neugierig auf KI	Kenntnisse zu KI<br>Einstellung/Haltung

STE-005	223	Nutzung von KI im Alltag	Nutzung von KI
STE-005	230	Interesse an KI	Haltung/Einstellung zu KI<br>Kenntnis zu KI
STE-005	235-236	Chancen/Vorteile<br>Funktionsweise	Kenntnisse zu KI<br>Hoffnung<br>Bewusstsein der Einflussmöglichkeiten durch Anwender
STE-005	230-231	KI lernt durch Rückmeldung	Kenntnis zu KI
STE-005	240-241	Kritische Reflexion der Nutzung von KI	Kenntnis zu KI<br>Ethische Bedenken
STE-005	249	Teilnehmende war von ethischem Aspekt des Lernens der KI überrascht<br>Neue Erkenntnis über KI	Kenntnis zu KI<br>Ethische Bedenken
STE-005	263-265	Reflektion/Kritische Auseinandersetzung in Bezug auf Anwendung in klinischen Standorten<br>Sorge?<br>Interesse an Funktionsweise/Datengrundlage der KI (für die eigene Berufstätigkeit?)	Prozesse<br>Herausforderung<br>Interesse an KI
STE-005	270-272	Unterschiedliche Verantwortlichkeiten und Prozessabläufe	Prozesse
STE-008	11-12	Patient ist Kern des Prozesses<br>Prozessinhaber	Akteur<br>Prozess
STE-008	24-26	Beteiligte Akteure	Akteur<br>Prozess
STE-008	29-31	Zuständigkeiten werden gegenseitig verordnet	Herausforderung<br>Prozess
STE-008	32	Beteiligte Akteure	Akteur<br>Prozess
STE-008	37	Beteiligte Akteure	Akteur<br>Prozess
STE-008	40	Beteiligte Akteure	Akteur<br>Prozess
STE-008	51	Bedeutender Akteur	Akteur<br>Prozess
STE-008	52-53	Bedeutender Akteure	Akteur

			Prozess
STE-008	72-77	Beteiligte Akteur	Akteur<br>Prozess
STE-008	79-80	Beteiligte Akteure	Akteur<br>Prozess<br>Herausforderung
STE-008	80-84	Oberarzt verhindert Maßnahmen, wenn diese nicht ausreichend finanziert werden. Auch, wenn diese medizinisch sinnvoll sind<br>Genehmigung der Maßnahme durch Oberarzt scheinbar erst nach Gespräch zwischen SD und Pat./Angehörigen	Prozess<br>Herausforderung<br>Finanzierung
STE-008	85-88	Patient ist Auftraggeber<br>Prozesshoheit liegt bei Facharzt/Oberarzt	Prozesshierarchie<br>Kritischer Punkt innerhalb des Prozesses<br>Finanzierung
STE-008	90-92	Nachversorger sind sehr wichtig	Kritischer Punkt innerhalb des Prozesses<br>Kontinuität der Versorgung
STE-008	94-95	KK übernimmt Finanzierung	Akteur<br>Kritischer Punkt innerhalb des Prozesses<br>Kontinuität der Versorgung<br>Finanzierung
STE-008	97-98	Finanzierung von KK abhängig	Prozess<br>Kritischer Punkt<br>Finanzierung<br>Akteur
STE-008	100-101	Viele KK als beteiligte Akteure	Akteur<br>Prozess<br>Herausforderung
STE-008	101-106	KK ist bedeutender Akteur	Akteur<br>Prozesshierarchie<br>Kritischer Punkt innerhalb des Prozesses

STE-008	107-109	KK haben Weisungsbefugnis	Akteur<br>Prozesshierarchie
STE-008	114-115	RK haben Weisungsbefugnis	Akteur<br>Prozesshierarchie
STE-008	120-125	Oberarzt ordnet Entlassung an, wenn Patient zulange hospitiert ist und dadurch Kosten entstehen	Akteur<br>Prozess<br>Finanzierung<br>Zuständigkeit
STE-008	138-139	SD und PÜ haben unterschiedliche Prozessabläufe	Akteur<br>Prozess
STE-008	154-155	Es gibt eine Prozessdefinition	Standardisierung<br>Prozessablauf
STE-008	158	Prozessbeschreibung wird nicht regulär eingehalten	Standardisierung<br>Prozessablauf
STE-008	158-161	Es gib keinen Standard. Ablauf wird mündlich weitergegeben und muss gelernt werden	Standardisierung<br>Prozessablauf
STE-008	161-163	Vorgehen beim EM begründet sich durch Expertise, Erfahrung des TN	Prozessablauf<br>Entscheidungsfindung<br>Standardisierung
STE-008	167-173	Klinischer Auftrag soll bei Aufnahme, spätestens binnen 24h, erfasst und an SD weitergeleitet werden. Daran schließt Bedarfserhebung an	Prozessablauf<br>Klinischer Auftrag<br>Zuständigkeiten
STE-008	174-179	Es besteht ein pflegerische und ärztl. Standard. Für EM benötigter Input durch andere Berufsgruppen ist größtenteils nicht gegeben, sodass EM Prozessablauf ändern und fehlende Prozessschritte kompensieren muss	Interdisziplinäre Zusammenarbeit<br>Herausforderung<br>Prozess<br>Zuständigkeit
STE-008	178-186	Für das EM relevante Daten und Informationen werden nicht immer von Ärzten und Pflege erhoben oder zur Verfügung gestellt. Drittmittelabteilung führt öfter nicht Maßnahmen zur Aufnahme und Abrechnung durch	Interdisziplinäre Zusammenarbeit<br>Herausforderung<br>Prozess<br>Zuständigkeit

STE-008	190-92	Patient gilt als aufgenommen, wenn er versorgt werden möchte, allem zugestimmt hat und SD einen klinischen Auftrag erhält	Prozessbeginn<br>Klinischer Auftrag
STE-008	198-204	Klinischer Auftrag kann von Arzt, Pflege, Patient oder Bevollmächtigten ausgelöst werden. Ist erster Prozessschritt des EM	Prozessbeginn<br>Klinischer Auftrag
STE-008	210-211	SD soll gemäß Prozessdefinition und möchte ausführliche Beratung und psychosoziale Intervention durchführen. Kann dies aber nicht umsetzen, da Ressourcen für fachfremde Tätigkeiten gebraucht werden.<br>Zweiter Prozessschritt	Herausforderung<br>Interdisziplinäre Zusammenarbeit<br>Prozess<br>Zuständigkeit<br>Beratung<br>Dissonanz
STE-008	214	Patient muss im System angelegt und aufgeklärt sein	Prozessablauf<br>Beratung
STE-008	217-219	Stomatherapie und -beratung leisten pflegerische Betreuung	Akteur
STE-008	226-232	EM nimmt Kontakt zu Ärzten und Pflege auf um Entlasstag zu erfragen und Ersteinschätzung zu erhalten. Barthel-Index ist sehr wichtig	Prozessablauf<br>Prognose des Entlasstags<br>Kommunikation<br>Assessments<br>Interdisziplinäre Zusammenarbeit
STE-008	234-236	SD nimmt zuerst Kontakt zu Ärzten und Pflege auf, um Präsenz zu zeigen	Interdisziplinäre Zusammenarbeit<br>Prozessablauf<br>Kommunikation
STE-008	239	Kommunikationsmedien sind Telefon, persönlich, Mail und Fax	Kommunikationswege<br>Interdisziplinäre Zusammenarbeit
STE-008	241-242	Erstkontakt dient der Bedarfserhebung	Prozessablauf<br>Bedarfserhebung
STE-008	244	Handynummer des Pat. Ist Idealfall	Kommunikationsweg
STE-008	247-248	Nicht jeder Patient hat Wunsch nach EM	Bedarf<br>Prozessablauf
STE-008	250-251	Zuerst persönliche Kontaktaufnahme mit Pat. Oder Angehörigen	Kommunikation<br>Prozessablauf
STE-008	252-256	Patienten konnten früher mit Hol- oder Bringdienst zu SD gelangen. Aufgrund von Personalmangel geht das nicht mehr	Prozessablauf<br>Herausforderung<br>Kommunikationsweg

STE-008	257-260	Zu Beginn benötigt SD Information über kognitiven Status des Pat. Und etwaige Betreuer	Informationsbedarf des SD
STE-008	263	Patienten können Termine bei SD ausmachen	Kommunikationsweg<br>Prozess
STE-008	264	Patienten können Termin ausmachen.	Kommunikationsweg<br>Prozess<br>Partizipation des Patienten am EM-Prozess
STE-008	268	Patienten kommen unangekündigt bei SD vorbei	Herausforderung<br>Kommunikationsweg<br>Prozess<br>Partizipation des Patienten am EM-Prozess
STE-008	269-272	Ambulanz schickt Pat. Ohne Termin zu SD. Diese blockieren dann Ressourcen und lassen vergebene Termine platze	Kommunikationsweg<br>Prozess<br>Herausforderung<br>Interdisziplinäre Zusammenarbeit<br>Partizipation des Patienten am EM-Prozess
STE-008	274-277	Bei planbaren Eingriffen kontaktiert SD Patienten im Vorfeld und klärt Antragstellung ab	Kommunikationsweg<br>Prozessablauf<br>Partizipation
STE-008	279-280	Planung vorab bei akuten Erkrankungen nicht möglich	Kommunikationsweg<br>Prozessablauf
STE-008	281-285	Tatsächlicher Hilfebedarf einer Person fällt insbesondere bei Patienten für Geriatrie erst im Verlauf auf	Bedarf<br>Prozessablauf<br>Herausforderung
STE-008	286-290	Viele Menschen setzen sich nicht mit der Thematik „Vorsorge“ auseinander. Ein/e langjährige/r Partner/in wird nicht automatisch als Betreuer/in anerkannt	Vorsorge<br>Betreuung<br>Partizipation/Selbstbestimmung<br>Herausforderung
STE-008	294-295	Ältere Personen befassen sich oft nicht mit dem Tod und Sterben	Vorsorge

			Betreuung<br>Partizipation/Selbstbestimmung<br>Herausforderung
STE-008	303	MA des SD hat mehrere Stationen zu betreuen	Prozess<br>Zuständigkeit
STE-008	306	SD-MA haben feste Regelung der Zuständigkeit	Prozess<br>Zuständigkeit
STE-008	308-310	MA kennen zuständige SD und sind personenfixiert	Zuständigkeit<br>Interdisziplinäre Zusammenarbeit
STE-008	317-319	Verweildauer ist abhängig von Erkrankung	Prozess<br>Verweildauer<br>Bedarf
STE-008	320-322	Verweildauer mancher Patienten teilweise Jahre. Kommen ein bis zwei mal pro Monat. Bei ihnen wir Zeit abgekappt.	Prozess<br>Verweildauer<br>Bedarf<br>Beratung<br>Herausforderung
STE-008	323-327	Patienten haben unterschiedliche Bedarfe an Beratung	Beratung<br>Versorgung
STE-008	329-331	Barthel-Index wird meist nicht ohne Aufforderung erhoben, obwohl er Pflicht ist	Zuständigkeit<br>Interdisziplinäre Zusammenarbeit<br>Herausforderung<br>Prozess<br>Bedarf<br>Indikator Nachsorgebedarf
STE-008	332-335t	Barthel-Index wird benötigt, um Verlauf der Krankheit/Genesung zu ermitteln. Dient auch als Argument für Kostenübernahme durch KK	Finanzierung<br>Bedarf<br>Versorgung<br>Indikator Nachsorgebedarf

STE-008	342-343	Barthel-Index ist pfleg. Aufgabe. Index kann abgerechnet werden	Zuständigkeit<br>Finanzierung<br>Interd. Zusammenarbeit
STE-008	345-346	Einschätzung des Patienten bereits früh möglich	Bedarf<br>Prozess<br>Versorgung
STE-008	347-349	Pfleg. Einschätzung als Assessment nicht ausreichend für eine vollständige Darstellung des Zustands	Bedarf<br>Versorgung<br>Indikator Nachsorgebedarf<br>Assessment<br>Interd. Zusammenarbeit
STE-008	350-351	Pfleg. Assessment ist oft nicht hinterlegt	Bedarf<br>Versorgung<br>Assessment<br>Interd. Zusammenarbeit<br>Herausforderung<br>Dokumentation
STE-008	362-354	Aktualisierung der Assessment durch Pflege erfolgt nicht, ist Problem für Einschätzung durch SD	Bedarf<br>Versorgung<br>Assessment<br>Interd. Zusammenarbeit<br>Herausforderung<br>Dokumentation
STE-008	355-359	SD muss sich selbst vor Ort ein Bild des Patienten machen	Bedarf<br>Versorgung<br>Assessment<br>Interd. Zusammenarbeit<br>Herausforderung<br>Dokumentation
STE-008	369-371	Dokumentation der Pflege oft unvollständig. PK haben keine Zeit oder kennen Pat. Noch nicht	Bedarf<br>Versorgung<br>Assessment<br>Interd. Zusammenarbeit

			Herausforderung<br>Dokumentation
STE-008	372	?	
STE-008	376-377	SD wünscht sich, dass Prüfung auf Vollständigkeit der Assessments automatisch geschieht	Hoffnung in Bezug auf KI<br>Wünsche/Bedürfnisse<br>Dokumentation<br>Kontrolle
STE-008	377-382	SD übernimmt Aufgabe der Pflege, die nicht abgerechnet werden können, damit Entlassung gelingt.	Bedarf<br>Versorgung<br>Prozess<br>Kritischer Punkt innerhalb Prozess<br>Assessment<br>Interd. Zusammenarbeit<br>Herausforderung<br>Dokumentation<br>Finanzierung
STE-008	384-385	Durch fehlende Tel.-Nr. wird EM behindert	Prozess<br>Bedarf<br>Kommunikationsweg<br>Herausforderung<br>Dokumentation
STE-008	387-388	Mit Tel.-Nr. können Infos schnell eingeholt werden	Prozess<br>Kommunikationsweg<br>Bedarf<br>Herausforderung<br>Dokumentation
STE-008	389-395	SD muss Zeit aufwenden, um Daten zu erhalten, die eigentlich durch Aufnahme erhoben werden sollte. Andere Instanzen benötigen diese Daten auch. PK fühlen sich belästigt von Informationssuche des SD	Prozess<br>Zuständigkeiten<br>Herausforderung<br>Dokumentation
STE-008	396-400	Ist Pflegebedarf erhoben, wird persönlich mit Pat./Angehörigen gesprochen und Setting erfragt	Bedarf<br>Prozessablauf

STE-008	403-405	Wenn Pflegedienst als Nachsorger eintritt, müssen benötigte Hilfsmittel ermittelt und beschafft werden	Nachsorge<br>Versorgung<br>Bedarf<br>Hilfsmittel<br>Interd. Zusammenarbeit<br>Kontinuität<br>Kritischer Punkt
STE-008	406-421	Teilweise sind häusl./persönl. Situationen der Patienten herausfordernd, sodass ethische Bedenken seitens der Pflege bestehen, ob dieser Pat. Entlassen werden kann. Aus rechtl. Aspekt steht der Entlassung nichts im Wege. Spannungsfeld für SD	Herausforderung<br>Interd. Zusammenarbeit<br>Versorgung<br>Ethische Bedenken<br>Kontinuität
STE-008	428-430		Herausforderung<br>Ethische Bedenken<br>Interd. Zusammenarbeit<br>Versorgung<br>Kontinuität
STE-008	435-436	Pflegegrad soll bei Aufnahme hnterlegt werden	Prozessablauf<br>Information/Daten
STE-008	437-439	Pflegegrad muss nicht angegeben werden, nur dass einer vorhanden ist	Inforation/Daten<br>Interd. Zusammenarbeit
STE-008	440	Dokumentation wird meist nicht gemacht	Herausforderung<br>Prozess<br>Bedarf<br>Interd. Zusammenarbeit<br>Versorgung
STE-008	443-445	Pflegegrad wird nicht dokumentiert, obwohl oft angesprochen. Sollte spätestens bei Aufnahme auf Station dokumentiert sein	Herausforderung<br>Prozess<br>Bedarf<br>Interd. Zusammenarbeit<br>Versorgung

STE-008	452-453	Pat. Wird für Nachsorge angemeldet, die nicht gebraucht wird, weil sie schon besteht	Herausforderung<br>Prozess<br>Dokumentation<br>Bedarf<br>Interd. Zusammenarbeit<br>Versorgung
STE-008	462-465	Pat. Sieht keinen Beratungsbedarf, wenn von ärztl. Seite kommuniziert wird, das der Eingriff erfolgreich war	Herausforderung<br>Prozess<br>Bedarf<br>Interd. Zusammenarbeit<br>Versorgung
STE-008	466-471	Negative Vorerfahrung der SD-MA, weil Pat. Nicht von Arzt erfuhr, dass er Palliativstatus hat.	Herausforderung<br>Prozess<br>Zuständigkeit<br>Bedarf<br>Interd. Zusammenarbeit<br>Versorgung
STE-008	472-474	SD erfährt nicht automatisch von Station, dass Pat. Verstorben ist. Bekommt Info tlw. Von Angehörigen	Wunsch/Bedfürniss an KI<br>Herausforderung<br>Interd. Zusammenarbeit<br>Kommunikation<br>Dokumentation
STE-008	477-480	Ärzte informieren SD nicht, wenn Pat. Verstirbt oder verlegt wird	Wunsch/Bedürniss an KI<br>Herausforderung<br>Interd. Zusammenarbeit<br>Kommunikation<br>Dokumentation
STE-008	482-485	Falsche Angaben/Lückenfüller in Maske erschweren Arbeit des SD	Wunsch/Bedürniss an KI<br>Herausforderung<br>Interd. Zusammenarbeit<br>Kommunikation<br>Dokumentation

Workshop 2			
**Interview**	**Zeilen-Nr.**	**Generalisierung**	**Reduktion (auf Kategorie)**
STE-010	67	Feste Zuständigkeiten der Sozialarbeiter	Prozess<br>Zuständigkeiten
STE-010	86-91	TN hat Vorerfahrung mit ChatGPT. Nutzt es privat und beruflich. Wendet es als Unterstützung zur Formulierung von Botschaften. Ist sehr begeistert davon	Einstellung zu KI (Unterstützung)<br>Chancen<br>Vorkenntnis<br>Nutzverhalten<br>Beratung
STE-010	93	Nutzt ChatGPT als Inspiration	
STE-010	98	Grenzen der Pflegerobotik sind TN bekannt	Vorkenntnis<br>Grenzen
STE-010	104	TN ist interessiert an KI	Einstellung zu KI (Interesse)
STE-010	124-125	Hofft bzw. wünscht, dass KI dafür sorgt, dass Eingabefelder vollständig und korrekt ausgefüllt werden	Chancen<br>Hoffnungen<br>Wünsche<br>Prozess (KLAU)<br>Bedarf
STE-010	126-128	Relevante Eingabefelder innerhalb der KLAU werden regelm. Mit Platzhaltern gefüllt. SD muss dann Informationen zur Kontaktaufnahme selber ausfindig machen. Kontaktieren sie dazu die Station, sind die MA dort genervt	Interd. Zusammenarbeit<br>Herausforderung<br>Prozesse (KLAU)<br>Kommunikation<br>Dokumentation<br>Information/Daten<br>Bedarf
STE-010	131-132	TN möchte wissen, welche Funktionen KI in Bezug auf KLAUs hat	Interesse<br>Chancen
STE-010	146-147	Unklarheit/Unstimmigkeit darüber, was das bestehende System kann	Dokumentation<br>Information/Daten
STE-010	158	Anamnese und Barthel sind Instrumente auf Station	Dokumentation

			Prozesse
STE-010	160-161	TN wünscht, dass KI selbständig Daten einfügt oder Station darin erinnert, es zu tun<br>Wenn Barthel ausgefüllt ist, spart sich SD Ressourcen zur Informationsbeschaffung	Chancen<br>Hoffnungen<br>Wünsche<br>Prozess (KLAU)<br>Bedarf<br>Dokumentation<br>Interd. Zusammenarbeit
STE-010	162-164	Wenn SD Einsicht in Informationen der Derma hätte, würde er sich viele Telefonate sparen	Chancen<br>Hoffnungen<br>Wünsche<br>Bedarf<br>Dokumentation<br>Interd. Zusammenarbeit
STE-010	177-179	Wunsch nach automatischer Dokumentation und Informationsübertragung	Chancen<br>Hoffnungen<br>Wünsche<br>Bedarf<br>Dokumentation<br>Standardisierung
STE-010	182	Wunsch an KI, dass Textbausteine vorgelegt sind	Chancen<br>Hoffnungen<br>Wünsche<br>Bedarf<br>Dokumentation
STE-010	189-193	KI hat Predigten geschrieben, die waren wenig emotional	Grenzen KI (Emotionalität)
STE-010	195-197	KI soll Dinge ersetzen. Berufsgruppen?	Einstellung KI (Sorge)<br>Ethische Bedenken
STE-010	211	Hofft auf mehr Zeit für Patienn	Chancen<br>Hoffnungen<br>Wünsche
STE-010	216-219	Interesse an Funktionsweise, Sorge	Einstellung zu KI (Interesse, Sorge)

STE-010	239-241	Sorge, dass freie Zeitkontingente nicht dem Patientenkontakt gewidmet wird, sondern durch AG Personal reduziert wird	Sorge KI
STE-010	342	Teilt Sorge	Sorge KI
STE-010	277-278	Hoffnung auf mehr Zeit für Patienten und Angehörige	Chancen<br>Hoffnungen<br>Wünsche
STE-010	280	Hoffnung auf schnelle Erfassung der Dokumentation	Chancen<br>Hoffnungen<br>Wünsche<br>Dokumentation
STE-010	283-285	Wunsch, dass KI automatisch KLAU anlegen kann	Chancen<br>Hoffnungen<br>Dokumentation<br>Information/Daten<br>Wünsche<br>KLAU
STE-010	287	Bedenken in Bezug des Aspekts Datenschutz	Sorgen<br>Bedenken
STE-010	305	Möchte wissen, wo Daten gespeichert sind	Interesse an KI<br>Dateschutz
STE-010	315	Schutz vor Hackern	Sorgen<br>Bedenken<br>Datenschutz
STE-010	343-344	Sorge um Patientengefährdung	Sorgen<br>Bedenken<br>Datenschutz
STE-010	368	Wünscht Bürokratieabbau	Chancen<br>Hoffnungen<br>Wünsche<br>Dokumentation<br>Herausforderung

STE-010	381-382	Interessiert an Funktionsweise	Einstellung (Interesse)
STE-010	393	Findet Lernfähigkeit der KI gruselig	Einstellung (Sorge)<br>Bedenken<br>Vorerkenntnisse
STE-010	404-410	Sorge, ob automatisierte Anrufweiterleitung emotional aufgeladen Pat. Gerecht werden kann	Einstellung (Sorge)<br>Bedenken<br>Ethische Bedenken<br>Bedarf<br>Beratung
STE-010	420-421	Immer wieder gestellte Fragen hemmen Arbeitsfortschritt des SD	Herausforderung<br>Beratung
STE-010	433	Manche Personen finden den Kontakt des SD nicht in Webpräsens	Herausforderung<br>Kommunikation/Kontakt<br>Bedarf<br>Versorgung
STE-010	442	Abhören des Abs tlw. Herausfordernd	Herausforderung<br>Kommunikationsweg<br>Beratung
STE-010	446-448	Wunsch, dass Sprachnachricht von Pat. Auf AB als Textnachricht im E-Mail-Postfach ankommt	Wunsch<br>Chance<br>Bedarf<br>Kommunikationsweg
STE-010	452-457	Schilderung einer sehr emotionalen Frau, die wegen ihrer Aufregung die Fragen des SD nur schwer beantworten konnte. Vermutung, dass diese Angehörige durch Assistenzsystem per Telefon nicht abgeholt werden könne	Grenzen<br>Sorge/Bedenken<br>Herausforderung (emotional aufgewühlte Personen)<br>Kommunikationsweg<br>Information (herausfordernd für Beschaffung)
STE-010	482-491	TN differenziert zwischen verschiedenen Arten von Tätigkeiten, die ihrer Meinung nach für den Einsatz von KI geeignet sind	Chancen und Grenzen<br>Risiken und Gefährdungen

			Wünsche<br>Prozesstätigkeiten (die für Automatisierung geeignet sind)<br>Ethische Bedenken
STE-010	502-511	Sorge, dass höhere Arbeitsbelastung des SD durch Automatisierung eintreten könnte	Sorge/Bedenken<br>Ethische Bedenken<br>Gefahr<br>Prozess<br>Selbstkundgabe (sieht sich als alt)
STE-010	515-519	Sorge, dass Stellen aufgrund der KI nicht (nach-)besetzt werden	Sorge/Bedenken<br>Ethische Bedenken<br>Gefahr<br>Prozess
STE-010	524-531	Komplexität und Anspruch an die Versorgung der Pat. Steigt seit Jahren. Diese werden im durchschnitt immer älter und morbider. Angehörige als Ressource (für Pat. + SD) entfällt zunehmend. Daher höhere Arbeitsaufwand pro Patient	Versorgung<br>Bedarf<br>Ressourcen der Patienten<br>Herausforderung<br>Partizipation
STE-010	537-538	Ki als unvermeidbares Phänomen, dass jeden einholt	Einstellung/Wahrnehmung
STE-010	544-545	KI kann emotionale Bedürfnisse nicht befriedigen	Grenzen<br>Einstellung<br>Versorgung
STE-010	563-565	Empfindet hohem Papierverbrauch als unangehnehm	Einstellung<br>Herausforderung<br>Ressourcen
STE-010	568-570	Sieht grundlegenden Klärungsbedarf	Einstellung<br>Standardisierung<br>Kommunikationsweg
STE-010	589-591	Sinnvoll wäre aus ihrer Sicht, dass Akteurübergreifend digital gearbtet wird	Interd. Zusammenarbeit<br>Grenzen<br>Einstellung<br>Kommuniktionsweg

			Dokumentation<br>Information
STE-010	607-610	Unterschiedliche Arbeitsweise/Nutzung verschiedene Kommunikationskanäle durch MA	Interd. Zusammenarbeit<br>Grenzen<br>Einstellung<br>Kommuniktionsweg<br>Dokumentation<br>Information
STE-010	644-645	Alle Berufsgruppen haben Zeitprobleme und Personalmangel	Herausforderung<br>Interd. Zusammenarbeit
STE-010	646-652	Klinisches Personal sieht aus Sicht der TN den Prozess als Ganzes nicht. Es wäre keine Sensibilität dafür da, dass die Daten für die Entlassung das Patienten benötigt würden, sodass die Betten frei gemacht werden können. Diese Aufgabe wird ausschließlich bei SD/PÜ verortet	Herausforderung<br>Interd. Zusammenarbeit<br>Information<br>Daten<br>Dokumentation
STE-010	655-656	Prozess der Pflegeüberleitung startet mit Anamnese, danach darf erst zur PÜ oder EM angemeldet werden	Prozess<br>Prozessbeginn<br>Standardisierung<br>Interd. Zusammenarbeit
STE-010	660-662	Anamnesebögen schrecken ab, weil sie so groß sind	Herausforderung<br>Dokumentation<br>Information/Daten<br>Interd. Zusammenarbei
STE-010	661-666	Wunsch nach mobilen Endgeräten zur Dokumentation	Wunsch<br>Bedarf<br>Dokumentation<br>Daten
STE-010	789-793	Einstellung zu Neuem ist, dass am Anfang alles herausfordernd ist. Wenn man sich mit dem Gegenstand aber auseinandersetzt, kann man lernen	Einstellung<br>Selbstkundgabe (Bereitschaft)<br>Partizipation
STE-012	11	Siebter Schritt ist Kostenzusage	Prozessschritt
STE-012	12	Schritt danach ist Entlassungstag	Prozessschritt

STE-012	13-14	Unterschiedliche Vorgehensweisen der Professionen, bei Sozialdienst kann es sein, dass nach Entlassung Anträge und Beratung stattfinden.<br>Bei Pflegeüberleitung muss dies vor der Entlassung geschehen	Zuständigkeit<br>Prozess<br>Beratung
STE-012	16-20	Bei Pflegeüberleitung müssen Verträge vor Entlassung geschlossen sein	Finanzierung<br>Nachsorge<br>Prozess<br>Kritischer Punkt<br>Kontinuität
STE-012	22-23	Keine Aufnahme zur KZP ohne Vertrag	Finanzierung<br>Nachsorge<br>Prozess<br>Kritischer Punkt<br>Kontinuität
STE-012	27-28	Aufnahme in den Pflegedienst und Beschaffung der Hilfsmittel muss gewährleistet sein, bevor Pat. Entlassen werden kann	Finanzierung<br>Nachsorge<br>Prozess<br>Kritischer Punkt<br>Kontinuität
STE-012	42-47	Bei onkologischen Patienten in der Chirurgie findet die Besprechung erst nach der Entlassung statt.	Beratung<br>Prozess
STE-012	54	Es gibt Fristen, die eingehalten werden müssen	Prozess
STE-012	58-59	Bei Sozialdienst kann alles auch nach der Entlassung stattfinden	Beratung<br>Prozess
STE-012	85-86	Herausforderungen beim KLAU sind, dass Patientendaten nicht vollständig oder inkorrekt sind oder dass unsinnige bzw. unklare Aufträge erteilt werden	Herausforderung<br>Dokumentation<br>Prozess (wird behindert)<br>Information<br>Interd. Zusammenarbeit<br>KLAU
STE-012	91-94	Vorschlag zu Herausforderungen mit KLAU ist, dass ein Auftrag erst freigegeben wird, wenn alle erforderlichen Daten vorliegen und eine objektiv nachvollziehbare Notwendigkeit des EM gegeben ist	Chance<br>Bedarf

			KLAU
STE-012	94-95	KI soll zur Anlegung eines KLAU auffordern, wenn gewisse Kriterien bei Patient vorliegen	Chance<br>Bedarf<br>KLAU
STE-012	97-98	Es kommt oft vor, dass Angehörige oder Ärzte das EM einschalten wollen, aber kein KLAU angelegt ist	Interd. Zusammenarbeut<br>KLAU<br>Herausforderung
STE-012	113-115	Ohne KLAU kann SD nicht aktiv werden	KLAU<br>Herausforderung
STE-012	121-129	Mehrfachanmeldungen für den KLAU sind nervig. Sie entstehen, weil SD Befunde des Arztes braucht, um Anmeldung bei Nachversorger zu realisieren. Ärzte lesen aber die entsprechende Dokumentation des Pat. Nicht. Er oder die Pflege denken, dass der SD den EM nicht nachkommt und eröffnen einen neuen Auftrag.	Interd. Zusammenarbeut<br>KLAU<br>Herausforderung
STE-012	131-132	Dokumentation wird oft nicht gelesen	Interd. Zusammenarbeut<br>KLAU<br>Herausforderung<br>Dokumentation
STE-012	135-138	Mehrfachanmeldung zum EM infolge von Verlegung des Patienten	KLAU<br>Herausforderung<br>Dokumentation
STE-012	191-197	Dokumentation im System ist teilweise sehr undurchsichtig. Einträge des SD werden teilweise durch MA unter älteren Einträgen fortgeführt. Andere legen neue Dokumente für aktuelles EM an. Besonders bei Patienten, die mehrere Aufenthalte habe, kann das zu Verwirrung führen	Herausforderung<br>Dokumentation<br>Interd. Zusammenarbeit<br>Daten<br>Information
STE-012	229-238	Bei Anliegen in Zusammenhang mit IT geben die zuständigen Kollegen keine zuverlässige Auskunft bzw. Hilfestellung. Man muss wissen, welcher Ansprechpartner Kompetenzen im jeweiligen Bereich hat.	Herausforderung<br>Interd. Zusammenarbeit<br>Zuständigkeit
STE-012	257-262	KI kann dabei unterstützen, den Barthel-Index zwingend einzutragen	Chance<br>Assessments<br>Interd. Zusammenarbeit
STE-012	266-267	Idealerweiße muss der Barthel-Index eingetragen sein, damit der KLAU anzulegen ist	Chance<br>Assessments

			Interd. Zusammenarbeit<br>KLAU
STE-012	317-324	Sinnvoll wäre eine automatische Meldung durch KI, wenn sich durch einen neuen Eintrag der Barthel-Index verändert und es in dessen Folge zu anderem Nachsorgebedarf kommt.	Chance<br>Nachsorge<br>Bedarf
STE-012	362-363	Benachrichtungswunsch, wenn ein Pat. Einer OP unterzogen wird	Chance<br>Wunsch<br>Bedarf<br>Interd. Zusammenarbeit<br><br>Dokumentation
STE-012	373-374	Wünschenswert sind persönliche Einstellungen zum Erhalt von Benachrichtungen	Chance<br>Wunsch
STE-012	447-451	Chatbot für Ermittlung der zuständigen Kostenträger sind wünschenswert für komplexe Fälle	LLM<br>Finanzierung<br>Wunsch<br>Information
STE-012	506-519	Herausfordernd sind Patienten ohne klaren Versicherungsschutz, Obdachlose ohne festen Wohnsitz, Geflüchtete und Ausländer. Hier besteht oft Unklarheit bzgl. Kostenübernahme	Herausforderung<br>LLM<br>Finanzierung<br>Wunsch<br>Information<br>Akteur - Patient
STE-012	644-645	Alle haben Zeitprobleme und Personalmangel	Herausforderung
STE-012	646-652	Klinisches Personal sieht aus Sicht der TN den Prozess als ganzes nicht. Es wäre keine Sensibilität dafür da, dass die Daten für die Entlassung das Patienten benötigt würden, sodass die Betten frei gemacht werden können. Diese Aufgabe wird ausschließlich bei SD/PÜ verortet	Herausforderung<br>Interd. Zusammenarbeit<br>Dokumentation<br>Informationen
STE-012	655-656	Prozess der Pflegeüberleitung startet mit Anamnese, danach darf erst zur PÜ oder EM angemeldet werden	Prozessbeginn
STE-012	660-661	Anamnesebögen schrecken ab, weil sie so groß sind	Herausforderung<br>Dokumentation<br>Interd. Zusammenarbeit

STE-012	661-665	Wunsch nach mobilen Endgeräten zur Dokumentation	Chance
STE-012	789-793	Einstellung zu Neuem ist, dass am Anfang alles herausfordernd ist. Wenn man sich mit dem Gegenstand aber auseinandersetzt, kann man lernen	Einstellung
STE-013	1	Prozessschritt 5 ist Übermittlung an Kostenträger	Prozesschritt
STE-013	4-8	Ärzte füllen notwendige Unterlagen spät, falsch oder gar nicht aus.	Herausforderung<br>Interd. Zusammenarbeit<br>Dokumentation
STE-013	9-10	Ärzte behaupten manchmal, dass Nachrichten oder Dokumente sie nicht erreicht haben oder ihre Nachricht abgesandt wurde, obwohl sie nie beim SD ankam.	Herausforderung<br>Interd. Zusammenarbeit<br>Dokumentation<br>Kommunikation
STE-013	27	Bedarf liegt bei zentralem Dokumentenmanagementsystem	Bedarf<br>Dokumentation<br>Daten
STE-013	58	Herausforderung liegt im vorhandenen SAP. TN sind mit den Funktionen des bestehenden Systems nicht zufrieden	Bedarf<br>Herausforderung
STE-013	81-82	Manuelles Ausfüllen von Anträgen und Dokumenten benötigt 30% der Arbeitszeit	Herausforderung<br>Dokumentation<br>Daten
STE-013	83-88	Es gibt nur einen Bildschirm am Arbeitsplatz, das behindert die Tätigkeit, da es viele Daten zum Kopieen und Einfügen gibt.. Eine TN hat sich einen Bildschirm von zuhause mitgebracht.	Herausforderung<br>Dokumentation<br>Daten<br>Bedarf an techn. Ausstattung
STE-013	103-104	Anträge an die Kostenträger sind nicht im SAP hinterlegt	Dokumentation<br>System
STE-013	131-142	Rezepte werden oft falsch ausgestellt. Ärzte wissen oft nicht, dass nur ein Hilfsmittel pro Rezept von Sanitätshäusern akzeptiert wird. Eine KI könnte hier auf diesen Fehler im Erstellungsprozess hinweisen	Interd. Zusammenarbeit<br>Bedarf<br>Informationsdefizit (Ärzte)<br>Chance<br>Herausforderung
STE-013	235-236	KI soll Kostenträger automatisch ermitteln können	Wunsch

			Chance<br>Kostenträger<br>Zuständigkeit
STE-013	238	Prozessschritt 6 ist Information von Patienten und Station	Prozessschritt
STE-013	249-250	Information von Pat. Und Station erfolgt per Mail oder Telefon	Prozess<br>Kommunikationskanal<br>Interd. Zusammenarbeit
STE-013	256-258	Hilfreich in diesem Prozessschritt wäre, wenn KI Patienten per Mail automatisch über Termin zur Anschlussheilbehandlung informiert	Chance<br>Standardisierung<br>Kommunikation
STE-013	296-299	Wenn in der Cirurgie keine Tagesvisite durch SD gemacht wird, werden Patienten (durch die Ärzte?) gar nicht angemeldet. SD muss proaktiv auf Ärzte zugehen, damit Pat. Nachsorge vermittelt bekommt	Herausforderung<br>Bedarf<br>Interd. Zusammenarbeit<br>Nachsorge<br>Zuständigkeit<br>KLAU
STE-013	319-323	Prozesse im Haus wurden angepasst, weil Assessments nicht zuverlässig durchgeführt werden und Kommunikation zum SD durch med. P. nicht autonom vorgenommen wird. Folge ist, dass SD nun regelmäßigen Termin mit Ärzten hat, um alle Patienten hinsichtlich eines Nachsorgebedarfes zu besprechen.	Prozess<br>Zuständigkeit<br>Assessments<br>KLAU<br>Interd. Zusammenarbeit<br>Bedarf
STE-013	330-331	Bedarf des EM/der Nachsorge muss auf Grundlage der Anamnese angezeigt werden. In diesem Kontext muss die SD-MA in den persönlichen Dialog mit den Ärzten gehen und jeden Pat. der Abteilung mit ihm besprechen, um einen Bedarf zu ermitteln und aktiv werden zu können.	Prozess<br>Zuständigkeit<br>Assessments<br>KLAU<br>Interd. Zusammenarbeit<br>Bedarf
STE-013	362-368	Unterschiedliche Arbeitsweisen und Prozessabläufe zwischen den Abteilungen. Teilweise sehr große Unterschiede	Prozesse<br>Interd. Zusammenarbeit
STE-013	442	Prozessschritt 7 ist Kostenzusage	Prozessschritt
STE-013	446-449	Wenn Patient vor Rrehaantritt noch keine Kostenzusage hat, sollte Benachrichtigung durch Anwendung erfolgen	Chance<br>Bedarf

			Nachsorge
STE-013	459-466	Bei manchen KK erfährt man garnicht, ob die Kostenzusage erfolgt, andere müssen erneut telefonisch kontaktiert werden	Herausforderung<br>Kommunikation<br>Akteur – KK<br>Finanzierung
STE-013	469	TN legt sich eine Wiedervoralge an, wenn sie Herausforderungen innerhalb des Prozesses vermutet	Herausforderung<br>Kommunikation<br>Akteur – KK<br>Finanzierung
STE-013	470-471	Wenn Kostenzusage nicht da ist, wird entweder Reha-Termin verschoben oder Reha ruft nochmal an	Prozess<br>Finanzierung<br>Herausforderung<br>Kommunikation<br>Nachsorge<br>Kritischer Punkt (AHB wird verschoben)
STE-013	477-586	Das Lebensalter der Patienten ist ein Einflussfaktor auf die Nachversorgung. Personen im mittleren Alter (55 – 65) ohne unterstützendes soziales/häusliches Umfeld haben bei Bedarf der Versorgung nach Klinikaufenthalt Schwierigkeiten, einen Platz zu bekommen, da Pflegeheime eine KZP meist erst ab dem 65. Lebensjahr akzeptieren.	Herausforderung<br>Nachsorge<br>Bedarf<br>Partizipation<br>Akteur – Angehörige<br>Versorgung
STE-013	489-497	Patienten, die nicht rehafähig sind, zuhause aber keine Unterstützung erwarten können, können schwer entlassen werden	Herausforderung<br>Nachsorge<br>Bedarf<br>Partizipation<br>Akteur – Angehörige<br>Versorgung<br>Kritischer Punkt
STE-013	576-577	Hilfreich wäre, wenn Anwendung bei Personen unter 65 Jahren einen Hinweis gibt, dass diese nicht in eine Pflegeeinrichtung können	Chance
STE-013	613-616	Nachsorge zuhause gelingt eher, wenn Angehörige mithelfen.	Prozess<br>Akteur

			Nachsorge<br>Versorgung
STE-013	641-647	Vor Entlassung wird dem Patienten oder dessen Angehörigen eine Mail mit Information zum Entlassungsprozess gesendet. Damit werden sie auf wichtige Aspekte zur Vorbereitung hingewiesen	Prozess<br>Akteur<br>Nachsorge<br>Versorgung<br>Information<br>Beratung<br>Kommunikation
STE-013	648-653	Ideal wäre, wenn KI zum Entlassungszeitpunkt alle Befunde, Rezepte und Verordnungen an die jeweilig beteiligten Akteure aussendet	Prozess<br>Chance<br>Nachsorge<br>Versorgung<br>Information<br>Beratung<br>Kommunikation

Workshop 3

Interview	Zeilen-Nr.	Generalisierung	Reduktion (auf Kategorie)
STE-014	7-9	Bei OP-Verschiebung erfolgt oft keine Benachrichtigung des EM. Nachsorge kann dann nicht angepasst werden.	Herausforderung<br>Kommunikation<br>Daten/Information
STE-014	9-12	Wartezeit für orthopädische Rehas liegen bei 4-8 Wochen. In Geriatrie kann erst Versorgung übernommen werden, wenn Patienten vor Ort sind. Die meisten Kliniken arbeiten nicht mit Voranmeldungen.	Prozess<br>Nachsorge<br>Versorgung<br>Akteure – Nachversorger
STE-014	30-33	Sinnvoll wäre eine automatische Benachrichtigung bei Verschiebung oder Ausfall der OP	Chance<br>Kommunikation
STE-014	79-80	TN finden partizipativen Gestaltungsprozess gut	Partizipation
STE-014	99-100	KI ist künstliche Intelligenz, die Arbeitsalltag erleichtert	Chance
STE-014	105-106	KI kann nach Priorität selekieren	Chance

STE-014	119-124	Verschlechtert sich der Allgemeinzustand eines Patienten oder hat er noch keinen PG, wird einer von der Pflegeüberleitung beantragt	Prozessbeschreibung<br>Zuständigkeit
STE-014	127-132	Weder Ärzte noch Pflege machen Anamnese, obwohl diese Standard ist. TN muss dann selbst alle Informationen erheben.	Herausforderung<br>Interd. Zusammenarbeit<br>Standard<br>Information
STE-014	155-158	Dokumentation wird von klinischem Personal nicht gelesen.	Herausforderung<br>Interd. Zusammenarbeit<br>Standard<br>Information
STE-014	164-166	Patient muss befragt werden, ob er ins EM einwilligt	Prozessbeginn<br>Akteur – Patient<br>Beratung<br>Partizipation
STE-014	171-174	Klinisches Personal gibt regelmäßig an, dass Pat. nicht befragt werden kann, obwohl er adäquat ist.	Interd. Zusammenarbeit<br>Herausforderung<br>Dokumentation
STE-014	177-179	Einwilligung in EM muss von Pat. unterschrieben sein	Prozessbeginn<br>Akteur – Patient<br>Beratung<br>Partizipation
STE-014	199-200	Patienten werden wahllos zu allen möglichen Nachsorgemöglichkeiten angemeldet. Ein konkreter Bedarf wird nicht ermittelt, das wird vom EM verlangt	Herausforderung<br>Interd. Zusammenarbeit<br>Standard<br>Information<br>Nachsorge<br>Dokumentation
STE-014	217-222	KI kann alle entlassrelevanten Informationen an einem Ort übersichtlich bewahren.	Chance<br>Dokumentation
STE-014	235-239	Wunsch ist, dass KI eigenständig freie Kapazitäten von Nachsorgern ermittelt und anzeigt.	Chance<br>Nachsorger<br>Kommunikation (zw. Leistungserbringern)

STE-014	240-241	Es müssen dutzende Pflegeheime kontaktiert werden, bis ein Pflegeplatz zu finden ist. Der Prozess erfordert auch viel Wartezeit	Herausforderung<br>Kommunikation<br>NAchsorger
STE-014	251-257	Herausfordernd ist, dass Pflegedienste in häuslicher Umgebung des Patienten erst herausgesucht werden und dann kontaktiert werden müssen	Herausforderung<br>Kommunikation<br>NAchsorger
STE-014	318-319	KI kann an Termine und Aufgaben erinnern	Chance
STE-014	318-321	Wunsch, dass KI Angehörige informiert	Chance<br>Kommunikation (zw. Akteuren)
STE-014	322-325	Zuständigkeit des Sozialdienstes liegt bei Rega und Sozialberatung. Sozialdienste in anderen Häusern übernehmen sowohl Pflegeüberleitung als auch Sozialdienst	Prozess<br>Zuständigkeit
STE-014	329-334	Pflege gibt Angehörigen die Telefonnummer des Sozialdienstes, weil sie dessen Zuständigkeit vermutet. Die ist aber im Bereich der KZP nicht gegeben, also muss TN den Angehörigen erklären, dass sie die falsche Ansprechpartnerin für ihn ist. Das ist zeitintensiv für MA und Angehörigen	Herausforderung<br>Zuständigkeit<br>Interd. Zusammenarbeit<br>Partizipation
STE-014	334-337	Wunsch, dass durch KI Zuständigkeit im EM für andere Berufsgruppen und Patienten/Angehörige transparenter ist. Dadurch können Fehler vermieden und Zeit gespart werden	Chance<br>Bedarf<br>Wunsch
STE-014	338-343	Angehörige rufen wiederholt die gleiche Person an, obwohl sie ihnen schon erklärt hat, dass sie nicht zuständig ist.	Herausforderung<br>Angehörige<br>Zuständigkeit
STE-014	345-349	Angehörige sind oft in emotionalen Ausnahmesituationen. Dadurch sinkt ihre Konzentrations- und Merkfähigkeit	Herausforderung<br>Angehörige
STE-014	349-351	Sinnvoll wäre, wenn KI direkt nach Kontakt mit Angehörigen und geklärtert Zuständigkeit die entsprechenden Anträge und Dokumente an diesen weiterleiten würde	Chance<br>Angehörige<br>Kommunikation
STE-014	355-360	Tafelvisite ist sehr zeitintensiv	Herausforderung<br>Interd. Zusammenarbeit
STE-014	389-393	Voraussetzung für weitere Tätigkeit/Dokumentation mit der Patientenakte sollte sein, dass mind. Ein Barthel-Index hinterlegt ist	Chance<br>Wunsch<br>Dokumentation

STE-014	396-399	Alle Einrichtungen arbeiten mit Barthel-Index. Pflegekräften ist nicht bewusst, wie wichtig er ist.	Herausforderung<br>Interd. Zusammenarbeit<br>Assessment
STE-014	410	Es fehlt einfach an Manpower	Herausforderung
STE-014	412-413	Wenn KI da ist, wird diese unterstützen	Einstellung<br>Hoffnung
STE-014	414-420	Andere Berufsgruppen interessieren sich nicht wirklich für Arbeitsweise des SD. Sie denken, sobald der Patient angemeldet ist, sei der Prozess abgeschlossen. Sie verstehen nicht, dass SD dann erst beginnen kann, die Tätigkeit aufzunehmen und die Zusammenarbeit mit vielen Berufsgruppen erforderlich ist	Herausforderung<br>Interd. Zusammenarbeit<br>Prozessverständnis
STE-014	478-499	TN äußert sich besorgt, wie der SD im Falle einer personellen Unterbesetzung mit den Deadlines innerhalb der Anwendung umgehen kann. Das Bedenken ist, dass die zu bearbeitenden Tätigkeiten durch die Personalsituation nicht entsprechend der Planung ausgeführt werden können. Daran schließt sich die Frage an, wie die KI mit einem solchem Umstand umgehen könnte, ob sie bspw. automatisch Schritte einleitet, um Deadlines entsprechend anzupassen	Ethische Bedenken<br>Sorgen
STE-015	42-44	Nicht jede Berufsgruppe braucht für ihre Tätigkeit die gleichen Informationen?	Prozess
STE-015	52-56	Information zu Gefahr der Verwahrlosung und Suchtproblematik sollte erhoben werden, da es ein Ausschlusskriterium für Rehas ist	Information<br>Bedarf - KI
STE-015	68-79	Es braucht auch Informationen zu Isolierpflicht. Oft müssen MA diese selber einholen	Herausforderung<br>KI – Bedarf<br>Information
STE-015	87-88	Es gibt einen Unterschied bei der Dokumentation zwischen den Stationen	Prozess
STE-015	91-94	Informationen zu Kontaktdaten warden nicht eingetragen. Im Diagnosefeld wird auf den Arztbrief verwiesen. Das führt dazu, dass MA sehr viel recherchieren müssen	Herausforderung<br>Dokumentation<br>Information<br>Interd. Zusammenarbeit
STE-015	96-99	Durch spärliche Dokumentation und Verweis auf Stationsakte müssen MA viel zu den entsprechenden Lagerorten laufen. Diese Wegzeit nimmt ihnen Ressourcen für andere Tätigkeiten	Herausforderung<br>Dokumentation<br>Information<br>Interd. Zusammenarbeit
STE-015	126-128	Kontaktdaten werden in Patientenaufnahme erhoben und in Aufnahmebrief geschrieben	Prozess

			Zuständigkeit
STE-015	136-137	In der pfleg. Anamnese werden Informationen (auch) nicht eingeholt	Herausforderung<br>Dokumentation<br>Information<br>Interd. Zusammenarbeit
STE-015	161-164	Angehörige kontaktieren SD und fragen, warum dieser sich nicht gemeldet hat. Sie haben ihre Nummer in der Akte auf der Station hinterlegt. Diese wurde von den Pflegekräften nicht ins System übertragen	Herausforderung<br>Dokumentation<br>Information<br>Interd. Zusammenarbeit<br>Angehörige
STE-015	171-189	Ärzte werden seitens der Organisation nicht wirksam aufgeklärt, welche Berufsgruppe welche Zuständigkeit hat.	Herausforderung<br>Zuständigkeit<br>Information<br>Bedarf an Information der BG
STE-015	197-210	Berufsgruppen haben wenig Kenntnis davon, welche andere Professionen im Haus vorhanden sind und was in deren Zuständigkeit fällt	Herausforderung<br>Zuständigkeit<br>Information<br>Bedarf an Information der BG
STE-015	244-250	KI sollte Daten auf Plausibilität miteinander im Entlassungsbericht prüfen und Warnhinweise gehen	KI - Bedar
STE-015	269-271	KLAU hat Struktur, die erfüllt werden muss, ansonsten kann der Auftrag nicht abgesandt werden	KLAU<br>Prozess
STE-015	271-282	Es kommt oft vor, dass KLAU bereits begonnen wird, aber nicht final bestätigt wird. In der Folge erreicht der die MA des SD nicht. Außerdem werden durch das Ausbleiben des EM weitere KLAUs angelegt	Herausforderung<br>Dokumentation<br>Information<br>Interd. Zusammenarbeit
STE-015	300-306	Folge des nicht korrekt angelegten KLAUs ist, dass weniger Zeit für die Organisation der Nachversorgung besteht und es zur Versorgungslücke kommen kann	Herausforderung<br>Dokumentation<br>Information<br>Interd. Zusammenarbeit<br>Kontinuität<br>Versorgung - Gefahr

STE-015	339-350,<br>370-381 ff	Arten von Anträgen, mit denen der SD in Kontakt kommt.	Dokumente<br>Anträge
STE-015	451-452	Frage der TN, wer in der Verantwortung steht, wenn in Folge einer Fehlentscheidung durch KI Schäden entstehen	Ethische Bedenken
STE-015	471-472	TN würde sich nie gänzlich auf KI verlassen	Einstellung
**Interview**	**Zeilen-Nr.**	**Generalisierung**	**Reduktion (auf Kategorie)**
STE-016	17-21	EM ist in der Chirurgie teilweise sehr herausfordernd, da dort häufig Obdachlose landen, die versorgt werden müssen	Herausforderung<br>Zuständigkeit – Abteilung<br>Prozess
STE-016	22-23	Wenn Patient selbst nicht in der Lage ist, zu telefonieren, müssen Ärzte eine gesetzliche Betreuung in die Wege leiten. Das wollen sie oft nicht machen	Zuständigkeit<br>Herausforderung<br>Bedarf – der gestzl. Betreuung<br>Interd. Zusammenarbeit
STE-016	27-31	MA der PÜ hat sich Textbausteine für ärztliche Verordnungen angelegt, damit sie Ärzten eine Vorlage zukommen lassen kann.	Dokumentation<br>Bedarf<br>Chance<br>Interd. Zusammenarbeit
STE-016	54-60	Kennt LLM durch ihren Sohn	Vorerfahrung KI
STE-016	72-75	TN ist interessiert an technischen Anwendungen, lernt schnell.	Interesse an KI<br>Bereitschaft zu Lernen-<br>Selbstkundgabe
STE-016	101-108	Obwohl Teile der Dokumentation schon vor Jahren digital angelegt wurden, wird immer noch vorrangig mit Papier gearbeitet und auf analoge Patientenakte verwiesen	Herausforderung<br>Dokumentation<br>Interd. Zusammenarbeit<br>Digitalsierung
STE-016	131-134	Technische Ausstattung ist mangelhaft, Headsets werden nicht genehmigt.	Herausforderung<br>Infrastruktur – Ausstattung<br>Kommunikation
STE-016	134-135	Mobiltelefon funktioniert auf der Station nicht, weil der Sendebereich unzureichend ist	Herausforderung<br>Infrastruktur – Ausstattung<br>Kommunikation

STE-016	185	Anforderung an KI: stellt alle entlassrelevanten Infos übersichtlich zusammen. Dazu gehört Pflegegrad des Patienten	Prozessbeginn<br>Bedarf<br>Anforderung KI<br>Information<br>Entlassrelevante Info
STE-016	190-194	Information, ob ein Pflegegrad vorliegt, muss zu Beginn des EM eingeholt werden. Falls keiner vorliegt, aber ein Pflegebedarf vorhanden ist, muss unmittelbar ein Antrag gestellt werden	Prozessbeginn<br>Information<br>Dokumentation<br>Bedarf
STE-016	194-	Eilantrag wird innerhalb von Sieben Werktagen per Aktenlage bestimmt. Normaler Pflegeantrag benötigt vier bis sechs Wochen Bearbeitungszeit	Prozessdauer – Beeinflussung<br>Pflegeantrag
STE-016	210-213	Private Krankenkassen geben grundsätzliche keine Informationen an SD	Akteur – PKV<br>Herausforderung
STE-016	214-215	Entlassrelevante Info ist, ob Pflegedienst vorhanden ist	Information<br>Entlassrelevante Info
STE-016	220	Entlassrelevante Info ist, ob Hilfsmittel vorhanden sind	Information<br>Entlassrelevante Info
STE-016	224-225	Entlassrelevante Info sind korrekte Kontaktdaten zu Bezugspersonen	Information<br>Entlassrelevante Info
STE-016	230-231	Als zuständiger Arzt ist meist Stationsarzt eingetragen	Arzt<br>Information<br>Prozess<br>Zuständigkeit
STE-016	239-240	TN muss erst herausfinden, wer der zuständige Arzt ist	Herausforderung<br>Zuständigkeit<br>Anforderung KI
STE-016	243-245	Entlassrelevante Info ist voraussichtliches Entlassdatum. Dieses muss nicht genau sein, eine ungefähre Einschätzung genügt	Information<br>Entlassrelevante Info
STE-016	248	Wenn Patienten von weiter weg kommen, wird Prozess des EM früher gestartet	Prozessbeginn<br>Einflussfaktor Prozess
STE-016	271	Entlassrelevante Info ist, wer der Hausarzt ist	Information<br>Entlassrelevante Info

STE-016	293-299	Wunsch ist, dass KI anhand der von der Pflege eingetragenen Informationen den Nachversorger ermittelt/vorschlägt, der benötigt wird.	Wunsch KI<br>Information<br>Prognose
STE-016	299-300	Station meldet sämtliche verfügbaren Optionen zur Nachsorge bei PÜ an. Diese weiß dann anhand der Anmeldung nicht, welcher Bedarf tatsächlich besteh	Herausforderung<br>Interd. Zusammenarbeit<br>Bedarf
STE-016	321-324	Vorschläge zur Umsetzung der Bedarfsermittlung von Nachsorgern	Interd. Zusammenarbeit<br>Bedarf
STE-016	341-345	Konkrete Vorschlag zur Anpassung der Optionen bei der Anmeldung zur Nachsorge: Einschränkung der verfügbaren Optionen zur Nachsorge aufgrund der Eingaben zum Patienten.	Wunsch KI
STE-016	358-361	Beim Aufnahmegespräch soll Assessment durchgeführt werden, das macht aber niemand	Herausforderung<br>Interd. Zusammenarbeit<br>Information<br>Assessment<br>Dokumentation
STE-016	451-456	Plausibilitätsprüfung bei Einträgen ist erforderlich	Bedarf KI
STE-016	474-482	Zu Beginn des EM werden Informationen hinsichtlich möglicher Kommunikationsbarrieren benötigt. Kogn. Einschränkungen, Einschränkungen im Hörverständnis, Sprachstörungen	Informationsbedarf<br>Prozessbeginn
STE-016	490-494	Wünschenswert wäre, dass KI solche Barrieren in der Kommunikation zwischen klinischen P. und Patient erkennt und sichtbar hervorhebt. Sollten Angaben dazu fehlen, soll KI eine Aufforderung zum Eintragne dieser Information geben, ggfs. Durch automatisches Aufsetzen einer Erinnerung oder Mail an die anschließende zuständige Pflegekraft	Wunsch KI
STE-016	514-516	Oft neue Anmeldung bereits gemeldeter Patienten bei Schichtwechsel	Herausforderung<br>KLAU
STE-016	521-523	Im Patientenorganizer kann man einsehen, ob ein Patient bereits angemeldet ist	Prozess<br>Information<br>Dokumentation
STE-016	528-530	Pflege liest Dokumentation der PÜ nicht	Herausforderung<br>Dokumentation<br>Interd. Zusammenarbeit
STE-016	596-597	Entlassrelevante Info ist Barthel-Index	Informationsbedarf

			Prozessbeginn
STE-016	604-608	PÜ erhält keine Information, wenn ein Patient verlegt wurde. Muss diese Information täglich selbst suchen	Herausforderung<br>Dokumentation<br>Interd. Zusammenarbeit<br>Kommunikation
STE-016	614-616	Wunsch ist, dass KI erkennt, wenn Patient verlegt wird und eine Benachrichtigung ausgibt	Anforderung KI
STE-017	3-7	Sozialarbeiter w3rden aus anderem Budget finanziert. PÜ wird über Pflege mit vergütet	Herausforderung<br>Finanzierung
STE-017	14-17	Headset wird nicht genehmigt. Dieses wird aber benötigt, da TN nicht telefonieren und dokumentieren gleichzeitig kann	Herausforderung - Ausstattung<br>Finanzierung<br>Dokumentation
STE-017	78-82	Sinnvoll wäre, wenn Angehörige oder Patienten durch Chatbot eine Liste mit Pflegediensten, Pflegestützpunkte in der häuslichen Umgebung erhalten	Chance KI
STE-017	96	Eine der Pflegeüberleitung häufig gestellt Frage ist, wie ein Pflegegrad beantragt wird	FAQ<br>Beratung
STE-017	163	Eine de Pflegeüberleitung häufig gestellt Frage ist, wie jemand Unterstützung zu Hause erhalten kann	FAQ<br>Beratung
STE-017	189-190	Eine de Pflegeüberleitung häufig gestellt Frage ist, wo Hilfsmittel zu finden sind	FAQ<br>Beratung
STE-017	205	Eine de Pflegeüberleitung häufig gestellt Frage ist, wie man an ein Rezept für ein Hilfsmittel gelangt	FAQ<br>Beratung
STE-017	211	Eine de Pflegeüberleitung häufig gestellt Frage ist, wie finanzielle Unterstützung erhalten werden kann	FAQ<br>Beratung<br>Finanzierung
STE-017	224	Eine de Pflegeüberleitung häufig gestellt Frage ist, wie eine Reha beantragt werden kann	FAQ<br>Beratung
STE-017	251	Eine de Pflegeüberleitung häufig gestellt Frage ist, wie ein Grad der Behinderung beantragt wird	FAQ<br>Beratung
STE-017	252-253	Eine de Pflegeüberleitung häufig gestellt Frage ist, ob ein Anspruch geltend gemacht werden kann	FAQ<br>Beratung
STE-017	264	Eine de Pflegeüberleitung häufig gestellt Frage ist, wie eine vollmacht erteilt werden kann	FAQ<br>Beratung

STE-017	302-306	Allgemein wird fast immer beraten zu Möglichkeiten der Versorgung zu Hause, Leistungen der Kranken- und Pflegeversicherung, Pflegegrad, Hilfsmittelversorgung, Hausnotrufsysteme und Hilfe zur Pflege	Beratung
STE-017	331-348	Eine Herausforderung ist, dass die PÜ/SD sich erstmal jedes angemeldeten Falls annehmen (müssen?). Durch die fehlende Bedarfsprüfung zu Beginn des Prozesses wird garnicht erhoben, welche Ressourcen der Patient und sein Umfeld innehaben. Würde die Einstellung seitens Krankenhaus als Institution von dieser Servicementatlität abrücken und die Patienten eher in die Selbstverantwortung nehmen, wäre das Arbeitsaufkommen um ei Vielfaches geringer	Herausfoderung<br>KLAU<br>Assessmen<br>Interd. Zusammenarbeit<br>Bedarf

Workshop 4

Interview	Zeilen-Nr.	Generalisierung	Reduktion (auf Kategorie)
STE-018	26	TN kennen Audiogenererierungen durch KI	Kenntnis<br>Vorerfahrung
STE-018	51-54	TN hat wenig Vorerfahrung mit KI	Kenntnis<br>Vorerfahrung
STE-018	100-103	Keine Auseinandersetzung mit dem Thema KI zuvor	Kenntnis<br>Vorerfahrung
STE-018	106-110	TN nimmt an, dass KI auf Informationseingabe durch Menschen angewiesen ist	Kenntnis<br>Vorerfahrung
STE-018	117-122	TN ist überrascht, dass KI die Fähigkeit besitzt, Entscheidungen zu treffen	Kenntnis<br>Einstellung
STE-018	131	TN sieht in KI auch ein Risikopotential	Einstellung KI
STE-018	178-180	Wünscht sich, dass KI die Anrufer nach Anliegen vorsortiert	Wunsch<br>Kommunikation
STE-018	207-211	Wunsch ist, dass manuelle Kontaktaufnahme zur Abfrage von Aufnahmen bei potentiellen Nachversorgern über KI erfolgt.	Wunsch<br>Nachversorger<br>Kommunikation
STE-018	217	Faxe und Telefonate sind aufwändig	Kommunikation<br>Herausforderung
STE-018	230-231	Barthel-Index soll automatisch erstellt und erfasst werden, wenn KLAU angelegt wird	KLAU<br>Assessment

			Wunsch
STE-018	231-232	Station muss oft zum Erstellen des Barthel-Index aufgefordert werden	KLAU<br>Assessment<br>Interd. Zusammenarbeit<br>Herausforderung
STE-018	359-361	TN erkundigt sich nach ethischen Aspekten beim Training der KI	Ethische Bedenken
STE-018	470-472	TN erfährt meist nur durch aktive Recherche in der Dokumentation von für sie relevanten Änderungen beim PAtienten	Herausforderung<br>Interd. Zusammenarbeit<br>Dokumentation<br>Bedarf<br>Information
STE-018	473-474	Wunsch über Benachrichtung sobald eine Veränderung beim Patienten eintritt	Wunsch<br>Bedarf<br>Information
STE-018	476-477	Zweifeln an Struktur bzw. Umsetzbarkeit innrehalb der Institution	Herausforderung - Struktur
STE-018	496-497	Bedarf an automatischer Information/Benachrichtigung bei Änderungen	Wunsch<br>Bedarf<br>Information
STE-018	500-501	Bei Fragen zu Patienten ist klinisches Personal für TN schwer erreichbar	Herausforderung<br>Interd. Zusammenarbeit<br>Dokumentation<br>Information
STE-018	509-512	Wollen nicht “alle” informationen sondern nur jene, die für EM umittelbar relevant sind	Herausforderung<br>Interd. Zusammenarbeit<br>Dokumentation<br>Information
STE-018	513-517	Sorge bzgl. Umsetzbarkeit der KI	Prozess<br>Strukturelle Herausforderung
STE-018	525-527	Entlassungsprozess wird letztlich durch Verfassung (und Willen?) des Patienten beeinflusst	Prozess<br>Herausforderung
STE-018	535	TN müssen Kontaktdaten suchen, weil sie nicht im System hinterlegt sind	Herausforderung<br>Interd. Zusammenarbeit<br>Dokumentation

			Information
STE-018	568-569	Viele Patienten und Angehörige geben an, dass sie keinen Computer besitzen	Herausforderung<br>Digitale Kompetenz<br>Akteure
STE-018	586	TN möchten sich eigentlich nicht mit KI oder damit verbundenen Veränderung auseinandersetzen	Einstellung – Selbstbekundung<br>Herausforderung
STE-018	588-589	TN zieht sich beim Thema Digitalisierung zurück	Einstellung – Selbstbekundung<br>Herausforderung
STE-018	592-594	Einschätzung zur Erreichbarkeit der Zielgruppe durch die Maßnahmen/Interventionen ist eher mäßig	Digitale Kompetenz<br>Akteur
STE-018	604-608	Jüngere Patienten haben ihr Smartphone immer bei sich. TN leitet sie an, sich eigenständig zu informieren und den Kontakt zur Krankenkasse herzustellen. Diese Patienten nehmen das positiv auf, weil sie dann während des Klinikaufenthalts Beschäftigung hätten	Digitale Kompetenz<br>Akteur<br>Partizipation
STE-018	620-624	Auch, wenn bei Klientel digitale Kompetenzen vorliegen/durch SD vermutet werden, hat die emotionale Belastung infolge der Erkrankung großen Einfluss auf das Ausmaß der Eigeninitiative zur Planung der Nachsorge	Herausforderung<br>Akteur<br>Partizipation
STE-018	626-628	Wenn TN Patienten auffordert, einen Pflegeantrag auszufüllen, tun das nur zwei von zehn. Die anderen acht möchten, dass die TN es macht	Herausforderung<br>Akteur<br>Partizipation
STE-018	642-643	Hilfe wird scheinbar oft konkret bei einer (Bezugs) Person gesucht	Akteur
STE-018	647	Großes Potnetial zur Entlastung des SD hat KI, die Anträge ausfüllt	Chance<br>Dokumentation
STE-018	674	In Notaufnahme werden fast nie Patientendaten dokumentiert	Herausforderung<br>Interd. Zusammenarbeit<br>Dokumentation<br>Information
STE-019	60-67	Beratung zu Versorgungsleistungen muss auf jeweilige Person und Situation abgestimmt sein. Faktoren wie Art der Versicherung, gewünschtes Pflegesetting und vorhandene Ressourcen bestimmen den Prozess maßgeblich	Beratung<br>Bedarf - Einflussfaktoren
STE-019	73-74	Mache Patienten/Angehörige wissen nicht, dass sie einen Pflegegrad selber beantragen können. Sie hinterlegen das Dokument beim Stationsarzt und warten darauf, dass dieser auf sie zukommt	Informationsdefizit<br>Patient/Angehörige

STE-019	107-111	Oft gestellte Fragen sind, ob Anspruch auf eine Haushaltshilfe besteht und wie diese beantragt werden kann	FAQ<br>Beratung
STE-019	123-126	Patienten, die zu geplanten OPs kommen, stellen erst nach dem Eingriff fest, dass sie Hilfe bei der Haushaltsführung benötigen. Ein Antrag dafür kann auch vor der OP aufgesetzt und bei der Krankenkasse eingereicht werden. Dann ist die Nachsorge auch zum Entlassungszeitpunkt abgesichert	Herausforderung<br>Nachsorge<br>Prozess<br>Kritischer Punkt
STE-019	128-131	KI kann bei geplanten Eingriffen eine mögliche Notwendigkeit von unterstützenden Leistungen ermitteln und Hinweise geben, sodass der Prozess vor Aufnahme ins KH geplant werden kann	Chance<br>Prozess
STE-019	152-153	Seniorenberatungsstellen und Pflegestützpunkte sind Akteure	Akteur
STE-019	160-164	Menschen beschäftigen sich mit Pflegebedürftigkeit und Vorsorge nicht, solange sie nicht unmittelbar davon betroffen sind	Herausforderung<br>Partizipation<br>Vorsorge
STE-019	168-171	Infolge eines Informationsdefizits beziehen die Personen u.a. Leistungen von Sanitätshäusern, bei denen die Kosten nicht von der Krankenkasse übernommen werden	Herausforderung<br>Partizipation<br>Vorsorge<br>Finanzierung
STE-019	214-217	Informatiosndefizit/fehlende Beratung?<br>Die Zuständigkeit für das jeweilige Anliegen der Versicherten ist für diese nicht immer nachvollziehbar. Sie wenden sich oft an den Leistungserbringer, mit dem sie zuletzt in Kontakt waren	Information<br>Beratung<br>Hilfsmittel<br>Bedarf
STE-019	262	Weitere Akteure sind Rentenversicherung und Beihilfestellen	Akteur
STE-019	278-284	Manche Privatpatienten sind nicht im Bilde darüber, welche Leistungen ihre Police abdeckt	Finanzierung<br>Krankenkassen<br>Herausforderung
STE-019	285-286	SD kann nicht alle Angelegenheiten selbst übernehmen. Gerade bei privat Versicherten haben sie keinen Einblick in die Vertragsbestandteile	Krankenkassen<br>Herausforderung<br>Kommunikation
STE-019	298-313	Sonderfall des Versicherungstatus<br>Besondere Konstellationen der Versicherungen erschweren die Versorgung (Bsp. Krankenversicherung gesetzlich, Pflegeversicherung privat)	Krankenkassen<br>Herausforderung

STE-019	344-346	Betreuungsverfahren als Folge von geschäftsunfähigkeit und fehlender Betreuungsverfügung	Prozess<br>Herausforderung<br>Information<br>Dokumentation
STE-019	354-355	Es kommt vor, dass sich erst zum Ende des EM-Prozesses herausstellt, dass der/Die Patient:in einen Betreuer hat.	Prozess<br>Herausforderung<br>Information<br>Dokumentation<br>Interd. Zusammenarbeit
STE-019	376-377	Manchmal wird erst kurz vor der Entlassung festgestellt, dass ein Patient nicht geschäftstüchtig ist.	Prozess<br>Herausforderung<br>Information<br>Dokumentation<br>Interd. Zusammenarbeit
STE-019	385-386	Sinnvoll wäre, das einmal dkokumentierte Angaben in der Patientenakte bei einem erneuten Aufenthalt übertragen werden	Chance<br>Dokumentation
STE-019	387-390	Aktuell werden analoge Daten in Papierakte hinterlegt und sind beim nächsten Aufenthalt des Patienten nicht mehr zielsicher auffindbar	Herausforderung<br>Dokumentation
STE-019	398-401	Bei manchen Patieten sind Kontakdaten hinterlegt, die nicht mehr aktuell sind	Herausforderung<br>Dokumentation
STE-019	447-453	Bestehende Rahmenverträge zwischen Krankenkassen und Leistungserbringern wie Transportdiensten sind nur begrenzt gültig. Diese Daten werden nicht automatisch aktualisiert	Herausforderung<br>Dokumentation<br>Finanzierung<br>Information<br>Chance<br>Hilfsmittel
STE-019	492-496	TN äußert Bedenken zur Datensicherheit der KI	Ethische Bedenken
STE-019	542-544	Wenn TN keine Kontaktdaten von Angehörigen haben oder auffinden können, legen sie ihre Kontaktdaten ins Patientenzimmer und bitten um Anruf	Information<br>Dokumentation<br>Prozess<br>Kommunikation
STE-019	552-553	Erleichterung wäre, wenn sich alle bestehenden Daten automatisch aktualiseren	Dokumentation<br>Information

			Bedarf KI
STE-019	560-562	TN äußert Bedenken, dass Patienten und Angehörige den Einsatz von KI im EM schwer bis nicht annehmen, weil sie menschlichen Austausch bevorzugen	Ethische Bedenken<br>Herausforderung<br>Sorge
STE-019	565-571	Anforderung an KI ist, das diese die benötigten Anträge und Formulare mit den vorhandenen Patientendaten vorab ausfüllt	Bedarf<br>Anträge<br>Chance<br>Dokumentation<br>Herausforderung
STE-019	576-583	Manuelles Ausfüllen der Anträge ist enormer Zeitaufwand	Bedarf<br>Anträge<br>Chance<br>Dokumentation<br>Herausforderung
STE-019	592	Wunsch nach Interoperabilität	Wunsch<br>Bedarf<br>Dokumentation
STE-019	634-639	Es kam in der Vergangenheit vor, dass Faxe nicht bei der Krankenkasse ankamen und eine Bewilligung in der Folge nicht rechtzeitig erfolgt	Herausforderung<br>Finanzierung<br>Kommunikation
STE-019	645-646	Wunsch ist, dass Patienten eine Erstinformation durch KI erhalten und dadurch gelassener sind	Information<br>Partizipation

Workshop 5			
**Interview**	**Zeilen-Nr.**	**Generalisierung**	**Reduktion (auf Kategorie)**
Workshop 5	52	KI wird durch Mensch entwickelt, kann sich eigenständig weiterentwickeln	Einstellung<br>Kenntnis/Annahme zu KI
Workshop 5	58	Hat ChatGPT bereits genutzt	Vorerfahrung zu LLM
Workshop 5	96	Künstliche Unterstützung, Spracherstellung	Einstellung<br>Kenntnis/Annahme zu KI

Workshop 5	102-103	Algorithmus, der ein neuronales Netz simuliert	Einstellung<br>Kenntnis/Annahme zu KI
Workshop 5	106	Programm, das Arbeit abnehmen und Texte schreiben soll	Einstellung<br>Kenntnis/Annahme zu KI
Workshop 5	115	Teilersatz des menschlichen Gehirns	Einstellung<br>Kenntnis/Annahme zu KI
Workshop 5	127-128	TN möchte wissen, ob KI menschliche Emotionen und Wahrnehmung ersetzen kann	Einstellung<br>Kenntnis/Annahme zu KI<br>Interesse
Workshop 5	135-137	Fragt sich, ob KI, wie ein Mensch auch, die persönlichen Eindrücke zum Patienten wahrnehmen kann	Einstellung<br>Kenntnis/Annahme zu KI<br>Interesse
Workshop 5	147-152	Berufserfahrung ist von großer Bedeutung und nicht mit Fachwissen gleichzusetzen. Erfahrenere Mitarbeiter sind u.U. stärker sensibilisiert.<br>KI kann Gesamtsituation nicht so wahrnehmen und deuten, wie ein Mensch	Einstellung<br>Grenzen (Wahrnehmung)
Workshop 5	159-161	Grenzen von KI liegen auch in ihrem Training	Einstellung<br>Grenzen (Wahrnehmung)
Workshop 5	171-174	TN fragt, wie KI unterstützen kann, sieht unüberwindbare Grenzen der Einsatzmöglichkeit in Wahrnehmung/Erfassung komplexer Situationen	Grenzen<br>Herausforderung
Workshop 5	202-203	Kann unterstützen. Arbeit abnehmen. Kann nicht menschlichen Kontakt ersetzen	Einstellung<br>Grenzen
Workshop 5	212	Informationen rausfiltern	Einstellung<br>Chancen
Workshop 5	213	KI kann nicht Empathie empfinden	Einstellung<br>Grenzen
Workshop 5	231	Selbstverständnis des SD als empathischen Beruf	Akteur SD
Workshop 5	256	Wie KI bei Arbeit helfen kann: Prozesse zusammenführen, verbessern, erleichtern	Chance
Workshop 5	257	Wie KI bei Arbeit helfen kann: Texte rasch erstellen und auswerten	Chance
Workshop 5	262-264	Wie KI bei Arbeit helfen kann:<br>Wenn er Arztbriefe ausgeben kann	Chance

Workshop 5	266-267	Wie KI bei Arbeit helfen kann:<br>Wenn KI Befehle befolgt „schreibe zwei Seiten“ oder „Fasse Text zusammen“	Chance<br>Vorkenntnisse<br>Einstellung
Workshop 5	289-294	TN hat im Rahmen einer Hausaufgabe für ihr Kind ChatGPT genutzt	Vorkenntnis<br>Anwendung
Workshop 5	393-394	KI kann unterstützend bei Personalmangel wirken	Chance<br>Hoffnung
Workshop 5	394-398	Herausforderung liegt darin, Menschen nicht zu ersetzen. KI kann nicht so professionell handeln wie ein Mensch. Sorge, dass Einsatz der KI als Argument für Personaleinsparung genutzt wird	Grenzen<br>Sorgen/Bedenken<br>Ethische Bedenken<br>Herausforderung
Workshop 5	454	Ohne Trainingsdaten keine KI möglich	Grenzen<br>Sorgen/Bedenken<br>Ethische Bedenken<br>Herausforderung
Workshop 5	457-458	Fehlerhafte Trainingsdaten	Grenzen<br>Sorgen/Bedenken<br>Ethische Bedenken<br>Herausforderung
Workshop 5	538-541	Möchte wissen, wie KI an Informationen gelangen kann, die sonst durch menschliche Wahrnehmung entstehen	Grenzen<br>Sorgen/Bedenken<br>Ethische Bedenken<br>Herausforderung
Workshop 5	542-550	Hilfreich wäre, wenn KI komplette Planung des EM und der Nachsorge autonom vornehmen würde. Je nach Ausgangslage des Pat. Und Erkrankung soll die KI den dafür prädestinierten Weg inkl. Beratungsinhalte vorzeichnen, sodass SD die Informationen nur noch aufnehmen und kommunizieren muss	Wünsche<br>Standardisierung<br>Beratung
Workshop 5	568-571	Berufsgruppen haben nicht alle die gleichen Prozesse	Prozessabläufe<br>Bedarf Patienten<br>Beratung
Workshop 5	582-594	Anforderungen von Stationen gehen digital an SD. Dieses Konsil wird ausgedruckt und handschriftlich weiter zur Dokumentation genutzt. Diese Informationen werden dann im IKT	Prozessdefinition/-ablauf<br>Doku

		übernommen, sodass andere MA auch Einsicht haben. Es sind Textbausteine durch SD eingerichtet, die bei Bedarf genutzt werden	Daten/Infos<br>Akteure
Workshop 5	596-598	Textbausteine sind unterteilt nach Arten von Kostenträgern, Arten von Reha-Verfahren und Arten von Hilfsmitteln, die beantragt wurden	Doku<br>Daten/Infos<br>Akteure<br>Standardisierung
Workshop 5	607	SD hat sich selbst Textbausteine erstellt<br><br>Bedarf (zeigt sich dadurch)	Doku<br>Daten/Infos<br>Akteure<br>Wunsch nach Standardisierung<br>Bedarf
Workshop 5	640	Patient als Stakeholder	Akteur<br>Prozess
Workshop 5	644	Möglichkeiten der Partizipation des Pat. Durch Annahme oder Ablehnen des EM	Akteur<br>Prozess<br>Partizipation
Workshop 5	647	Es gibt Patienten, die EM regelrecht einfordern	Akteur<br>Prozess<br>Wunsch<br>Bedarf<br>Partizipation
Workshop 5	650	Angehörige und Betreuer	Akteur<br>Prozess
Workshop 5	652	Sorgeberechtigte	Akteur<br>Prozess
Workshop 5	670	Ärzte und Pfleger	Akteur<br>Prozess
Workshop 5	680-681	Physiotherapie, Pflegeüberleitung und Reha	Akteur<br>Prozess
Workshop 5	697	Hilfsmittel-Lieferanten, KK, Pflegedienste	Akteur<br>Prozess

Workshop 5	704	Sanitätshäuser	Akteur<br>Prozess
Workshop 5	706	Apotheken	Akteur<br>Prozess
Workshop 5	707	Weiterbehandelnde Ärzte	Akteur<br>Prozess
Workshop 5	708	Externe Praxen	Akteur<br>Prozess
Workshop 5	710	Einrichtungen	Akteur<br>Prozess
Workshop 5	712	Pflegeheime	Akteur<br>Prozess
Workshop 5	722-723	Sozialpsychiatrischer Dienst und ambulante psychiatrische Pflege	Akteur<br>Prozess
Workshop 5	733	Spezialisierte ambulante palliative PFlege	Akteur<br>Prozess
Workshop 5	736	Hospize	Akteur<br>Prozess
Workshop 5	750	Betreutes Wohnen	Akteur<br>Prozess
Workshop 5	773-774	Spezialanbieter für parenterale Ernährung, Stoma-Versorgung und Tracheostoma	Akteur<br>Prozess
Workshop 5	777-778	Es gibt direkte und indirekte Stakeholder	Akteur<br>Prozess<br>Partizipation
Workshop 5	801-803	Bedeutung der beteiligte Akteure hängt von Bedarf der Patienten ab	Akteur<br>Prozess<br>Bedarf
Workshop 5	807-818	Ausmaß des Engagements bzw. der Partizipation von Angehörigen bestimmt unmittelbar die Arbeitslast der SD im EM	Akteur<br>Prozess<br>Bedarf<br>Herausforderung<br>Partizipation

Workshop 5	834	Rentenversicherung	Akteur<br>Prozess
Workshop 5	835	Sozialamt	Akteur<br>Prozess
Workshop 5	837	Private Versicherungen, private Kostenträger	Akteur<br>Prozess
Workshop 5	838	Unfallträger	Akteur<br>Prozess
Workshop 5	840	Jugendamt	Akteur<br>Prozess
Workshop 5	844	Berufsgenossenschaft	Akteur<br>Prozess
Workshop 5	912-913	Meiste Herausforderungen gibt es in der Zusammenarbeit mit Ärzten	Herausforderung<br>Akteure<br>Interd. Zusammenarbeit
Workshop 5	930-931	Meiste Punkte sind bei Ärzten, zweite Stelle ist Kostenträger	Herausforderung<br>Akteure<br>Interd. Zusammenarbeit
Workshop 5	933-934	Ärzte versprechen Pat. Dinge, die gesetzlich nicht bestand haben	Herausforderung<br>Akteure<br>Interd. Zusammenarbeit<br>Information<br>Beratung
Workshop 5	936-939	Ärzte versprechen Pat. Dinge, die gesetzlich nicht bestand haben	Herausforderung<br>Akteure<br>Interd. Zusammenarbeit<br>Information<br>Beratung
Workshop 5	946	Ärzte führen eine vermeintliche Beratung der Patienten durch bzw. stellen diesen Versorgungsmaßnahmen in Aussicht, die so nicht umsetzbar sind	Herausforderung<br>Akteure<br>Interd. Zusammenarbeit<br>Information<br>Beratung

Workshop 5	953-956	SD berät und empfiehlt Patienten zu der Versorgungsform, die er bedarf und die auch faktisch umsetzbar ist	Prozess<br>Information<br>Beratung
Workshop 5	966-968	Herausforderung in der Interd. Zusammenarbeit. Professionen verfolgen eigene Ziele. Hier stehen sie in direktem Konflikt	Herausforderung<br>Akteure<br>Interd. Zusammenarbeit
Workshop 5	969-971	Führt dazu, dass falsche Auskünfte gegeben und Versprechen gemacht werden. Daraus resultieren Diskussionen zwischen Pat. Und SD	Herausforderung<br>Akteure<br>Interd. Zusammenarbeit<br>Beratung<br>Information
Workshop 5	980-986	Patienten werden oft entlassen, weil deren Pflegebett benötigt wird. Dies geschieht, ohne das mit zuständiger SD-MA abzusprechen. Diese kann ihre Beratung nicht vollständig durchführen.	Herausforderung<br>Kommunikation<br>Beratung<br>Kritischer Punkt - Versorgungsbu<br>Interd. Zusammenarbeit
Workshop 5	989	Interessen kollidieren eben	Herausforderung<br>Akteure<br>Interd. Zusammenarbeit<br>Beratung<br>Information
Workshop 5	996	Großes Problem ist Erreichbarkeit von Ärzten	Herausforderung<br>Akteure<br>Interd. Zusammenarbeit
Workshop 5	1002-1006	SD hat keine festen zuständigen Ansprechpartner im Bereich der Ärzte. Verantwortlichkeiten wechseln mitunter sehr schnell. Ärzte verweisen auf andere Zuständigkeit, Prozess des EM wird dadurch insgesamt negativ beeinflusst	Herausforderung<br>Akteure<br>Interd. Zusammenarbeit<br>Zuständigkeit
Workshop 5	1018-1023	Kommunikation mit Kostenträgern herausfordrend. Konkrete Ansprechpartner sind nicht leicht ausfindig zu machen. Nicht ausreichend besetzte Hotlines erschweren die Recherche	Herausforderung<br>Akteure<br>Interd. Zusammenarbeit

			Zuständigkeit<br>Prozess
Workshop 5	1029-1030	i.d.R. kein fester Kontakt bei KK für SD	Herausforderung<br>Akteure<br>Prozess<br>Interd. Zusammenarbeit<br>Zuständigkeit<br>Kommunikation
Workshop 5	1032-1034	DRV-Bund hat feste Zuständigkeit und Hotline für Sozialdienste.	Akteure<br>Prozess<br>Interd. Zusammenarbeit<br>Zuständigkeit<br>Kommunikation
Workshop 5	1039-1042	Wunsch an KI, dass diese Kommunikation mit Kostenträgern unterstützt indem sie direkt Verbindung herstellt	Chance<br>Wunsch<br>Bedarf<br>Kommunikation
Workshop 5	1050-1053	SD ruft bei Kostenträgern an, um eine Beschleunigung in laufenden Verfahren zu erreichen. Tlw. Werden Kostenzusagen sehr dringend benötigt	Herausforderung<br>Kommunikation<br>Finanzierung
Workshop 5	1057-1059	In 90-95% der Fälle ist eine Zusage zur Kostenübernahme gegeben	Finanzierung
Workshop 5	1069-1073	Privatkassen und Beihilfen sind zu 99% nicht zu erreichen. Sprechen auch nicht mit SD, nur mit Patienten. Kostenübernahmebescheide senden sie ausschließlich an Anschrift des Patient	Finanzierung<br>Herausforderung
Workshop 5	1078-1084	Bei bestimmten Krankheitsbildern oder Nachsorgebedrafen kommt es vor, dass SD große Schwierigkeiten hat, einen geeigneten Nachversorger mit Aufnahmekapazität zu finden. Dann wenden se sich an Kostenträger mit Bitte um Unterstützung. Es wäre eine Erleichterung, wenn KI bei solchen Verfahren automatisiert eine	Krischer Punkt<br>Kontinuität der Versorgung<br>Versorgung<br>Chance<br>Hoffnung<br>Wünsche
Workshop 5	1097-1103	Angehörige sind oft fordernd. Möchten in kurzer Zeit Versorgungslösungen. Sie sind auch verärgert, wie Ärzte Versprechungen machen, die der SD nicht einhalten kann.	Angehörige<br>Herausforderung<br>Versorgung

		Angehörige haben wenig Verständnis	
Workshop 5	1109-1113	Angehörige informieren sich im Internet. Haben Anspruchshaltung und kommen aktiv mit Forderungen auf SD zu.<br>KK sagen Angehörigen, dass SD verantwortlich ist.	Angehörige<br>Herausforderung<br>Versorgung
Workshop 5	1126-1130	Angehörige wirken oft hilflos und uninformiert. MA klärt dann zu Möglichkeiten der Versorgung auf	Beratung<br>Informationsdefizit<br>Angehörige<br>Versorgung (überfordert Angehörige)
Workshop 5	1133-1134	Angehörige werden ungehalten. SD beendet dann Gespräch	Angehörige<br>Herausforderung<br>Versorgung<br>Prozess (hemmender Faktor)
Workshop 5	1140-1145	Lösungsansatz für Beratung kann sein, dass KI für Patienten und Angehörige Informationen bereitstellt, die bereits in Beratungsgespräch mit SD besprochen wurden. SD muss Themen mehrmals besprechen, da Pat./A. dies innerhalb eines Gespräches nicht alles fassen können	Beratung<br>Herausforderung<br>Chance<br>Prozess<br>Information
Workshop 5	1149	KK bieten schon solche Beratungen an, dennoch entstehen die Fragen im Akutsetting Krankenhaus.	Beratung<br>Herausforderung<br>Chance<br>Prozess<br>Information
Workshop 5	1150-1156	Aus Sicht dieser TN bietet sich eine Beratung je nach Setting an.	Beratung<br>Bedarf<br>Herausforderung<br>Chance<br>Prozess<br>Information
Workshop 5	1157-1161	Wunsch wäre, dass KI Pat./Angehörige darauf hinweist, wie EM eingeleitet wird.<br><br>Patienten/Angeh. Sind unwissend über den Prozess des EM und die Arbeitsweise. Folge sind hohe Anzahl an Kontaktversuchen zu SD. Das wiederum belastet diesen.	Beratung<br>Bedarf<br>Herausforderung<br>Chance

			Prozess<br>Information<br>Partizipation
Workshop 5	1178-1180	Es könnte Werkzeug generiert werden, dass Bedarf der Pat. Abfragt und zu diesem Auskunft gibt	Bedarf<br>Chance<br>Beratung
Workshop 5	1186-1188	KI soll Bedarf der Pat. Abfragen und zu diesem Auskunft per Mail weiterleiten	Bedarf<br>Chance<br>Beratung<br>Kommunikationsweg
Workshop 5	1207-1209	Zahl der Anrufe sehr hoch, erfordert viel Personalressourcen zur Koordinierung	Herausforderung<br>Kommunikation
Workshop 5	1244-1245	Plätze zur Nachversorgung im Pflegeheim sind schwer zu beschaffen	Nachversorgung<br>Herausforderung<br>Kritischer Punkt
Workshop 5	1251-1253	Pflegeheim-Ampel zur Symoblisierung der Auslastung von Einrichtungen gibt es schon, hilft aber nicht, da es allgemein kaum Kapazitäten gibt	Nachversorgung<br>Herausforderung<br>Kritischer Punkt
Workshop 5	1255-1259	Hohe Auslastung des Gesundheitssystems und dadurch knappes Angebot als strukturelle Herausforderung für EM	Nachversorgung<br>Herausforderung<br>Kritischer Punkt
Workshop 5	1265-1267	Patient muss im Zweifel nach Hause, wenn er keinen Nachversorger hat	Nachversorgung<br>Herausforderung<br>Kritischer Punkt
Workshop 5	1273-1279	Klinikinterne Prozesse sind nicht aufeinander abgestimmt. Prozess der OP bedarf eigentlich der Abstimmung mit Prozess des EM, wird aber nicht vorgenommen. So kommt es Herausforderungen bei der Nachsorge und Versorgungsbruch.	Prozess<br>Interd. Zusammenarbeit<br>Beratung<br>Herausforderung<br>Kritischer Punkt
Workshop 5	1282-1285	Im Kern entsteht ein Problem daraus, dass Patienten vor Eingriffen nicht umfangreich aufgeklärt/beraten werden	Prozess<br>Interd. Zusammenarbeit<br>Beratung<br>Herausforderung

			Kritischer Punkt
Workshop 5	1286-1287	Bei korrekter Afklärung vor med Eingriff hat der Patient noch die Chance, die Nachsorge selbst mitzugestalten.<br>Im Kern entsteht ein Problem daraus, dass Patienten vor Eingriffen nicht umfangreich aufgeklärt/beraten werden	Partizipation<br>Prozess<br>Interd. Zusammenarbeit<br>Beratung<br>Herausforderung
Workshop 5	1293-1296	Resultat des oben beschriebenen Vorgehens ist, dass Pat. Sich einer geplanten OP unterziehen und danach verwundert sind, dass nicht sofort eine Nachsorge gewährleistet ist	Partizipation<br>Prozess<br>Interd. Zusammenarbeit<br>Beratung<br>Herausforderung<br>Nachsorge<br>Kontinuität
Workshop 5	1297-1300	Sinnvoll könnte sein, dass KI den OP-Plan beobachtet und lernt, welche Eingriffe sehr Nachsorgeintensiv sind. Außerdem könnte dadurch die Nachversorgung besser abgestimmt werden.	Chance<br><br>Nachsorge<br>Kontinuität<br>Prozess - Planung
Workshop 5	1310-1313	Prozessoptimierung innerhalb des EM muss bereits vor Aufnahme ins KH ansetzen	Nachsorge<br>Bedarf<br>Kontinuität<br>Prozess - Planung
Workshop 5	1321-1323	Unterschiedliche Prozessplanung in Bereichen der Ortho und Kardio	Prozess
Workshop 5	1323-1327	Problem ist oft, dass EM geplant ist, der Patient dann aber verlegt wird und SD darüber nicht informiert wird. KI wäre hier hilfreich	Herausforderung<br>Interd. Zusammenarbeit<br>Prozess<br>Chance
Workshop 5	1523-1525	Prozessbeginn ist hier das Konsil. Es gibt eine MSDN-Anforderung	Prozessbeginn<br>KLAU
Workshop 5	1534-1535	Zuständigkeit des SD ist nach Organisationseinheit geregelt	Zuständigkeit<br>Prozess

Workshop 5	1546-1548	Ärzte haben Möglichkeit, dringende Anforderungen an den SD zu stellen.	Interd. Zusammenarbeit<br>KLAU<br>Prozess
Workshop 5	1553-1554	Möglichkeit der Markierung der hohen Dringlichkeit wird häufiger genutzt, als es nach Ansicht des SD erforderlich wäre	Interd. Zusammenarbeit<br>KLAU<br>Prozess
Workshop 5	1564-1567	Nächster Prozessschritt ist Kontrolle der Zuständigkeit	Zuständigkeit<br>Prozessschritt
Workshop 5	1579-1586	Oft kommt es dazu, dass die Anforderungen an den falschen Ansprechpartner gesandt werden. Das liegt daran, dass es mehrere Sozialarbeiter gibt. Manche gehören fest zu Organisationseinheiten.	Zuständigkeiten<br>Herausforderung
Workshop 5	1596-1598	Dringlichkeit des EM bzw. der Nachsorgeplanung lässt sich klassifizieren. Priorität sind kurzfristige Entlassungen und Palliativpatienten, die in der letzten Sterbephse sind	Prozess<br>Versorgung
Workshop 5	1600	Kurzzeitpflegeplätze sind nie dringend, weil man lange warten muss	Prozess<br>Versorgung<br>Nachsorge
Workshop 5	1603-1604	Für Arzt ist es dringend, weil er Pat. Entlassen will	Herausforderung<br>Zielkonflikt<br>Akteur Arzt
Workshop 5	1616-1617	Wer letztlich wann entlassen wird, entscheidet der Oberarzt	Herausforderung<br>Zielkonflikt<br>Akteur Arzt
Workshop 5	1627-1632	KI könnte Markierung als dringlichen Fall unterbinden<br>Lösungsvorschlag für inflationären Gebraucht der Dringlichkeits-Markierung	Wunsch<br>Chance
Workshop 5	1637-1643	Unklarheit darüber, welche Berufsgruppe für den Bedarf des Pat. zuständig ist führt dazu, dass falscher Mitarbeitende eingeschaltet wird<br><br>KI kann hier bei Anforderung der richtigen Profession unterstützen	Herausforderung<br>Zuständigkeit<br>Chance<br>Prozess
Workshop 5	1652-1655	Wenn unzureichende Informationen zum Pat. vorliegen, können Mitarbeitende nicht einschätzen, welche Profession tätig werden muss	Herausforderung<br>Informationen<br>Prozess

Workshop 5	1661	Wichtige Informationen, die oft fehlen, sind Angaben zu Angehörigen und Betreuung sowie die Hinterlegung des Auftrags.	Herausforderung<br>Benötigte Informationen<br>Prozess<br>KLAU
Workshop 5	1662-1663	Angehörige, Betreuer	Benötigte Informationen<br>Herausforderung (weil Infos fehlen)<br>Prozess
Workshop 5	1664-1667	Bedeutend ist auch zu wissen, ob ein Patient kognitiv beeinträchtigt ist. Diese Information wünschen sich MA zu Beginn des Prozesses	Benötigte Informationen<br>Herausforderung (weil Infos fehlen)<br>Prozess
Workshop 5	1684-1688	Einsatzmöglichkeit für KI im Bereich der Bedarfsermittlung bei Hilfsmitteln.<br>KI könnte anhand der Diagnose und Abteilung Hilfsmittel vorschlagen, die oft in dem Kontext benötigt werden. MA kann dann damit ins Beratungsgespräch gehen und hat eine Entlastung in der Vorbereitung	Wunsch<br>Chance<br>Versorgung – Hilfsmittel<br>Beratung
Workshop 5	1694-1697	Wunsch, dass KI vor Beratungsgespräch schon relevante Aspekte strukturiert ausgibt und als Schema für Gespräch genutzt werden kann	Wunsch<br>Chance<br>Beratung
Workshop 5	1700-1703	Möchte von KI relevante Unterlagen für Patientengespräche vorbereiten bekommen	Wunsch<br>Chance<br>Beratung
Workshop 5	1707-1710	KI könnte Fragenkatalog anhand von Schlüsselwörtern in der ePA generieren und sich Pat.-Daten aus System ziehen, um entsprechende Anträge vorzubereiten	Wunsch<br>Chance<br>Beratung<br>Daten<br>Informationen<br>Prozess
Workshop 5	1714-1716	KI soll doppelte Anforderungen/Aufträge an SD erkennen und Duplikate unterbinden. Erneute Erteilung des inhaltlich selben Auftrags verursacht Mehraufwand	Herausforderung (weil Infos fehlen)<br>Prozess<br>Chance

Workshop 5	1730-1734	KI sollte bei gewissen Informationen über den Pat. Einen bestimmten Prozessablauf vorgeben, der an diese speziellen Anforderungen angepasst ist	Wunsch<br>Chance
Workshop 5	1747-1750	Wenn MA keine Kenntnis über demenz. Erkrankung eines Pat. Haben verlängert und erschwert das den Prozess	Benötigte Informationen<br>Herausforderung (weil Infos fehlen)<br>Prozess
Workshop 5	1772-1774	Teilweise können Informationen über Gesundheitszustand und Versorgungsbedarfe aus Maßnahmen abgeleitet werden	Benötigte Informationen<br>Herausforderung (weil Infos fehlen)<br>Informationen<br>Prozess
Workshop 5	1774-1775	Em braucht Informationen, muss sie selbst einholen	Benötigte Informationen<br>Herausforderung (weil Infos fehlen)<br>Prozess
Workshop 5	1796	Nächster Prozessschritt ist Kontaktaufnahme zum Patienten	Prozessschritt
Workshop 5	1807	Beim Patienten werden Notizen angefertigt	Prozessschritt
Workshop 5	1815-1816	KI sollte automatisch benachrichtigen, wenn Pat. Verlegt wird	Chance<br>Wunsch<br>Information
Workshop 5	1817-1821	EM wird oft nicht über Verlegung des Patienten informiert und muss ihn dann suchen	Kommunikation<br>Information<br>Herausforderung<br>Informationen<br>Daten
Workshop 5	1827	Innerhalb der Anforderung des EM wird der neue Aufenthaltsort des Pat. Nicht aktualisiert	Kommunikation<br>Information<br>Informationen<br>Daten

			Herausforderung<br>KLAU
Workshop 5	1828-1829	automatische Aktualisierung im Auftrag der Anforderung von Dokumenten wo möglich	Kommunikation<br>Information<br>Informationen<br>Daten<br>Herausforderung<br>KLAU<br>Chance
Workshop 5	1832-1835	Konsil wird ausgedruckt, Termin beim Patienten mitunter Tage später. Gedruckte Informationen sind dann nicht mehr aktuell	Kommunikation<br>Information<br>Informationen<br>Daten<br>Herausforderung<br>Prozess
Workshop 5	1839-1841	Insbesondere Patienten der Intensivstation sind davon betroffen	Kommunikation<br>Information<br>Informationen<br>Daten<br>Herausforderung<br>Prozess
Workshop 5	1859-1860	Manche Pat. Sprechen kein Deutsch, dann muss ein Dolmetscher gesucht werden	Kommunikation<br>Information<br>Informationen<br>Daten<br>Herausforderung<br>Prozess
Workshop 5	1866-1867	Information wird benötigt, welche Sprache der Pat. spricht	Kommunikation<br>Information<br>Bedarf<br>Informationen<br>Daten<br>Herausforderung

			Prozess
Workshop 5	1922-1924	Nächster Prozessschritt ist Bedarfserhebung anhand der gesammelten Informationen.	Prozessschritt<br>Bedarf
Workshop 5	1941	Was unterschreibungspflichtig ist, muss unterschrieben werden	Prozessschritt
Workshop 5	1977-1978	MA hoffen, dass mobile Endgeräte zur Dokumentation am Patientenbett bald kommen	Wunsch<br>Einstellung<br>Dokumentation
Workshop 5	1983-1990	Häufig können Patienten ihre eigenen Bedarfe nicht nennen. SD nimmt dann Kontakt zu Angehörigen auf, um mit diesen die Bedarfe zu besprechen. Dies geschieht tlw. Gemeinsam mit Pat.	Herausforderung<br>Bedarf<br>Kommunikation<br>Partizipation
Workshop 5	2020-2025	Nächster Prozessschritt ist suchen eines geeigneten Nachversorgers	Prozessschritt
Workshop 5	2027-2031	Problem ist, dass Nachversorger nicht die gleiche Software nutzen. Über einzelne Kontaktanfragen per Mail oder Telefon geben sie zuverlässig Auskunft	Herausforderung<br>(Systembruch)<br>Kommunikation<br>Nachsorge<br>Kritischer Punkt
Workshop 5	2043-2044	Lösungsvorschlag für KI. Automatisierte Anfrage für Nachversorger	Chance<br>Kommunikation<br>Nachsorge
Workshop 5	2055-2058	Bei entlassenen Patienten werden offene Einträge oft nicht vervollständigt. Ärzte schauen nicht mehr in die digitale Akte und der Eintrag des Befundes bleibt aus. KI sollte daran erinnern	Herausforderung<br>Dokumentation<br>Interd. Zusammenarbeit<br>Chance<br>Information
Workshop 5	2058-2063	SD wird nicht regelmäßig über Änderungen der OP bei Pat. Informiert. Wünscht sich automatische Information, damit Nachversorgung angepasst werden kann	Herausforderung<br>Dokumentation<br>Interd. Zusammenarbeit<br>Chance<br>Information<br>Nachsorge

Workshop 5	2075-2076	Auftrag ist bei SD noch offen, erscheint für den Arzt aber nicht	Herausforderung<br>Dokumentation<br>Interd. Zusammenarbeit<br>Information
Workshop 5	2135-2037	Sorge, dass durch Einsatz von KI Personalstellen abgebaut werden	Sorge<br>Ethische Bedenken
Workshop 5	2149-2153	Eine Grenze für den Einsatz von KI wird darin gesehen, dass diese im Patientengespräch nicht in dem Maße empathisch und aufmerksam für emotionale Aspekte sein kann, wie es ein Mensch ist	Grenzen<br>Ethische Bedenken<br>Informationen – Defizit in Aufnahme/Wahrnehmung
Workshop 6			
**Interview**	**Zeilen-Nr.**	**Generalisierung**	**Reduktion (auf Kategorie)**
Workshop 6	61	KI ist ein Computerprogramm	Verständnis und Vorerfahrung
Workshop 6	73-74	KI ist ein Hilfsmittel, das Arbeit erleichtern kann	Verständnis und Vorerfahrung
Workshop 6	118-120	KI ist eine Arbeitserleichterung	Verständnis und Vorerfahrung
Workshop 6	125	KI kann helfen und unterstützen	Verständnis und Vorerfahrung
Workshop 6	126-127	KI kann nicht empathisch oder authentisch sein	Verständnis und Vorerfahrung
Workshop 6	146-147	KI kann automatisierte Handlungen durchführen, bspw, Sprache übersetzen	Verständnis und Vorerfahrung
Workshop 6	148	KI kann halluzinieren	Verständnis und Vorerfahrung
Workshop 6	149	KI kann Prozesse vereinfachen und beschleunigen	Verständnis und Vorerfahrung
Workshop 6	150-151	KI kann Emotionen nicht wahrnehmen oder menschliche Kontakte ersetzen	Verständnis und Vorerfahrung

Workshop 6	154	KI kann Arbeitsprozesse erleichtern und verkürzen	Verständnis und Vorerfahrung
Workshop 6	156	KI kann Personal entlasten	Verständnis und Vorerfahrung
Workshop 6	160	KI kann bei der Suche nach Pflegediensten und Heimplätzen unterstützen	Verständnis und Vorerfahrung
Workshop 6	162-163	KI kann helfen, indem es Anrufe und Anmeldungen automatisch vorsortiert	Verständnis und Vorerfahrung
Workshop 6	174-176	Hilfreich wäre, wenn KI erkennt, dass bei einem Patienten Pflegegrad besteht und Angehörige da sind dass diese Fälle automatisch bei der familiären Pflege gemeldet/zugewiesen werden<br><br>Aktuell scheint es so zu sein, dass die zuständigen Bereiche/Personen sich die Fälle selbst aus einem Pool von Anmeldungen heraussuchen müssen	Anwendungsbereiche
Workshop 6	177	KI soll anhand vorliegender Faktoren die zuständige Abteilung erkennen und Patenten dort anmelden	Anwendungsbereiche
Workshop 6	188	TN äußert Bedenken, da KI nicht empathisch sein und Emotionen wahrnehmen kann	Ethische Bedenken<br>Menschliche Kompetenzen
Workshop 6	474-475	Welche Akteure im engeren Kontakt mit dem EM stehen, hängt von der jeweiligen Abteilung ab, für die der Prozess durchgeführt wird.<br>Bsp. Kinder	Akteure und Zuständigkeiten
Workshop 6	488	Mit Rehas wird hier nicht zusammengearbeitet	Interdisziplinäre Zusammenarbeit
Workshop 6	525-527	Patientenaufnahme ist zuständig dafür, Informationen zum Patienten zu erheben und dokumentieren	Verantwortlichkeiten
Workshop 6	529	Wenn die Patientendaten falsch oder unvollständig durch die Patientenaufnahme erhoben wurden, hat TN Probleme beim Prozess	Information und Dokumentation
Workshop 6	533	Patientenaufnahme ist der erste Kontakt für Patienten. Dort wird Karte eingelesen und Information erhoben	Prozessablauf
Workshop 6	552-554	Herausfordernd sind falsch eingetragene Telefonnummern oder Kontaktpersonen, die nicht mehr aktuell sind.	Information und Dokumentation
Workshop 6	565-571	Im Bereich der Reha-Vermittlung kommt es oft vor, dass Formulare vom EM an Ärzte zur Bearbeitung gesendet werden, die dann aber nur teilweise bis gar nicht ausgefüllt oder falsch	Information und Dokumentation<br>Akteure und Zuständigkeiten

		bearbeitet sind. Oft muss auch mehrmals auf die Bearbeitung hingewiesen werden, bis sie vorgenommen wird. Angesprochene Ärzte sind oft nicht in der Zuständigkeit	Interdisziplinäre Zusammenarbeit (Aspekt Kommunikation)
Workshop 6	591-592	Es kann vorkommen, dass die Suche nach einem KZP aus Sicht der Ärzte zu lang dauert und diese den Patienten dann nach Hause entlassen wollen.	
Workshop 6	599-600	Kommunikation mit den Ärzten wird als unzureichend empfunden. Absprachen werden manchmal nicht eingehalten	
Workshop 6	605	TN erhält nicht automatisch Rückmeldung, wenn sich entlassrelevante Aspekte beim Patienten ändern	
Workshop 6	611-615	Wenn ein Übergang eines Patienten vom KH ist die Reha aus Sicht der Ärzte zu lange dauert, wird manchmal eine weitere Anmeldung zur KZP vorgenommen.	
Workshop 6	619-625	Verschiedene Ärzte melden selben Patienten mehrmals und für unterschiedliche Nachsorger an	
Workshop 6	629-631	Wunsch ist, dass KI solche Mehrfachanmeldungen erkennt und Hinweis an Person gibt, die Auftrag anlegen will	
Workshop 6	634-643	Ohne Überleitbogen kann kein Platz beim Pflegedienst oder im Pflegeheim zustande kommen. Dieser muss von der Pflege erstellt werden, das dauert aber sehr lange. Gründe dafür sind, dass in der Akte nicht ausreichend dokumentiert wurde und angesprochene Pflegekräfte den Patienten oft noch nicht kennen	
Workshop 6	644-646	TN müssen zu Beginn des Prozesses wissen, wie pflegeaufwändig die Versorgung des Patienten ist der wird	
Workshop 6	652-659	Herausforderungen im Zusammenhang mit Kostenträgern sind, dass diese schwer erreichbar sind und Anträge, die per Fax gesandt werden, lange Bearbeitungszeiten haben oder verloren gehen	
Workshop 6	669	Fax ist der vorrangige Kommunikationsweg zwischen den Einrichtungen	
Workshop 6	672-680	Kritischer Punkt, der zu Versorgungslücken führt ist, dass Kostenträger (speziell private KK) keine Patientendaten aushändigen. Dadurch werden Patienten ohne Versorgung nach Hause entlassen, weil auch sie die benötigten Informationen nicht (alleine) liefern können	
Workshop 6	717-722	Kontakt mit Angehörigen kann herausfordernd sein, wenn mehrere eines Patienten anrufen und Auskunft oder Beratung wollen, weil die Angehörigen sich untereinander nicht im Austausch befinden	

Workshop 6	771-776	Herausfordernd ist Beschaffung von Hilfsmittel bei Sanitätshäusern. Da nicht jedes Sanitätshaus einen Rahmenvertrag mit jeder KK hat, können nicht alle Hilfsmittel bei einem Sanitätshaus bezogen werden. Das führt dazu, dass ein teil der Hilfsmittel bei einem bestellt werden kann, der Rest aber von einem anderen geliefert werden muss und kostet den SD viel Zeit	
Workshop 6	778-784	Teilweise ist sehr intransparent, welche Hilfsmittel bei den Sanitätshäusern für Angehöriger einer Krankenkasse übernommen werden	
Workshop 6	785-787	Hilfsmittel müssen vor Entlassung gestellt sein, ansonsten kommt es zu Beeinträchtigung des Entlassungsprozess	
Workshop 6	804-811	KI kann dabei unterstützen, die von der jeweiligen KK übernommenen Hilfsmittel beim Sanitätshaus der Wahl anzuzeigen	
Workshop 6	835	Prozess beginnt, wenn Anforderung an SD gestellt wird. Dies geschieht formal durch den Arzt. Er kann aber den Impuls von Angehörigen oder SD erhalten.	
Workshop 6	857-858	Herausforderung bei Prozessbeginn ist, dass Inhalte der Anforderungen unklar, nicht vorhanden oder widersprüchlich sind.	
Workshop 6	876	Problem ist auch, wenn Anmeldung zum EM zu spät erfolgt	
Workshop 6	884-893	Herausforderung zu Prozessbeginn ist, wenn keine direkte Telefonnummer zum Arzt hinterlegt ist, der SD angefordert hat. Oft muss SD bei diesem noch Informationen einholen, weil sie nicht eindeutig sind	
Workshop 6	902-904	Nachsorgebedarf wird bei Anforderung des EM nicht korrekt angegeben.	
Workshop 6	909-910	Lösungsansatz für eine fälschliche Mehrauswahl an Nachsorgebedarf kann durch KI gegeben werden	
Workshop 6	933	Mangelnde Einarbeitung der Ärzte, insbesondere der Assistenzärzte ist ein Problem	
Workshop 6	954-956	Zu Beginn muss unbedingt erhoben werden, ob eine Versorgung bereits besteht und ob Bedarf an Anpassung besteht	
Workshop 6	969	Prozessschritt: Mitarbeiter nimmt sich des Falls an und druckt ihn aus	
Workshop 6	994-995	SD nehmen ausgedruckte Fälle, weil sie beim Patienten vor Ort dokumentieren müssen und die bestehenden Informationen vor Ort brauchen. Tablets gibt es nicht	

Workshop 6	1018-1019	Nächster Prozessschritt ist direkter Kontakt zu Patient	
Workshop 6	1030-1034	Bei dieser Gruppe von TN ist das Auffinden der Patienten weniger ein Problem. Schwierig ist es u.U. den Patienten zum richtigen Zeitpunkt anzutreffen, da SD keine Einsicht in seine Termine hat	
Workshop 6	1047	Herausforderung liegt darin, dass manche Termine spontan frei werden und Patient ohne Vorankündigung zur Untersuchung geht	
Workshop 6	1076-1078	SD macht mit Angehörigen Termin aus, wenn diese in den Prozess involviert werden müssen	
Workshop 6	1092-1094	Nächster Prozessschritt ist das Ermitteln des Bedarfes. Das erfolgt oft auch gemeinsam mit Angehörigen	
Workshop 6	1110-1113	Zur Ermittlung des Bedarfs gehört, je nach Abteilung, auch die Rücksprache mit den behandelnden Ärzten. Im Idealfall sind diese Informationen schon mit in der Anforderung hinterlegt	
Workshop 6	1122-1124	Vor dem Gespräch ist noch nicht bekannt, ob eine Anschlussbehandlung/Nachsorge benötigt oder gewünscht ist. Hilfreich wäre, wenn SD beim Patientenkontakt vor Ort spontan Zugriff auf Anträge hätte.	
Workshop 6	1153-1163	Bedarfserhebung umfasst auch Rücksprache mit anderen Disziplinen	
Workshop 6	1179-1183	Bei manchen Patienten kann die Bedarfserhebung mehrere Termine benötigen	
Workshop 6	1199-1203	Für die Anträge der Reha werden Befunde benötigt	
Workshop 6	1204-1209	SD muss oft um Erstellung der ärztlichen Befunde bitten	
Workshop 6	1222-1224	KI könnte automatisch erkennen, dass ärztlicher Befund noch offen ist und eine Erinnerung an den Arzt versenden	
Workshop 6	1376-1384	Wenn Bedarf erhoben wurde und Rücksprache mit anderen Disziplinen erfolgt ist, ist die Entlassplanung der nächste Schritt. Das inkludiert auch das Stellen der Anträge	
Workshop 6	1428-1436	Nächster Schritt ist Information des Patienten, des Arztes und der Station	
Workshop 6	1441-1448	Bei manchen Patienten kann Entlassung erst erfolgen, wen Hilfsmittel vorhanden sind	

Workshop 6	1471-1472	Anforderung an KI ist, dass nicht mehrere Bedarfe der Nachsorge gleichzeitig ausgewählt werden können	
Workshop 6	1755-1756	TN äußern Bedenken, dass sensible Daten in die falschen Hände geraten könnten	
Workshop 6	1760-1764	Außenwirkung des UKB könnte durch den Einsatz von KI negativ beeinflusst werden	
Workshop 6	1810-1811	Es besteht die Möglichkeit, dass die KI nicht empathisch gegenüber den Nutzenden ist	
Workshop 6	1812-1813	Eine Sorge besteht darin, dass Fälle falsch eingeschätzt werden und bspw. Nebendiagnosen nicht angemessen berücksichtigt werden	
Workshop 6	1827-1828	KI könnte Mitarbeitende dazu verleiten, am Prozess eher passiv teilzunehmen und ihn nicht mehr ausreichend zu reflektieren	
Workshop 6	1855-1856	Weitere ethische Bedenken sind, dass durch den Einsatz von KI das Personal reduziert wird oder die Löhne gesenkt werden	
Workshop 6	1867-1868	Sorge, dass durch fehlerhafte Eingaben der Prozess in eine falsche Richtung läuft und infolge dessen die Verweildauer verlängert wird	
