# Supplementary material for: Optimizing Hospital Discharge Planning: Empirical Insights and Requirements of AI-Based Technologies From an Explorative Mixed Methods Field Study
Source: JMIR Form Res. 2026 Mar 24;10:e81824. doi: 10.2196/81824 (PMC13012232; doi:10.2196/81824)
Supplement: Multimedia Appendix 8 [file formative-v10-e81824-s008.pdf]

# Übersicht generalisierte Aussagen

## Inhalt


Oberkategorie Künstliche Intelligenz ........ 2
Kategorie Erwartungen an KI ........ 2
Kategorie Grenzen des Einsatzes ........ 6
Unterkategorie Technische Grenzen ........ 6
Unterkategorie Ethische und rechtliche Bedenken ........ 7
Unterkategorie Organisatorische Hürden ........ 8
Unterkategorie Menschliche Kompetenzen ........ 9
Kategorie Verständnis und Vorerfahrung ........ 10
Kategorie Einstellung ........ 11
Oberkategorie Entlassmanagement ........ 14
Kategorie Prozessablauf ........ 14
Unterkategorie Schritte und Phasen ........ 14
Unterkategorie Zeitliche Abläufe ........ 20
Kategorie Akteure und Zuständigkeiten ........ 21
Unterkategorie Beteiligte Rollen ........ 21
Unterkategorie Verantwortlichkeiten ........ 27
Unterkategorie Interdisziplinäre Zusammenarbeit ........ 31
Kategorie Patientenzentrierte Versorgungsbedarfe ........ 40
Unterkategorie Medizinische und pflegerische Bedarfe ........ 40
Unterkategorie Gesundheitskompetenz und Beratungsbedarf ........ 43
Kategorie Herausforderungen und Verbesserungspotentiale ........ 47
Unterkategorie Ressourcenbedarf ........ 47
Unterkategorie Strukturelle Herausforderungen ........ 47
Unterkategorie Verbesserungspotentiale ........ 49
Unterkategorie Herausforderungen und Barrieren ........ 51

# Oberkategorie Künstliche Intelligenz

## Kategorie Erwartungen an KI

### Aussagen nach Workshops

#### Workshop 1

- Unterstützung bei Sortierung von Aufgaben und Telefonanrufen
- Klärung und Anzeige von Zuständigkeiten
- Automatisierung von Prozessen
- Vorschläge für passende Nachversorger:innen
- Erfassung und Prüfung relevanter Daten bei Anmeldung von Patient:innen zum Entlassmanagement
- Automatische Benachrichtigung bei Verlegungen, Todesfällen oder Prozessänderungen

#### Workshop 2

- Vollständigkeit der Eingabefelder sicherstellen
- Automatische Dokumentation und Systemeinträge
- Vorgegebene Textbausteine
- Mehr Zeit für Patient:innen und Angehörige
- Schnellere Dokumentation und Gesamtprozess
- Automatische Erstellung klinischer Aufträge bei Bedarf
- Bürokratieabbau
- Sprachnachrichten als Text im E-Mail-Postfach
- Klinischer Auftrag nur bei vollständigen Daten und Notwendigkeit
- Aufforderung des Systemnutzenden zur Auftragserstellung bei definierten Kriterien
- Barthel-Index verpflichtend eintragen
- Benachrichtigung bei Änderung des Barthel-Index
- Benachrichtigung bei Operationen
- Individuelle Benachrichtigungseinstellungen
- Chatbot zur Ermittlung von Kostenträgern
- Mobile Endgeräte zur Dokumentation
- Automatische Ermittlung des Kostenträgers
- Patient:innen automatisch über Termine informieren
- Benachrichtigung bei fehlender Kostenzusage vor Reha-Antritt
- Hinweis bei Personen unter 65 Jahren, die für eine Pflegeeinrichtung angemeldet werden sollen
- Automatischer Versand von Befunden und Verordnungen an Akteure

#### Workshop 3

- Benachrichtigung bei OP-Verschiebung oder -Ausfall
- Zentrale Übersicht aller entlassrelevanten Informationen
- Automatische Ermittlung und Anzeige freier Nachsorgekapazitäten
- Erinnerungsfunktion für Termine und Aufgaben
- Angehörige automatisch informieren

- Weiterleitung von Anträgen/Dokumenten nach Kontakt mit Angehörigen
- Patientenakte nur bei hinterlegtem Barthel-Index nutzbar
- Allgemeine Unterstützung durch KI
- Erhebung von Informationen zu Verwahrlosung und Suchtproblematik (Reha-Ausschlusskriterium)
- Zwingende Abfrage zu möglicher Isolierpflicht
- Plausibilitätsprüfung der Daten im Entlassungsbericht
- Vorschlag des passenden Nachversorgers anhand Pflegeinformationen
- Anpassung der Nachsorgeoptionen basierend auf Patientendaten
- Plausibilitätsprüfung bei Einträgen
- Erkennung und Hervorhebung von Kommunikationsbarrieren bei Patient:innen (eingeschränktes Hörvermögen, kognitive Beeinträchtigung etc.)
- Automatische Erinnerung bei fehlenden Angaben zu Sinneswahrnehmungen und kognitivem Status
- Benachrichtigung bei Verlegung von Patient:innen

**Workshop 4**

- Anrufer nach Anliegen vorsortieren
- Automatische Ermittlung und Anzeige freier Nachsorgekapazitäten
- Automatische Erstellung des Barthel-Index bei Anlage eines Auftrags für Entlassmanagement
- Benachrichtigung bei Veränderungen bei Patient:innen
- Automatisches Ausfüllen von Anträgen**/**Vorausfüllen von Anträgen und Formularen mit vorhandenen Patientendaten
- Frühzeitige Ermittlung des Bedarfs an unterstützenden Leistungen bei geplanten chirurgischen Eingriffen
- Übernahme bereits dokumentierter Patientendaten bei erneutem Aufenthalt
- Patient:innen durch den automatisierten Versand von Erstinformationen beruhigen

**Workshop 5**

- Prozesse zusammenführen, verbessern und erleichtern
- Schnelle Texterstellung und -auswertung
- Unterstützung bei Personalmangel
- Autonome Planung des Entlassmanagements und der Nachsorge
- Automatisierte Ermittlung geeigneter Nachversorger und Kontaktaufnahme zu Kostenträgern
- Bereitstellung der bereits im persönlichen Kontakt besprochenen Beratungsthemen für Patient:innen und Angehörige
- Automatische Hinweise für Patient:innen zur Einleitung des Entlassmanagements
- Bedarfsermittlung und Informationsweitergabe per Mail
- Benachrichtigung bei Patientenverlegungen und OP-Änderungen
- Vermeidung von doppelten Anforderungen an den Sozialdienst
- Automatische Prüfung anhand definierter Kriterien, ob als dringlich markierte Fälle tatsächlich notwendig sind
- Unterstützung bei Bedarfsermittlung für Hilfsmittel
- Vorbereitung relevanter Unterlagen und Gesprächsschemata für Beratungsgespräche

- Fragenkatalog anhand von Schlüsselwörtern in der ePA generieren und sich Patient:innendaten aus System ziehen, um entsprechende Anträge vorzubereiten
- Automatische Prozessabläufe basierend auf spezifischen Patient:inneninformationen
- Automatische Aktualisierung von Dokumentenanforderungen

**Workshop 6**

- Arbeitsprozesse erleichtern und verkürzen
- Personal entlasten
- Unterstützung bei Suche nach Pflegediensten und Heimplätzen
- Automatische Vorsortierung von Anrufen und Anmeldungen
- Zuständige Abteilung anhand vorliegender Faktoren erkennen und Patient:innen anmelden
- Erkennung und Verhinderung von Mehrfachanmeldungen
- Anzeigen von durch die Krankenkasse übernommene Hilfsmittel beim jeweiligen Sanitätshaus
- Unterbindung von Mehrauswahl von Nachsorgebedarf bei der Anmeldung eines Auftrags
- Automatische Erinnerung an Ärzt:innen bei offenen Befunden

## Gesamtdarstellung aller Punkte

- Unterstützung bei Sortierung von Aufgaben und Telefonanrufen
- Klärung und Anzeige von Zuständigkeiten
- Automatisierung von Prozessen
- Vorschläge für passende Nachversorger:innen
- Erfassung und Prüfung relevanter Daten bei Anmeldung von Patient:innen zum Entlassmanagement
- Automatische Benachrichtigung bei Verlegungen, Todesfällen oder Prozessänderungen
- Vollständigkeit der Eingabefelder sicherstellen
- Automatische Dokumentation und Systemeinträge
- Vorgegebene Textbausteine
- Mehr Zeit für Patient:innen und Angehörige
- Schnellere Dokumentation und Gesamtprozess
- Automatische Erstellung klinischer Aufträge bei Bedarf
- Bürokratieabbau
- Sprachnachrichten als Text im E-Mail-Postfach
- Klinischer Auftrag nur bei vollständigen Daten und Notwendigkeit
- Aufforderung des Systemnutzenden zur Auftragserstellung bei definierten Kriterien
- Barthel-Index verpflichtend eintragen
- Benachrichtigung bei Änderung des Barthel-Index
- Benachrichtigung bei Operationen
- Individuelle Benachrichtigungseinstellungen
- Chatbot zur Ermittlung von Kostenträgern
- Mobile Endgeräte zur Dokumentation
- Automatische Ermittlung des Kostenträgers
- Patient:innen automatisch über Termine informieren

- Benachrichtigung bei fehlender Kostenzusage vor Reha-Antritt
- Hinweis bei Personen unter 65 Jahren, die für eine Pflegeeinrichtung angemeldet werden sollen
- Automatischer Versand von Befunden und Verordnungen an Akteure
- Benachrichtigung bei OP-Verschiebung oder -Ausfall
- Zentrale Übersicht aller entlassrelevanten Informationen
- Automatische Ermittlung und Anzeige freier Nachsorgekapazitäten
- Erinnerungsfunktion für Termine und Aufgaben
- Angehörige automatisch informieren
- Weiterleitung von Anträgen/Dokumenten nach Kontakt mit Angehörigen
- Patientenakte nur bei hinterlegtem Barthel-Index nutzbar

- Allgemeine Unterstützung durch KI
- Erhebung von Informationen zu Verwahrlosung und Suchtproblematik (Reha-Ausschlusskriterium)
- Zwingende Abfrage zu möglicher Isolierpflicht
- Plausibilitätsprüfung der Daten im Entlassungsbericht
- Vorschlag des passenden Nachversorgers anhand Pflegeinformationen
- Anpassung der Nachsorgeoptionen basierend auf Patientendaten
- Plausibilitätsprüfung bei Einträgen
- Erkennung und Hervorhebung von Kommunikationsbarrieren bei Patient:innen (eingeschränktes Hörvermögen, kognitive Beeinträchtigung etc.)
- Automatische Erinnerung bei fehlenden Angaben zu Sinneswahrnehmungen und kognitivem Status
- Benachrichtigung bei Verlegung von Patient:innen
- Anrufer nach Anliegen vorsortieren
- Automatische Ermittlung und Anzeige freier Nachsorgekapazitäten
- Automatische Erstellung des Barthel-Index bei Anlage eines Auftrags für Entlassmanagement
- Benachrichtigung bei Veränderungen bei Patient:innen
- Automatisches Ausfüllen von Anträgen/Vorausfüllen von Anträgen und Formularen mit vorhandenen Patientendaten
- Frühzeitige Ermittlung des Bedarfs an unterstützenden Leistungen bei geplanten chirurgischen Eingriffen
- Übernahme bereits dokumentierter Patientendaten bei erneutem Aufenthalt
- Patient:innen durch den automatisierten Versand von Erstinformationen beruhigen
- Prozesse zusammenführen, verbessern und erleichtern
- Schnelle Texterstellung und -auswertung
- Unterstützung bei Personalmangel
- Autonome Planung des Entlassmanagements und der Nachsorge
- Automatisierte Ermittlung geeigneter Nachversorger und Kontaktaufnahme zu Kostenträgern
- Bereitstellung der bereits im persönlichen Kontakt besprochenen Beratungsthemen für Patient:innen und Angehörige
- Automatische Hinweise für Patient:innen zur Einleitung des Entlassmanagements
- Bedarfsermittlung und Informationsweitergabe per Mail
- Benachrichtigung bei Patientenverlegungen und OP-Änderungen
- Vermeidung von doppelten Anforderungen an den Sozialdienst

- Automatische Prüfung anhand definierter Kriterien, ob als dringlich markierte Fälle tatsächlich notwendig sind
- Unterstützung bei Bedarfsermittlung für Hilfsmittel
- Vorbereitung relevanter Unterlagen und Gesprächsschemata für Beratungsgespräche
- Fragenkatalog anhand von Schlüsselwörtern in der ePA generieren und sich Patient:innendaten aus System ziehen, um entsprechende Anträge vorzubereiten
- Automatische Prozessabläufe basierend auf spezifischen Patient:inneninformationen
- Automatische Aktualisierung von Dokumentenanforderungen
- Personal entlasten
- Unterstützung bei Suche nach Pflegediensten und Heimplätzen
- Automatische Vorsortierung von Anrufen und Anmeldungen
- Zuständige Abteilung anhand vorliegender Faktoren erkennen und Patient:innen anmelden
- Erkennung und Verhinderung von Mehrfachanmeldungen
- Anzeigen von durch die Krankenkasse übernommene Hilfsmittel beim jeweiligen Sanitätshaus
- Unterbindung von Mehrauswahl von Nachsorgebedarf bei der Anmeldung eines Auftrags
- Automatische Erinnerung an Ärzt:innen bei offenen Befunden

## Kategorie Grenzen des Einsatzes

### Unterkategorie Technische Grenzen

#### Aussagen nach Workshops

**Workshop 1**

- ChatGPT hat keine aktuelle Datenbank
- ChatGPT gibt auch falsche Antworten aus

**Workshop 3**

- Teilnehmende äußert sich besorgt, wie die KI im Falle einer personellen Unterbesetzung mit terminierten Aufgaben umgehen kann. Das Bedenken ist, dass die zu bearbeitenden Tätigkeiten durch die Personalsituation nicht entsprechend der Planung ausgeführt werden können. Daran schließt sich die Frage an, wie die KI mit einem solchem Umstand umgehen könnte, ob sie bspw. automatisch Schritte einleitet, um Deadlines entsprechend anzupassen

**Workshop 5:**

- Grenzen von KI liegen auch in ihrem Training

**Workshop 6:**

- Eine Sorge besteht darin, dass Fälle falsch eingeschätzt werden und bspw. Nebendiagnosen nicht angemessen berücksichtigt werden
- Sorge, dass durch fehlerhafte Eingaben der Prozess in eine falsche Richtung läuft und infolge dessen die Verweildauer verlängert wird

#### Gesamtdarstellung aller Punkte zur Unterkategorie

- ChatGPT hat keine aktuelle Datenbank
- ChatGPT gibt auch falsche Antworten aus
- Teilnehmende äußert sich besorgt, wie die KI im Falle einer personellen Unterbesetzung mit terminierten Aufgaben umgehen kann. Das Bedenken ist, dass die zu bearbeitenden Tätigkeiten durch die Personalsituation nicht entsprechend der Planung ausgeführt werden können. Daran schließt sich die Frage an, wie die KI mit einem solchem Umstand umgehen könnte, ob sie bspw. automatisch Schritte einleitet, um Deadlines entsprechend anzupassen
- Grenzen von KI liegen auch in ihrem Training
- Eine Sorge besteht darin, dass Fälle falsch eingeschätzt werden und bspw. Nebendiagnosen nicht angemessen berücksichtigt werden
- Sorge, dass durch fehlerhafte Eingaben der Prozess in eine falsche Richtung läuft und infolge dessen die Verweildauer verlängert wird

## Unterkategorie Ethische und rechtliche Bedenken

### Aussagen nach Workshops

**Workshop 1**

- Annahme, dass KI durch unreflektierte Nutzung unethische Inhalte lernt
- Teilnehmende war von ethischem Aspekt des Lernens der KI überrascht

**Workshop 2**

- Bedenken in Bezug des Aspekts Datenschutz – ob Patienten und deren Daten sicher sind, ob Hacker leicht Zugriff haben können
- Sorge, dass höhere Arbeitsbelastung des SD infolge der Automatisierung von Teilbereichen eintreten könnte
- Sorge, dass Stellen aufgrund der KI nicht (nach-)besetzt werden, da Arbeitslast vermeintlich verringert wird

**Workshop 3**

- Frage der Teilnehmende, wer in der Verantwortung steht, wenn in Folge einer Fehlentscheidung durch KI Schäden entstehen

**Workshop 4**

- Teilnehmende erkundigt sich nach ethischen Aspekten beim Training der KI
- Teilnehmende äußert Bedenken zur Datensicherheit der KI

**Workshop 5:**

- Sorge, wie fehlerhafte Trainingsdaten sich auswirken
- Sorge, dass durch Einsatz von KI Personalstellen abgebaut werden
- Sieht Herausforderung darin, Menschen nicht zu ersetzen. KI könne nicht so professionell handeln wie ein Mensch. Sorge, dass Einsatz der KI als Argument für Personaleinsparung genutzt wird

**Workshop 6:**

- Teilnehmende äußern Bedenken, dass sensible Daten in die falschen Hände geraten könnten
- KI könnte Mitarbeitende dazu verleiten, am Prozess eher passiv teilzunehmen und ihn nicht mehr ausreichend zu reflektieren

- Weitere ethische Bedenken sind, dass durch den Einsatz von KI das Personal reduziert oder die Löhne gesenkt würden

### Gesamtdarstellung aller Punkte zur Unterkategorie

- Annahme, dass KI durch unreflektierte Nutzung unethische Inhalte lernt
- Teilnehmende war von ethischem Aspekt des Lernens der KI überrascht
- Bedenken in Bezug des Aspekts Datenschutz – ob Patienten und deren Daten sicher sind, ob Hacker leicht Zugriff haben können
- Sorge, dass höhere Arbeitsbelastung des SD infolge der Automatisierung von Teilbereichen eintreten könnte
- Sorge, dass Stellen aufgrund der KI nicht (nach-)besetzt werden, da Arbeitslast vermeintlich verringert wird
- Frage der Teilnehmende, wer in der Verantwortung steht, wenn in Folge einer Fehlentscheidung durch KI Schäden entstehen
- Sorge, wie fehlerhafte Trainingsdaten sich auf die Qualität der Versorgung auswirken
- Sieht Herausforderung darin, Menschen nicht zu ersetzen. KI könne nicht so professionell handeln wie ein Mensch. Sorge, dass Einsatz der KI als Argument für Personaleinsparung genutzt wird
- KI könnte Mitarbeitende dazu verleiten, am Prozess eher passiv teilzunehmen und ihn nicht mehr ausreichend zu reflektieren
- Bedenken, dass die KI als Argument für Personaleinsparungen oder -abbau genutzt werden könnte
- Der Einsatz von KI könnte zur Reduzierung von Personal oder zur Senkung der Löhne führen
- Herausforderung, Menschen nicht zu ersetzen: KI könne nicht so professionell handeln wie ein Mensch

## Unterkategorie Organisatorische Hürden

### Aussagen nach Workshops

#### Workshop 1

- Kritische Auseinandersetzung der Teilnehmerin in Bezug auf Anwendung in unterschiedlichen klinischen Standorten

#### Workshop 2

- Sieht grundlegenden Klärungsbedarf innerhalb der Organisation, bevor neue Maßnahmen geplant werden können, andernfalls würde Implementierung von KI nicht erfolgreich sein können

#### Workshop 6:

- Außenwirkung des UKB könnte durch den Einsatz von KI negativ beeinflusst werden

### Gesamtdarstellung aller Punkte zur Unterkategorie

- Kritische Auseinandersetzung der Teilnehmerin in Bezug auf Anwendung in unterschiedlichen klinischen Standorten

- Sieht grundlegenden Klärungsbedarf innerhalb der Organisation, bevor neue Maßnahmen geplant werden können, andernfalls würde Implementierung von KI nicht erfolgreich sein können
- Außenwirkung des UKB könnte durch den Einsatz von KI negativ beeinflusst werden

## Unterkategorie Menschliche Kompetenzen

### Aussagen nach Workshops

**Workshop 2**

- Sorge, ob automatisierte Anrufweiterleitung emotional aufgeladen Patient:innen Gerecht werden kann
- Schilderung einer sehr emotionalen Frau, die wegen ihrer Aufregung die Fragen des SD nur schwer beantworten konnte. Vermutung, dass diese Angehörige durch Assistenzsystem per Telefon nicht abgeholt werden könne
- Teilnehmende differenziert zwischen verschiedenen Arten von Tätigkeiten, die ihrer Meinung nach für den Einsatz von KI geeignet sind
- KI und deren generierte Text kann emotionale Bedürfnisse nicht befriedigen

**Workshop 4**

- Einschätzung zur Erreichbarkeit der Zielgruppe durch KI ist eher mäßig
- Auch, wenn bei Klientel digitale Kompetenzen vorliegen/durch Mitarbeitende vermutet werden, hat die emotionale Belastung infolge der Erkrankung großen Einfluss auf das Ausmaß der Eigeninitiative zur Planung der Nachsorge
- Teilnehmende äußert Bedenken, dass Patienten und Angehörige den Einsatz von KI im EM schwer bis nicht annehmen, weil sie menschlichen Austausch bevorzugen

**Workshop 5:**

- KI kann Gesamtsituation nicht so wahrnehmen und deuten, wie ein Mensch
- Teilnehmende sieht unüberwindbare Grenze der Einsatzmöglichkeit von KI in Wahrnehmung/Erfassung komplexer Situationen

**Workshop 6:**

- Teilnehmende äußert Bedenken, da KI nicht empathisch sein und Emotionen wahrnehmen kann

### Gesamtdarstellung aller Punkte zur Unterkategorie

- Sorge, ob automatisierte Anrufweiterleitung emotional aufgeladen Patient:innen gerecht werden kann
- Teilnehmende differenziert zwischen verschiedenen Arten von Tätigkeiten, die ihrer Meinung nach für den Einsatz von KI geeignet sind
- KI und deren generierte Text kann emotionale Bedürfnisse nicht befriedigen
- Einschätzung zur Erreichbarkeit der Zielgruppe durch KI ist eher mäßig
- Auch, wenn bei Klientel digitale Kompetenzen vorliegen/durch Mitarbeitende vermutet werden, hat die emotionale Belastung infolge der Erkrankung großen Einfluss auf das Ausmaß der Eigeninitiative zur Planung der Nachsorge
- KI kann Gesamtsituation nicht so wahrnehmen und deuten, wie ein Mensch
- Teilnehmende sieht unüberwindbare Grenze der Einsatzmöglichkeit von KI in Wahrnehmung/Erfassung komplexer Situationen

- Teilnehmende äußert Bedenken, da KI nicht empathisch sein und Emotionen wahrnehmen kann

## Kategorie Verständnis und Vorerfahrung

### Aussagen nach Workshops

#### Workshop 1

- KI wirkt durch Algorithmus, ist lernfähig
- KI hat festen Aufgabenbereich
- Wesentliche Charakteristika von KI sind bekannt
- Teilnehmer hat ChatGPT in Uni genutzt
- Findet ChatGPT spannend

#### Workshop 2

- Teilnehmende hat Vorerfahrung mit ChatGPT. Nutzt es privat und beruflich. Wendet es als Unterstützung zur Formulierung von Botschaften. Ist sehr begeistert davon
- Grenzen der Pflegerobotik sind Teilnehmende bekannt

#### Workshop 3

- KI ist künstliche Intelligenz, die Arbeitsalltag erleichtert
- KI kann nach Priorität selektieren
- Kennt LLM durch ihren Sohn

#### Workshop 4

- Teilnehmende kennen Audiogenererierungen durch KI
- Teilnehmende hat wenig Vorerfahrung mit KI
- Keine Auseinandersetzung mit dem Thema KI zuvor
- Teilnehmende nimmt an, dass KI auf Informationseingabe durch Menschen angewiesen ist
- Teilnehmende ist überrascht, dass KI die Fähigkeit besitzt, Entscheidungen zu treffen

#### Workshop 5:

- Hat ChatGPT bereits genutzt
- Algorithmus, der ein neuronales Netz simuliert
- Programm, das Arbeit abnehmen und Texte schreiben soll
- Teilnehmende hat im Rahmen einer Hausaufgabe für ihr Kind ChatGPT genutzt
- Ohne Trainingsdaten keine KI möglich, Art und Umfang des Trainings begrenzen Können
- Künstliche Unterstützung, Spracherstellung
- Teilnehmende möchte wissen, ob KI menschliche Emotionen und Wahrnehmung ersetzen kann
- Fragt sich, ob KI, wie ein Mensch auch, die persönlichen Eindrücke zum Patienten wahrnehmen kann

#### Workshop 6:

- KI ist ein Computerprogramm
- KI ist ein Hilfsmittel, das Arbeit erleichtern kann
- KI ist eine Arbeitserleichterung

- KI kann helfen und unterstützen
- KI kann nicht empathisch oder authentisch sein
- KI kann automatisierte Handlungen durchführen, bspw, Sprache übersetzen
- KI kann halluzinieren
- KI kann Prozesse vereinfachen und beschleunigen

### Gesamtdarstellung aller Punkte

- KI wirkt durch Algorithmus, ist lernfähig
- KI hat festen Aufgabenbereich
- Wesentliche Charakteristika von KI sind bekannt
- Teilnehmer hat ChatGPT in Uni genutzt
- Findet ChatGPT spannend
- Teilnehmende hat Vorerfahrung mit ChatGPT. Nutzt es privat und beruflich. Wendet es als Unterstützung zur Formulierung von Botschaften. Ist sehr begeistert davon
- Grenzen der Pflegerobotik sind Teilnehmende bekannt
- KI ist künstliche Intelligenz, die Arbeitsalltag erleichtert
- KI kann nach Priorität selektieren
- Kennt LLM durch ihren Sohn
- Teilnehmende kennen Audiogenererierungen durch KI
- Teilnehmende hat wenig Vorerfahrung mit KI
- Keine Auseinandersetzung mit dem Thema KI zuvor
- Teilnehmende nimmt an, dass KI auf Informationseingabe durch Menschen angewiesen ist
- Teilnehmende ist überrascht, dass KI die Fähigkeit besitzt, Entscheidungen zu treffen
- Hat ChatGPT bereits genutzt
- Algorithmus, der ein neuronales Netz simuliert
- Programm, das Arbeit abnehmen und Texte schreiben soll
- Teilnehmende hat im Rahmen einer Hausaufgabe für ihr Kind ChatGPT genutzt
- Ohne Trainingsdaten keine KI möglich, Art und Umfang des Trainings begrenzen Können
- Künstliche Unterstützung, Spracherstellung
- Teilnehmende möchte wissen, ob KI menschliche Emotionen und Wahrnehmung ersetzen kann
- Fragt sich, ob KI, wie ein Mensch auch, die persönlichen Eindrücke zum Patienten wahrnehmen kann
- KI ist ein Computerprogramm
- KI ist ein Hilfsmittel, das Arbeit erleichtern kann
- KI ist eine Arbeitserleichterung
- KI kann nicht empathisch oder authentisch sein
- KI kann automatisierte Handlungen durchführen, bspw, Sprache übersetzen
- KI kann halluzinieren
- KI kann Prozesse vereinfachen und beschleunigen

## Kategorie Einstellung

### Aussagen nach Workshops

#### Workshop 1

- KI hat maschinellen Sprachstil
- Findet KI interessant
- Nutzt ChatGPT zum Finden von Rezepten im Alltag
- Findet ChatGPT spannend

**Workshop 2**

- Nutzt ChatGPT als Inspiration für das Verfassen eigener Texte in Beratungsgesprächen
- ist interessiert an KI
- Ist interessiert daran, wie KI im Entlassmanagement eingesetzt werden kann
- Nach Ansicht der Teilnehmenden soll KI langfristig Dinge wie Berufsgruppen ersetzen
- Interesse an Funktionsweise
- Interessiert an Funktionsweise
- Findet Lernfähigkeit der KI beängstigend
- KI ist unvermeidbares Phänomen, dass jeden einholt
- Empfindet hohem Papierverbrauch als unangenehm, ist daher an digitalen Lösungen interessiert
- Einstellung zu Neuem ist, dass am Anfang alles herausfordernd ist. Wenn man sich mit dem Gegenstand aber auseinandersetzt, kann man lernen

**Workshop 3**

- Teilnehmende finden partizipativen Gestaltungsprozess gut
- Teilnehmende würde sich nie gänzlich auf KI verlassen
- Teilnehmende ist interessiert an technischen Anwendungen, lernt schnell.

**Workshop 4**

- Jüngere Patienten haben ihr Smartphone immer bei sich. Teilnehmende bindet sie dann damit in das Entlassmanagement ein
- Teilnehmende sieht in KI auch ein Risikopotential
- Teilnehmende möchten sich eigentlich nicht mit KI oder damit verbundenen Veränderung auseinandersetzen
- Teilnehmende zieht sich beim Thema Digitalisierung zurück

**Workshop 5:**

- KI wird durch Mensch entwickelt, kann sich eigenständig weiterentwickeln
- Künstliche Unterstützung, Spracherstellung
- KI als Teilersatz des menschlichen Gehirns
- Teilnehmende möchte wissen, ob KI menschliche Emotionen und Wahrnehmung ersetzen kann
- Fragt sich, ob KI, wie ein Mensch auch, die persönlichen Eindrücke zum Patienten wahrnehmen kann
- KI kann gezielt Informationen rausfiltern
- Ist interessiert an Funktionsweise von KI

## Gesamtdarstellung aller Punkte

- KI hat maschinellen Sprachstil
- Nutzt ChatGPT zum Finden von Rezepten im Alltag
- Findet ChatGPT spannend
- Nutzt ChatGPT als Inspiration für das Verfassen eigener Texte in Beratungsgesprächen

- Ist interessiert daran, wie KI im Entlassmanagement eingesetzt werden kann
- Nach Ansicht der Teilnehmenden soll KI langfristig Dinge wie Berufsgruppen ersetzen
- Findet Lernfähigkeit der KI beängstigend
- KI ist unvermeidbares Phänomen, dass jeden einholt
- Empfindet hohem Papierverbrauch als unangenehm, ist daher an digitalen Lösungen interessiert
- Einstellung zu Neuem ist, dass am Anfang alles herausfordernd ist. Wenn man sich mit dem Gegenstand aber auseinandersetzt, kann man lernen
- Teilnehmende finden partizipativen Gestaltungsprozess gut
- Teilnehmende würde sich nie gänzlich auf KI verlassen
- Teilnehmende ist interessiert an technischen Anwendungen, lernt schnell.
- Jüngere Patienten haben ihr Smartphone immer bei sich. Teilnehmende bindet sie dann damit in das Entlassmanagement ein
- Teilnehmende sieht in KI auch ein Risikopotential
- Teilnehmende möchten sich eigentlich nicht mit KI oder damit verbundenen Veränderung auseinandersetzen
- Teilnehmende zieht sich beim Thema Digitalisierung zurück
- KI wird durch Mensch entwickelt, kann sich eigenständig weiterentwickeln
- Künstliche Unterstützung, Spracherstellung
- KI als Teilersatz des menschlichen Gehirns
- Teilnehmende möchte wissen, ob KI menschliche Emotionen und Wahrnehmung ersetzen kann
- Fragt sich, ob KI, wie ein Mensch auch, die persönlichen Eindrücke zum Patienten wahrnehmen kann
- KI kann gezielt Informationen rausfiltern
- Ist interessiert an Funktionsweise von KI

# Oberkategorie Entlassmanagement

## Kategorie Prozessablauf

### Unterkategorie Schritte und Phasen

**Aussagen nach Workshops**

**Workshop 1**

Anmeldung zu EM

- EM wird bei SD angemeldet
- Regelmäßig Beratung/Aufklärung aufgrund falsch geplanter Nachversorgung
- Es gibt eine Prozessdefinition, diese Prozessbeschreibung wird nicht regulär eingehalten
- Es gib keinen Standard. Ablauf wird mündlich weitergegeben und muss gelernt werden
- Vorgehen beim EM begründet sich durch Expertise, Erfahrung des TN
- Klinischer Auftrag soll bei Aufnahme, spätestens binnen 24h, erfasst und an SD weitergeleitet werden. Daran schließt Bedarfserhebung an
- Patient gilt als zum EM aufgenommen, wenn er versorgt werden möchte, allen Dokumenten zugestimmt hat und SD einen klinischen Auftrag erhält. Nicht jeder Patient hat Wunsch nach EM
- Klinischer Auftrag kann kommen von Station, Arzt oder Pflege, Patient direkt oder Bevollmächtigten. Nur die drei sind berechtigt. Ist erster Prozessschritt im EM.
- Patient muss im System angelegt und aufgeklärt sein, damit EM tätig werden kann
- Zu Beginn des Prozesses erfolgt persönliche Kontaktaufnahme mit Patient oder Angehörigen, dieser Erstkontakt dient der Bedarfserhebung
- Verweildauer ist abhängig von Erkrankung
- Verweildauer mancher Patienten teilweise Jahre. Kommen ein bis zwei Mal pro Monat. Bei ihnen wir Zeit abgekappt.
- Ist Pflegebedarf erhoben, wird persönlich mit Pat./Angehörigen gesprochen und häusliches/pflegerisches Setting erfragt
- Ein etwaiger Pflegegrad des Patienten soll bei Aufnahme hinterlegt werden
- EM nimmt Kontakt zu Ärzten und Pflege auf um Entlasstag zu erfragen und Ersteinschätzung zu erhalten. Barthel-Index ist sehr wichtig
- Bei planbaren Eingriffen kontaktiert SD Patienten im Vorfeld und klärt Antragstellung ab
- Tatsächlicher Hilfebedarf einer Person fällt insbesondere bei Patienten für Geriatrie erst im Verlauf auf
- Einschätzung des Patienten hinsichtlich des voraussichtlichen Nachsorgebedarfs bereits früh möglich
- Falsche oder fehlende Angaben und Lückenfüller in Maske erschweren Arbeit des SD, dazu zählen insbesondere Kontaktdaten von Patienten und deren Angehörigen
- Prozessschritt 2: Eine ausführliche Beratung und eine psychosoziale Intervention

**Workshop 2**

- Prozess der Pflegeüberleitung startet mit Anamnese, danach darf erst zur PÜ oder EM angemeldet werden. In der Praxis ist es allerdings nicht so.
- Relevante Eingabefelder innerhalb der KLAU werden regelm. Mit Platzhaltern gefüllt. SD muss dann Informationen zur Kontaktaufnahme selber ausfindig machen. Kontaktieren sie dazu die Station, sind die MA dort genervt
- Siebter Schritt ist Kostenzusage
- Achter Schritt danach ist Entlassungstag
- Unterschiedliche Arbeitsweisen und Prozessabläufe zwischen den Abteilungen. Teilweise sehr große Bei Sozialdienst kann es sein, dass erst nach Entlassung Bearbeitung der Anträge und Beratung stattfinden.
- Aufnahme in den Pflegedienst und Beschaffung der Hilfsmittel muss gewährleistet sein, bevor Pat. Entlassen werden kann
- Es gibt Fristen, die eingehalten werden müssen
- Ohne KLAU darf SD nicht aktiv werden
- Bei Pflegeüberleitung müssen Verträge vor Entlassung geschlossen sein, keine Aufnahme von Patienten durch die Nachversorger zur KZP ohne Vertrag
- Bei onkologischen Patienten in der Chirurgie findet die Besprechung erst nach der Entlassung statt. Bei Sozialdienst kann alles auch nach der Entlassung stattfinden
- Prozessschritt 5 ist Übermittlung an Kostenträger
- Prozessschritt 6 ist Information von Patienten und Station
- Prozessschritt 7 ist Kostenzusage
- Vor Entlassung wird dem Patienten oder dessen Angehörigen eine Mail mit Information zum Entlassungsprozess gesendet. Damit werden sie auf wichtige Aspekte zur Vorbereitung hingewiesen

**Workshop 3**

- Patient muss befragt werden, ob er ins EM einwilligt und dieses durch das klinische Personal aufgenommen werden kann. Ohne Unterschrift des Patienten kann es nicht erfolgen
- Wartezeit für orthopädische Rehas liegen bei 4-8 Wochen. In Geriatrie kann erst Versorgung übernommen werden, wenn Patienten vor Ort sind. Die meisten Kliniken arbeiten nicht mit Voranmeldungen.
- Patienten werden von Ärzten und Pflege wahllos zu allen möglichen Nachsorgemöglichkeiten angemeldet. Ein konkreter Bedarf wird nicht regelhaft ermittelt oder dokumentiert, das wird dem Aufgabenfeld des Entlassmanagements zugeordnet.
- KLAU hat Struktur, die erfüllt werden muss, ansonsten kann der Auftrag nicht abgesandt werden
- Information, ob ein Pflegegrad vorliegt, muss zu Beginn des EM eingeholt werden. Falls keiner vorliegt, aber ein Pflegebedarf vorhanden ist, muss unmittelbar ein Antrag gestellt werden. Dies geschieht durch Pflegeüberleitung
- Wenn Patienten von weiter weg kommen, wird Prozess des EM früher gestartet
- Beim Aufnahmegespräch des Patienten soll Assessment durchgeführt werden, das wird aber selten getan

- Kogn. Einschränkungen, Einschränkungen im Hörverständnis, Sprachstörungen sind Aspekte, die zu Beginn des Prozesses bekannt sein sollen

**Workshop 4**

- Entlassungsprozess wird letztlich durch gesundheitliche Verfassung und Willen des Patienten beeinflusst

**Workshop 5**

- Berufsgruppen haben nicht alle die gleichen Prozesse
- Anforderungen von Stationen gehen digital an SD. Dieses Konsil wird ausgedruckt und handschriftlich weiter zur Dokumentation genutzt. Diese Informationen werden dann im IKT übernommen, sodass andere MA auch Einsicht haben. Es sind Textbausteine durch SD eingerichtet, die bei Bedarf genutzt werden
- Möglichkeiten der Partizipation des Patienten durch Einwilligung oder Ablehnen des EM
- Ausmaß des Engagements bzw. der Partizipation von Angehörigen bestimmt unmittelbar die Arbeitslast der SD im EM
- Klinikinterne Prozesse sind nicht aufeinander abgestimmt. Prozess der OP bedarf eigentlich der Abstimmung mit Prozess des EM, wird aber nicht vorgenommen. So kommt es Herausforderungen bei der Nachsorge und Versorgungsbruch.
- Anforderungen von Stationen gehen digital an SD. Dieses Konsil wird ausgedruckt und handschriftlich weiter zur Dokumentation genutzt. Diese Informationen werden dann im IKT übernommen, sodass andere MA auch Einsicht haben. Es sind Textbausteine durch SD eingerichtet, die bei Bedarf genutzt werden
- Prozessoptimierung innerhalb des EM muss bereits vor Aufnahme ins KH ansetzen
- Unterschiedliche Prozessplanung in Bereichen der Ortho und Kardio
- Prozessbeginn ist hier das Konsil. Es gibt eine MSDN-Anforderung
- Nächster Prozessschritt ist Kontrolle der Zuständigkeit
- Dringlichkeit des EM bzw. der Nachsorgeplanung lässt sich klassifizieren. Priorität sind kurzfristige Entlassungen und Palliativpatienten, die in der letzten Sterbephse sind
- Nächster Prozessschritt ist Kontaktaufnahme zum Patienten
- Beim Patienten werden während des Gesprächs durch den Sozialdienst Notizen angefertigt
- Nächster Prozessschritt ist Bedarfserhebung anhand der gesammelten Informationen.
- Was unterschreibungspflichtig ist, muss unterschrieben werden
- Nächster Prozessschritt ist suchen eines geeigneten Nachversorgers

**Workshop 6:**

- Patientenaufnahme ist der erste Kontakt für Patienten. Dort wird Karte eingelesen und Information erhoben
- Teilnehmende müssen zu Beginn des Prozesses wissen, wie pflegeaufwändig die Versorgung des Patienten ist der wird, damit sie diesen Bedarf entsprechenden abdecken kann
- Hilfsmittel müssen vor Entlassung gestellt sein, ansonsten kommt es zu Beeinträchtigung des Entlassungsprozess
- Prozess beginnt, wenn Anforderung an SD gestellt wird. Dies geschieht formal durch den Arzt. Er kann aber den Impuls von Angehörigen oder SD erhalten.
- Zu Beginn muss unbedingt erhoben werden, ob eine Versorgung bereits besteht und ob Bedarf an Anpassung besteht
- Prozessschritt: Mitarbeiter nimmt sich des Falls an und druckt ihn aus
- Nächster Prozessschritt ist direkter Kontakt zu Patient
- Wenn Bedarf erhoben wurde und Rücksprache mit anderen Disziplinen erfolgt ist, ist die Entlassplanung der nächste Schritt. Das inkludiert auch das Stellen der Anträge
- Nächster Schritt ist Information des Patienten, des Arztes und der Station
- Bei manchen Patienten kann Entlassung erst erfolgen, wen Hilfsmittel vorhanden sind

### Gesamtdarstellung aller Punkte

- Regelmäßig Beratung/Aufklärung aufgrund falsch geplanter Nachversorgung
- Es gibt eine Prozessdefinition, diese Prozessbeschreibung wird nicht regulär eingehalten
- Es gib keinen Standard. Ablauf wird mündlich weitergegeben und muss gelernt werden
- Vorgehen beim EM begründet sich durch Expertise, Erfahrung des Teilnehmenden
- Klinischer Auftrag soll bei Aufnahme, spätestens binnen 24h, erfasst und an SD weitergeleitet werden. Daran schließt Bedarfserhebung an
- Patient gilt als zum EM aufgenommen, wenn er versorgt werden möchte, allen Dokumenten zugestimmt hat und SD einen klinischen Auftrag erhält. Nicht jeder Patient hat Wunsch nach EM
- Klinischer Auftrag kann kommen von Station, Arzt oder Pflege, Patient direkt oder Bevollmächtigten. Nur die drei sind berechtigt. Ist erster Prozessschritt im EM.
- Patient muss im System angelegt und aufgeklärt sein, damit EM tätig werden kann
- Zu Beginn des Prozesses erfolgt persönliche Kontaktaufnahme mit Patient oder Angehörigen, dieser Erstkontakt dient der Bedarfserhebung
- Verweildauer ist abhängig von Erkrankung
- Verweildauer mancher Patienten teilweise Jahre. Kommen ein bis zwei Mal pro Monat. Bei ihnen wir Zeit abgekappt.
- Ist Pflegebedarf erhoben, wird persönlich mit Pat./Angehörigen gesprochen und häusliches/pflegerisches Setting erfragt
- Ein etwaiger Pflegegrad des Patienten soll bei Aufnahme hinterlegt werden
- EM nimmt Kontakt zu Ärzten und Pflege auf um Entlasstag zu erfragen und Ersteinschätzung zu erhalten. Barthel-Index ist sehr wichtig

- Bei planbaren Eingriffen kontaktiert SD Patienten im Vorfeld und klärt Antragstellung ab
- Tatsächlicher Hilfebedarf einer Person fällt insbesondere bei Patienten für Geriatrie erst im Verlauf auf
- Einschätzung des Patienten hinsichtlich des voraussichtlichen Nachsorgebedarfs bereits früh möglich
- Prozessschritt 2: Eine ausführliche Beratung und eine psychosoziale Intervention
- Relevante Eingabefelder innerhalb der KLAU werden regelm. Mit Platzhaltern gefüllt. SD muss dann Informationen zur Kontaktaufnahme selber ausfindig machen. Kontaktieren sie dazu die Station, sind die MA dort genervt
- Siebter Schritt ist Kostenzusage
- Achter Schritt danach ist Entlassungstag
- Unterschiedliche Arbeitsweisen und Prozessabläufe zwischen den Abteilungen. Teilweise sehr große Bei Sozialdienst kann es sein, dass erst nach Entlassung Bearbeitung der Anträge und Beratung stattfinden.
- Aufnahme in den Pflegedienst und Beschaffung der Hilfsmittel muss gewährleistet sein, bevor Pat. Entlassen werden kann
- Es gibt Fristen, die eingehalten werden müssen
- Bei Pflegeüberleitung müssen Verträge vor Entlassung geschlossen sein, keine Aufnahme von Patienten durch die Nachversorger zur KZP ohne Vertrag
- Bei onkologischen Patienten in der Chirurgie findet die Besprechung erst nach der Entlassung statt. Bei Sozialdienst kann alles auch nach der Entlassung stattfinden
- Prozessschritt 5 ist Übermittlung an Kostenträger
- Prozessschritt 6 ist Information von Patienten und Station
- Prozessschritt 7 ist Kostenzusage
- Vor Entlassung wird dem Patienten oder dessen Angehörigen eine Mail mit Information zum Entlassungsprozess gesendet. Damit werden sie auf wichtige Aspekte zur Vorbereitung hingewiesen
- Patient muss befragt werden, ob er ins EM einwilligt und dieses durch das klinische Personal aufgenommen werden kann. Ohne Unterschrift des Patienten kann es nicht erfolgen. In der Praxis ist es allerdings nicht so.
- Wartezeit für orthopädische Rehas liegen bei 4-8 Wochen. In Geriatrie kann erst Versorgung übernommen werden, wenn Patienten vor Ort sind. Die meisten Kliniken arbeiten nicht mit Voranmeldungen.
- Patienten werden von Ärzten und Pflege wahllos zu allen möglichen Nachsorgemöglichkeiten angemeldet. Ein konkreter Bedarf wird nicht regelhaft ermittelt oder dokumentiert, das wird dem Aufgabenfeld des Entlassmanagements zugeordnet.
- KLAU hat Struktur, die erfüllt werden muss, ansonsten kann der Auftrag nicht abgesandt werden
- Information, ob ein Pflegegrad vorliegt, muss zu Beginn des EM eingeholt werden. Falls keiner vorliegt, aber ein Pflegebedarf vorhanden ist, muss unmittelbar ein Antrag gestellt werden. Dies geschieht durch Pflegeüberleitung
- Wenn Patienten von weiter weg kommen, wird Prozess des EM früher gestartet
- Beim Aufnahmegespräch des Patienten soll Assessment durchgeführt werden, das wird aber selten getan

- Kogn. Einschränkungen, Einschränkungen im Hörverständnis, Sprachstörungen sind Aspekte, die zu Beginn des Prozesses bekannt sein sollen
- Entlassungsprozess wird letztlich durch gesundheitliche Verfassung und Willen des Patienten beeinflusst
- Berufsgruppen haben nicht alle die gleichen Prozesse
- Anforderungen von Stationen gehen digital an SD. Dieses Konsil wird ausgedruckt und handschriftlich weiter zur Dokumentation genutzt. Diese Informationen werden dann im IKT übernommen, sodass andere MA auch Einsicht haben. Es sind Textbausteine durch SD eingerichtet, die bei Bedarf genutzt werden
- Möglichkeiten der Partizipation des Patienten durch Einwilligung oder Ablehnen des EM
- Ausmaß des Engagements bzw. der Partizipation von Angehörigen bestimmt unmittelbar die Arbeitslast der SD im EM
- Klinikinterne Prozesse sind nicht aufeinander abgestimmt. Prozess der OP bedarf eigentlich der Abstimmung mit Prozess des EM, wird aber nicht vorgenommen. So kommt es Herausforderungen bei der Nachsorge und Versorgungsbruch.
- Anforderungen von Stationen gehen digital an SD. Dieses Konsil wird ausgedruckt und handschriftlich weiter zur Dokumentation genutzt. Diese Informationen werden dann im IKT übernommen, sodass andere MA auch Einsicht haben. Es sind Textbausteine durch SD eingerichtet, die bei Bedarf genutzt werden
- Prozessoptimierung innerhalb des EM muss bereits vor Aufnahme ins KH ansetzen
- Unterschiedliche Prozessplanung in Bereichen der Ortho und Kardio
- Prozessbeginn ist hier das Konsil. Es gibt eine MSDN-Anforderung
- Nächster Prozessschritt ist Kontrolle der Zuständigkeit
- Dringlichkeit des EM bzw. der Nachsorgeplanung lässt sich klassifizieren. Priorität sind kurzfristige Entlassungen und Palliativpatienten, die in der letzten Sterbephse sind
- Nächster Prozessschritt ist Kontaktaufnahme zum Patienten
- Beim Patienten werden während des Gesprächs durch den Sozialdienst Notizen angefertigt
- Nächster Prozessschritt ist Bedarfserhebung anhand der gesammelten Informationen.
- Was unterschreibungspflichtig ist, muss unterschrieben werden
- Nächster Prozessschritt ist suchen eines geeigneten Nachversorgers
- Patientenaufnahme ist der erste Kontakt für Patienten. Dort wird Karte eingelesen und Information erhoben
- Teilnehmende müssen zu Beginn des Prozesses wissen, wie pflegeaufwändig die Versorgung des Patienten ist der wird, damit sie diesen Bedarf entsprechenden abdecken kann
- Hilfsmittel müssen vor Entlassung gestellt sein, ansonsten kommt es zu Beeinträchtigung des Entlassungsprozess
- Prozess beginnt, wenn Anforderung an SD gestellt wird. Dies geschieht formal durch den Arzt. Er kann aber den Impuls von Angehörigen oder SD erhalten.

- Zu Beginn muss unbedingt erhoben werden, ob eine Versorgung bereits besteht und ob Bedarf an Anpassung besteht
- Prozessschritt: Mitarbeiter nimmt sich des Falls an und druckt ihn aus
- Nächster Prozessschritt ist direkter Kontakt zu Patient
- Wenn Bedarf erhoben wurde und Rücksprache mit anderen Disziplinen erfolgt ist, ist die Entlassplanung der nächste Schritt. Das inkludiert auch das Stellen der Anträge
- Nächster Schritt ist Information des Patienten, des Arztes und der Station
- Bei manchen Patienten kann Entlassung erst erfolgen, wen Hilfsmittel vorhanden sind

## Unterkategorie Zeitliche Abläufe

### Aussagen nach Workshops

#### Workshop 1

- Bei planbaren Eingriffen kontaktiert SD Patienten im Vorfeld und klärt Antragstellung ab
- Planung vorab bei akuten Erkrankungen nicht möglich
- Patienten kommen unangekündigt bei SD vorbei
- Zuerst persönliche Kontaktaufnahme mit Pat. Oder Angehörigen
- Patienten können Termin ausmachen.

#### Workshop 2

- Herausforderungen beim KLAU sind, dass Patientendaten nicht vollständig oder inkorrekt sind oder dass unsinnige bzw. unklare Aufträge erteilt werden
- Es kommt oft vor, dass Angehörige oder Ärzte das EM einschalten wollen, aber kein KLAU angelegt ist

#### Workshop 3

- Eilantrag wird innerhalb von Sieben Werktagen per Aktenlage bestimmt. Normaler Pflegeantrag benötigt vier bis sechs Wochen Bearbeitungszeit
- Wenn Patienten von weiter weg kommen, wird Prozess des EM früher gestartet

#### Workshop 4

- Entlassungsprozess wird letztlich durch Verfassung (und Willen?) des Patienten beeinflusst
- Jüngere Patienten haben ihr Smartphone immer bei sich. TN leitet sie an, sich eigenständig zu informieren und den Kontakt zur Krankenkasse herzustellen. Diese Patienten nehmen das positiv auf, weil sie dann während des Klinikaufenthalts Beschäftigung hätten

#### Workshop 6:

- Wenn MA keine Kenntnis über demenz. Erkrankung eines Pat. Haben verlängert und erschwert das den Prozess

### Gesamtdarstellung aller Punkte

- Bei planbaren Eingriffen kontaktiert SD nach Möglichkeit Patienten im Vorfeld und klärt Antragstellung ab, Planung vorab bei akuten Erkrankungen nicht möglich
- Patienten kommen teilweise unangekündigt bei SD vorbei
- Zuerst persönliche Kontaktaufnahme mit Patienten Oder Angehörigen
- Patienten können auch Termin ausmachen
- Herausforderungen beim KLAU sind, dass Patientendaten nicht vollständig oder inkorrekt sind oder dass unsinnige bzw. unklare Aufträge erteilt werden
- Es kommt oft vor, dass Angehörige oder Ärzte das EM einschalten wollen, aber kein KLAU angelegt ist
- Eilantrag wird innerhalb von Sieben Werktagen per Aktenlage bestimmt. Normaler Pflegeantrag benötigt vier bis sechs Wochen Bearbeitungszeit
- Wenn Patienten von weiter weg kommen, wird Prozess des EM früher gestartet
- Entlassungsprozess wird letztlich durch Verfassung (und Willen?) des Patienten beeinflusst
- Jüngere Patienten haben ihr Smartphone immer bei sich. TN leitet sie an, sich eigenständig zu informieren und den Kontakt zur Krankenkasse herzustellen. Diese Patienten nehmen das positiv auf, weil sie dann während des Klinikaufenthalts Beschäftigung hätten
- Wenn MA keine Kenntnis über demenzielle Erkrankung eines Pat. Haben verlängert und erschwert das den Prozess

## Kategorie Akteure und Zuständigkeiten

### Unterkategorie Beteiligte Rollen

#### Aussagen nach Workshops

**Workshop 1**

- Großes Arbeitsaufkommen durch (manuelle) Bearbeitung der Anrufe
- Patient, Arzt, Pflege und Betreuer sind „inner circle“, Patient ist Kern des Prozesses
- Patienten kommen unangekündigt bei SD vorbei
- Zuerst persönliche Kontaktaufnahme mit Pat. Oder Angehörigen
- Patienten können Termin ausmachen.

**Workshop 2**

- Das Lebensalter der Patienten ist ein Einflussfaktor auf die Nachversorgung. Personen im mittleren Alter (55 – 65) ohne unterstützendes soziales/häusliches Umfeld haben bei Bedarf der Versorgung nach Klinikaufenthalt Schwierigkeiten, einen Platz zu bekommen, da Pflegeheime eine KZP meist erst ab dem 65. Lebensjahr akzeptieren.
- Patienten, die nicht rehafähig sind, zuhause aber keine Unterstützung erwarten können, können schwer entlassen werden
- Nachsorge zuhause gelingt eher, wenn Angehörige mithelfen.

**Workshop 3**

- Einwilligung in EM muss von Pat. unterschrieben sein
- Angehörige rufen wiederholt die gleiche Person an, obwohl sie ihnen schon erklärt hat, dass sie nicht zuständig ist.
- Angehörige sind oft in emotionalen Ausnahmesituationen. Dadurch sinkt ihre Konzentrations- und Merkfähigkeit
- Patient muss befragt werden, ob er ins EM einwilligt
- EM ist in der Chirurgie teilweise sehr herausfordernd, da dort häufig Obdachlose landen, die versorgt werden müssen

**Workshop 4**

- Hilfe wird von Patient:innen scheinbar oft konkret bei einer (Bezugs-)Person gesucht
- Mache Patienten/Angehörige wissen nicht, dass sie einen Pflegegrad selber beantragen können. Es kommt vor, dass sie das Dokument beim Stationsarzt hinterlegen und darauf warten, dass dieser auf sie zukommt
- Manche Privatpatienten sind nicht im Bilde darüber, welche Leistungen ihre Police abdeckt
- Betreuungsverfahren als Folge von geschäftsunfähigkeit und fehlender Betreuungsverfügung
- Beratung zu Versorgungsleistungen muss auf jeweilige Person und Situation abgestimmt sein. Faktoren wie Art der Versicherung, gewünschtes Pflegesetting und vorhandene Ressourcen bestimmen den Prozess maßgeblich

**Workshop 5**

- Patient als Stakeholder
- Angehörige und Betreuer
- Sorgeberechtigte
- Möglichkeiten der Partizipation des Pat. Durch Annahme oder Ablehnen des EM
- Es gibt Patienten, die EM regelrecht einfordern
- Ausmaß des Engagements bzw. der Partizipation von Angehörigen bestimmt unmittelbar die Arbeitslast der SD im EM
- Angehörige sind oft fordernd. Möchten in kurzer Zeit Versorgungslösungen. Sie sind auch verärgert, wie Ärzte Versprechungen machen, die der SD nicht einhalten kann.
- Angehörige informieren sich im Internet. Haben Anspruchshaltung und kommen aktiv mit Forderungen auf SD zu.
- Angehörige wirken oft hilflos und uninformiert. MA klärt dann zu Möglichkeiten der Versorgung auf
- Angehörige werden ungehalten. SD beendet dann Gespräch
- Bei korrekter Afklärung vor med Eingriff hat der Patient noch die Chance, die Nachsorge selbst mitzugestalten.
- Häufig können Patienten ihre eigenen Bedarfe nicht nennen. SD nimmt dann Kontakt zu Angehörigen auf, um mit diesen die Bedarfe zu besprechen. Dies geschieht tlw. Gemeinsam mit Pat.

**Workshop 6:**

- Kontakt mit Angehörigen kann herausfordernd sein, wenn mehrere eines Patienten anrufen und Auskunft oder Beratung wollen, weil die Angehörigen sich untereinander nicht im Austausch befinden
- SD macht mit Angehörigen Termin aus, wenn diese in den Prozess involviert werden müssen

**Workshop 5**

- Krankenkassen
- Rentenversicherung
- Sozialamt
- Private Kostenträger wie private Krankenversicherung oder Pflegeversicherung
- Privatkassen und Beihilfen sind zu 99% nicht zu erreichen. Sprechen auch nicht mit SD, nur mit Patienten. Kostenübernahmebescheide senden sie ausschließlich an Anschrift des Patient
- Unfallträger
- Jugendamt
- Berufsgenossenschaft
- DRV-Bund hat feste Zuständigkeiten und Hotline für Sozialdienste

**Workshop 1**

- Familienhilfe, Pflegestützpunk und Rehakliniken sind Stakeholder
- Versorgungsamt
- Intensivpflege
- Spezialklinik
- Rehakliniken sind fast wichtigste Prozessbeteiligte für geriatrische Abteilung
- Patient, Arzt, Pflege und Betreuer sind „inner circle“
- SAPV
- SD und PÜ haben unterschiedliche Prozessabläufe
- Vorgehen beim EM begründet sich durch Expertise, Erfahrung des TN
- Stomatherapie und -beratung leisten pflegerische Betreuung
- Zuerst persönliche Kontaktaufnahme mit Pat. Oder Angehörigen
- Patienten können Termin ausmachen.
- Negative Vorerfahrung der SD-MA, weil Pat. Nicht von Arzt erfuhr, dass er Palliativstatus hat.
- Oberarzt ordnet Entlassung an, wenn Patient zulange hospitiert ist und dadurch Kosten entstehen

**Workshop 2**

- Unterschiedliche Vorgehensweisen der Professionen, bei Sozialdienst kann es sein, dass nach Entlassung Anträge und Beratung stattfinden. Bei Pflegeüberleitung muss dies vor der Entlassung geschehen
- Bei Sozialdienst kann Beratungsgespräch mit Patient:innen auch nach der Entlassung stattfinden
- Ärzte füllen notwendige Unterlagen spät, falsch oder gar nicht aus.

**Workshop 3**

- Wartezeit für orthopädische Rehas liegen bei 4-8 Wochen. In Geriatrie kann erst Versorgung übernommen werden, wenn Patienten vor Ort sind. Die meisten Kliniken arbeiten nicht mit Voranmeldungen.
- Es müssen dutzende Pflegeheime kontaktiert werden, bis ein Pflegeplatz zu finden ist. Der Prozess erfordert auch viel Wartezeit
- Herausfordernd ist, dass Pflegedienste in häuslicher Umgebung des Patienten erst herausgesucht werden und dann kontaktiert werden müssen
- Klinisches Personal gibt regelmäßig an, dass Pat. nicht befragt werden kann, obwohl er adäquat ist.
- Nicht jede Berufsgruppe braucht für ihre Tätigkeit die gleichen Informationen
- Berufsgruppen haben wenig Kenntnis davon, welche andere Professionen im Haus vorhanden sind und was in deren Zuständigkeit fällt
- Ärzte werden seitens der Organisation nicht wirksam aufgeklärt, welche Berufsgruppe welche Zuständigkeit hat.
- In der pfleg. Anamnese werden Informationen (auch) nicht eingeholt

**Workshop 4**

- Seniorenberatungsstellen und Pflegestützpunkte sind Akteure

**Workshop 5**

- SD berät und empfiehlt Patienten zu der Versorgungsform, die er bedarf und die auch faktisch umsetzbar ist
- Selbstverständnis des SD als empathischen Beruf
- Berufsgruppen haben nicht alle die gleichen Prozesse
- Leistungserbringer sind Ärzte und Pflege, Hilfsmittel-Lieferanten, Pflegedienste, Physiotherapie, Pflegeüberleitung, Sanitätshäuser, Apotheken, weiterbehandelnde Ärzt:innen, externe Praxen, stationäre Einrichtungen, Pflegeheime, Spezialisierte ambulante palliative Pflege, Hospize, Betreutes Wohnen, Spezialanbieter für parenterale Ernährung, Stoma- und Tracheostomieversorgung sowie Reha-Einrichtungen
- Es kann zwischen direkten und indirekten Stakeholdern unterschieden werden
- Kurzzeitpflegeplätze sind nie dringend, weil man lange warten muss
- Meiste Herausforderungen gibt es in der Zusammenarbeit mit Ärzten
- Ärzte versprechen Pat. Dinge, die gesetzlich nicht bestand haben
- Großes Problem ist Erreichbarkeit von Ärzten
- SD hat keine festen zuständigen Ansprechpartner im Bereich der Ärzte. Verantwortlichkeiten wechseln mitunter sehr schnell. Ärzte verweisen auf andere Zuständigkeit, Prozess des EM wird dadurch insgesamt negativ beeinflusst
- Wer letztlich wann entlassen wird, entscheidet der Oberarzt
- Ärzte führen eine vermeintliche Beratung der Patienten durch bzw. stellen diesen Versorgungsmaßnahmen in Aussicht, die so nicht umsetzbar sind
- Für Arzt ist es dringend, weil er Pat. Entlassen will

## Gesamtdarstellung aller Punkte

- Großes Arbeitsaufkommen durch (manuelle) Bearbeitung der Anrufe
- Patient, Arzt, Pflege und Betreuer sind „inner circle“
- Patient ist Kern des Prozesses
- Patienten kommen unangekündigt bei SD vorbei
- Patienten können Termin ausmachen.
- Das Lebensalter der Patienten ist ein Einflussfaktor auf die Nachversorgung. Personen im mittleren Alter (55 – 65) ohne unterstützendes soziales/häusliches Umfeld haben bei Bedarf der Versorgung nach Klinikaufenthalt Schwierigkeiten, einen Platz zu bekommen, da Pflegeheime eine KZP meist erst ab dem 65. Lebensjahr akzeptieren.
- Patienten, die nicht rehafähig sind, zuhause aber keine Unterstützung erwarten können, können schwer entlassen werden
- Patient muss ins EM einwilligen, Einwilligung in EM muss von Patienten unterschrieben sein
- Manche Privatpatienten sind nicht im Bilde darüber, welche Leistungen ihre Police abdeckt
- Betreuungsverfahren als Folge von Geschäftsunfähigkeit und fehlender Betreuungsverfügung
- Nachsorge zuhause gelingt eher, wenn Angehörige mithelfen.
- Angehörige rufen wiederholt die gleiche Person an, obwohl sie ihnen schon erklärt hat, dass sie nicht zuständig ist.
- Angehörige sind oft in emotionalen Ausnahmesituationen. Dadurch sinkt ihre Konzentrations- und Merkfähigkeit
- EM ist in der Chirurgie teilweise sehr herausfordernd, da dort häufig Obdachlose landen, die versorgt werden müssen
- Hilfe wird scheinbar oft konkret bei einer (Bezugs) Person gesucht
- Mache Patienten/Angehörige wissen nicht, dass sie einen Pflegegrad selber beantragen können. Sie hinterlegen das Dokument beim Stationsarzt und warten darauf, dass dieser auf sie zukommt
- Patient, Angehörige, Sorgeberechtigte und Betreuer als Stakeholder
- Möglichkeiten der Partizipation des Pat. Durch Annahme oder Ablehnen des EM
- Es gibt Patienten, die EM regelrecht einfordern
- Ausmaß des Engagements bzw. der Partizipation von Angehörigen bestimmt unmittelbar die Arbeitslast der SD im EM
- Angehörige sind oft fordernd. Möchten in kurzer Zeit Versorgungslösungen. Sie sind auch verärgert, wie Ärzte Versprechungen machen, die der SD nicht einhalten kann.
- Angehörige informieren sich im Internet. Haben Anspruchshaltung und kommen aktiv mit Forderungen auf SD zu.
- Angehörige wirken oft hilflos und uninformiert. MA klärt dann zu Möglichkeiten der Versorgung auf
- Angehörige werden ungehalten. SD beendet dann Gespräch
- Bei korrekter Aufklärung vor elektivem Eingriff hat der Patient noch die Chance, die Nachsorge selbst mitzugestalten.
- Häufig können Patienten ihre eigenen Bedarfe nicht nennen. SD nimmt dann Kontakt zu Angehörigen auf, um mit diesen die Bedarfe zu besprechen. Dies geschieht tlw. Gemeinsam mit Patienten

- Kontakt mit Angehörigen kann herausfordernd sein, wenn mehrere eines Patienten anrufen und Auskunft oder Beratung wollen, weil die Angehörigen sich untereinander nicht im Austausch befinden
- SD macht mit Angehörigen Termin aus, wenn diese in den Prozess involviert werden müssen
- Krankenkassen
- Rentenversicherung
- Sozialamt
- Private Kostenträger wie private Krankenversicherung oder Pflegeversicherung
- Privatkassen und Beihilfen sind zu 99% nicht zu erreichen. Sprechen auch nicht mit SD, nur mit Patienten. Kostenübernahmebescheide senden sie ausschließlich an Anschrift des Patient
- Unfallträger
- Jugendamt
- Berufsgenossenschaft
- DRV-Bund hat feste Zuständigkeiten und Hotline für Sozialdienste
- Familienhilfe, Pflegestützpunk, Seniorenberatungsstellen und Rehakliniken sind Stakeholder
- Versorgungsamt
- Intensivpflege
- Spezialklinik
- Rehakliniken sind fast wichtigste Prozessbeteiligte in der Geriatrie
- Patient, Arzt, Pflege und Betreuer sind „inner circle“
- SAPV
- SD und PÜ haben unterschiedliche Prozessabläufe
- Vorgehen beim EM begründet sich durch Expertise, Erfahrung des TN
- Bei Sozialdienst kann Beratungsgespräch mit Patient:innen auch nach der Entlassung stattfinden
- Unterschiedliche Vorgehensweisen der Professionen, bei Sozialdienst kann es sein, dass nach Entlassung Anträge und Beratung stattfinden. Bei Pflegeüberleitung muss dies vor der Entlassung geschehen
- Nicht jede Berufsgruppe braucht für ihre Tätigkeit die gleichen Informationen?
- Negative Vorerfahrung der SD-MA, weil Pat. Nicht von Arzt erfuhr, dass er Palliativstatus hat.
- Oberarzt ordnet Entlassung an, wenn Patient zulange hospitiert ist und dadurch Kosten entstehen
- Ärzte füllen notwendige Unterlagen spät, falsch oder gar nicht aus.
- Ärzte werden seitens der Organisation nicht wirksam aufgeklärt, welche Berufsgruppe welche Zuständigkeit hat.
- Stomatherapie und -beratung leisten pflegerische Betreuung
- Wartezeit für orthopädische Rehas liegen bei 4-8 Wochen. In Geriatrie kann erst Versorgung übernommen werden, wenn Patienten vor Ort sind. Die meisten Kliniken arbeiten nicht mit Voranmeldungen.
- Es müssen dutzende Pflegeheime kontaktiert werden, bis ein Pflegeplatz zu finden ist. Der Prozess erfordert auch viel Wartezeit
- Herausfordernd ist, dass Pflegedienste in häuslicher Umgebung des Patienten erst herausgesucht werden und dann kontaktiert werden müssen

- Klinisches Personal gibt regelmäßig an, dass Pat. nicht befragt werden kann, obwohl er adäquat ist.
- Berufsgruppen haben wenig Kenntnis davon, welche andere Professionen im Haus vorhanden sind und was in deren Zuständigkeit fällt
- In der pfleg. Anamnese werden Informationen (auch) nicht eingeholt
- SD berät und empfiehlt Patienten zu der Versorgungsform, die er bedarf und die auch faktisch umsetzbar ist
- Selbstverständnis des SD als empathischen Beruf
- Berufsgruppen haben nicht alle die gleichen Prozesse
- Leistungserbringer sind Ärzte und Pflege, Hilfsmittel-Lieferanten, Pflegedienste, Physiotherapie, Pflegeüberleitung, Sanitätshäuser, Apotheken, weiterbehandelnde Ärzt:innen, externe Praxen, stationäre Einrichtungen, Pflegeheime, Spezialisierte ambulante palliative Pflege, Hospize, Betreutes Wohnen, Spezialanbieter für parenterale Ernährung, Stoma- und Tracheostomieversorgung sowie Reha-Einrichtungen
- Es kann zwischen direkten und indirekten Stakeholdern unterschieden werden
- Kurzzeitpflegeplätze sind nie dringend, weil man lange warten muss
- Meiste Herausforderungen gibt es in der Zusammenarbeit mit Ärzten
- Ärzte versprechen Patienten Dinge, die gesetzlich nicht bestand haben
- Großes Problem ist Erreichbarkeit von Ärzten
- SD hat keine festen zuständigen Ansprechpartner im Bereich der Ärzte. Verantwortlichkeiten wechseln mitunter sehr schnell. Ärzte verweisen auf andere Zuständigkeit, Prozess des EM wird dadurch insgesamt negativ beeinflusst
- Wer letztlich wann entlassen wird, entscheidet der Oberarzt
- Ärzte führen eine vermeintliche Beratung der Patienten durch bzw. stellen diesen Versorgungsmaßnahmen in Aussicht, die so nicht umsetzbar sind

## Unterkategorie Verantwortlichkeiten

### Aussagen nach Workshops

#### Workshop 1

- Unterschiedliche Verantwortlichkeiten und Prozessabläufe bei SD und PÜ
- Zuständigkeiten werden gegenseitig verordnet
- Oberarzt verhindert Maßnahmen, wenn diese nicht ausreichend finanziert werden. Auch, wenn diese medizinisch sinnvoll sind. Er ordnet Entlassung an, wenn Patient zulange hospitiert ist und dadurch Kosten entstehen
- Patient ist Auftraggeber
- Finanzierung von KK abhängig, diese haben Weisungsbefugnis
- Es besteht ein pflegerische und ärztl. Standard zum Entlassmanagement. Dieser wird aber meist nicht eingehalten. Für EM benötigter Input durch andere Berufsgruppen ist größtenteils nicht gegeben, sodass EM Prozessablauf ändern und fehlende Prozessschritte kompensieren muss. SD übernimmt Aufgabe der Pflege, die nicht abgerechnet werden können, damit Entlassung gelingt.
- MA des SD hat mehrere Stationen zu betreuen, haben feste Regelung der Zuständigkeit
- Barthel-Index wird meist nicht ohne Aufforderung erhoben, obwohl er Pflicht ist
- Barthel-Index ist pfleg. Aufgabe. Index kann abgerechnet werden

- Pfleg. Assessment ist oft nicht hinterlegt
- Für das EM relevante Daten und Informationen werden nicht immer von Ärzten und Pflege erhoben oder zur Verfügung gestellt. Drittmittelabteilung führt öfter nicht Maßnahmen zur Aufnahme und Abrechnung durch
- Stationen kennen Ansprechpartner besser. Sowohl Ärzte, Pfleger und Patienten sind personenfixiert
- Aktualisierung der Assessment durch Pflege erfolgt nicht, ist Problem für Einschätzung durch SD
- Dokumentation der Pflege oft unvollständig. PK haben keine Zeit oder kennen Pat. Noch nicht
- SD übernimmt Aufgabe der Pflege, die nicht abgerechnet werden können, damit Entlassung gelingt.
- SD muss Zeit aufwenden, um Daten zu erhalten, die eigentlich durch Aufnahme erhoben werden sollte. Andere Instanzen benötigen diese Daten auch. PK fühlen sich belästigt von Informationssuche des SD
- Pflegegrad wird nicht dokumentiert, obwohl oft angesprochen. Sollte spätestens bei Aufnahme auf Station dokumentiert sein
- Negative Vorerfahrung der SD-MA, weil Pat. Nicht von Arzt erfuhr, dass er Palliativstatus hat.

**Workshop 2**

- Manche Personen finden den Kontakt des SD nicht in Webpräsens
- Bei Anliegen in Zusammenhang mit IT geben die zuständigen Kollegen keine zuverlässige Auskunft bzw. Hilfestellung. Man muss wissen, welcher Ansprechpartner Kompetenzen im jeweiligen Bereich hat.
- Prozesse im Haus wurden angepasst, weil Assessments nicht zuverlässig durchgeführt werden und Kommunikation zum SD durch med. P. nicht autonom vorgenommen wird. Folge ist, dass SD nun regelmäßigen Termin mit Ärzten hat, um alle Patienten hinsichtlich eines Nachsorgebedarfes zu besprechen.
- Wenn in der Cirurgie keine Tagesvisite durch SD gemacht wird, werden Patienten (durch die Ärzte?) gar nicht angemeldet. SD muss proaktiv auf Ärzte zugehen, damit Pat. Nachsorge vermittelt bekommt

**Workshop 3**

- Zuständigkeit des Sozialdienstes liegt bei Rega und Sozialberatung. Sozialdienste in anderen Häusern übernehmen sowohl Pflegeüberleitung als auch Sozialdienst
- Verschlechtert sich der Allgemeinzustand eines Patienten oder hat er noch keinen PG, wird einer von der Pflegeüberleitung beantragt
- Pflege gibt Angehörigen die Telefonnummer des Sozialdienstes, weil sie dessen Zuständigkeit vermutet. Die ist aber im Bereich der KZP nicht gegeben, also muss TN den Angehörigen erklären, dass sie die falsche Ansprechpartnerin für ihn ist. Das ist zeitintensiv für MA und Angehörigen
- Kontaktdaten werden in Patientenaufnahme erhoben und in Aufnahmebrief geschrieben
- Berufsgruppen haben wenig Kenntnis davon, welche andere Professionen im Haus vorhanden sind und was in deren Zuständigkeit fällt

- Wenn Patient selbst nicht in der Lage ist, zu telefonieren, müssen Ärzte eine gesetzliche Betreuung in die Wege leiten. Das wollen sie oft nicht machen
- Beim Aufnahmegespräch soll Assessment durchgeführt werden, das macht aber niemand
- Als zuständiger Arzt ist meist Stationsarzt eingetragen, TN muss erst herausfinden, wer der zuständige Arzt ist

**Workshop 4**

- Wenn TN Patienten auffordert, einen Pflegeantrag auszufüllen, tun das nur zwei von zehn. Die anderen acht möchten, dass die TN es macht

**Workshop 5**

- Im Kern entsteht ein Problem daraus, dass Patienten vor Eingriffen nicht umfangreich aufgeklärt/beraten werden
- Zuständigkeit des SD ist nach Organisationseinheit geregelt
- SD hat keine festen zuständigen Ansprechpartner im Bereich der Ärzte. Verantwortlichkeiten wechseln mitunter sehr schnell. Ärzte verweisen auf andere Zuständigkeit, Prozess des EM wird dadurch insgesamt negativ beeinflusst
- Kommunikation mit Kostenträgern herausfordrend. Konkrete Ansprechpartner sind nicht leicht ausfindig zu machen. Nicht ausreichend besetzte Hotlines erschweren die Recherche
- i.d.R. kein fester Kontakt bei KK für SD
- Oft kommt es dazu, dass die Anforderungen an den falschen Ansprechpartner gesandt werden. Das liegt daran, dass es mehrere Sozialarbeiter gibt. Manche gehören fest zu Organisationseinheiten.
- Wer letztlich wann entlassen wird, entscheidet der Oberarzt

**Workshop 6:**

- Welche Akteure im engeren Kontakt mit dem EM stehen, hängt von der jeweiligen Abteilung ab, für die der Prozess durchgeführt wird.
- Patientenaufnahme ist zuständig dafür, Informationen zum Patienten zu erheben und dokumentieren

## Gesamtdarstellung aller Punkte

- Unterschiedliche Verantwortlichkeiten und Prozessabläufe bei SD und PÜ
- Zuständigkeiten werden gegenseitig verordnet
- Oberarzt verhindert Maßnahmen, wenn diese nicht ausreichend finanziert werden. Auch, wenn diese medizinisch sinnvoll sind. Er ordnet Entlassung an, wenn Patient zulange hospitiert ist und dadurch Kosten entstehen
- Patient ist Auftraggeber
- Finanzierung von KK abhängig, diese haben Weisungsbefugnis
- Es besteht ein pflegerische und ärztl. Standard zum Entlassmanagement. Dieser wird aber meist nicht eingehalten. Für EM benötigter Input durch andere Berufsgruppen ist größtenteils nicht gegeben, sodass EM

Prozessablauf ändern und fehlende Prozessschritte kompensieren muss. SD übernimmt Aufgabe der Pflege, die nicht abgerechnet werden können, damit Entlassung gelingt.

- MA des SD hat mehrere Stationen zu betreuen, haben feste Regelung der Zuständigkeit
- Barthel-Index wird meist nicht ohne Aufforderung erhoben, obwohl er Pflicht ist
- Barthel-Index ist pfleg. Aufgabe. Index kann abgerechnet werden
- Pfleg. Assessment ist oft nicht hinterlegt
- Für das EM relevante Daten und Informationen werden nicht immer von Ärzten und Pflege erhoben oder zur Verfügung gestellt. Drittmittelabteilung führt öfter nicht Maßnahmen zur Aufnahme und Abrechnung durch
- Stationen kennen Ansprechpartner besser. Sowohl Ärzte, Pfleger und Patienten sind personenfixiert
- Aktualisierung der Assessment durch Pflege erfolgt nicht, ist Problem für Einschätzung durch SD
- Dokumentation der Pflege oft unvollständig. PK haben keine Zeit oder kennen Pat. Noch nicht
- SD übernimmt Aufgabe der Pflege, die nicht abgerechnet werden können, damit Entlassung gelingt.
- SD muss Zeit aufwenden, um Daten zu erhalten, die eigentlich durch Aufnahme erhoben werden sollte. Andere Instanzen benötigen diese Daten auch. PK fühlen sich belästigt von Informationssuche des SD
- Pflegegrad wird nicht dokumentiert, obwohl oft angesprochen. Sollte spätestens bei Aufnahme auf Station dokumentiert sein
- Negative Vorerfahrung der SD-MA, weil Pat. Nicht von Arzt erfuhr, dass er Palliativstatus hat.
- Manche Personen finden den Kontakt des SD nicht in Webpräsens
- Bei Anliegen in Zusammenhang mit IT geben die zuständigen Kollegen keine zuverlässige Auskunft bzw. Hilfestellung. Man muss wissen, welcher Ansprechpartner Kompetenzen im jeweiligen Bereich hat.
- Prozesse im Haus wurden angepasst, weil Assessments nicht zuverlässig durchgeführt werden und Kommunikation zum SD durch med. P. nicht autonom vorgenommen wird. Folge ist, dass SD nun regelmäßigen Termin mit Ärzten hat, um alle Patienten hinsichtlich eines Nachsorgebedarfes zu besprechen.
- Wenn in der Cirurgie keine Tagesvisite durch SD gemacht wird, werden Patienten (durch die Ärzte?) gar nicht angemeldet. SD muss proaktiv auf Ärzte zugehen, damit Pat. Nachsorge vermittelt bekommt
- Zuständigkeit des Sozialdienstes liegt bei Rega und Sozialberatung. Sozialdienste in anderen Häusern übernehmen sowohl Pflegeüberleitung als auch Sozialdienst
- Verschlechtert sich der Allgemeinzustand eines Patienten oder hat er noch keinen PG, wird einer von der Pflegeüberleitung beantragt
- Pflege gibt Angehörigen die Telefonnummer des Sozialdienstes, weil sie dessen Zuständigkeit vermutet. Die ist aber im Bereich der KZP nicht gegeben, also muss TN den Angehörigen erklären, dass sie die falsche Ansprechpartnerin für ihn ist. Das ist zeitintensiv für MA und Angehörigen
- Kontaktdaten werden in Patientenaufnahme erhoben und in Aufnahmebrief geschrieben

- Berufsgruppen haben wenig Kenntnis davon, welche andere Professionen im Haus vorhanden sind und was in deren Zuständigkeit fällt
- Wenn Patient selbst nicht in der Lage ist, zu telefonieren, müssen Ärzte eine gesetzliche Betreuung in die Wege leiten. Das wollen sie oft nicht machen
- Beim Aufnahmegespräch soll Assessment durchgeführt werden, das macht aber niemand
- Als zuständiger Arzt ist meist Stationsarzt eingetragen, TN muss erst herausfinden, wer der zuständige Arzt ist
- Wenn TN Patienten auffordert, einen Pflegeantrag auszufüllen, tun das nur zwei von zehn. Die anderen acht möchten, dass die TN es macht
- Im Kern entsteht ein Problem daraus, dass Patienten vor Eingriffen nicht umfangreich aufgeklärt/beraten werden
- Zuständigkeit des SD ist nach Organisationseinheit geregelt
- SD hat keine festen zuständigen Ansprechpartner im Bereich der Ärzte. Verantwortlichkeiten wechseln mitunter sehr schnell. Ärzte verweisen auf andere Zuständigkeit, Prozess des EM wird dadurch insgesamt negativ beeinflusst
- Kommunikation mit Kostenträgern herausfordrend. Konkrete Ansprechpartner sind nicht leicht ausfindig zu machen. Nicht ausreichend besetzte Hotlines erschweren die Recherche
- i.d.R. kein fester Kontakt bei KK für SD
- Oft kommt es dazu, dass die Anforderungen an den falschen Ansprechpartner gesandt werden. Das liegt daran, dass es mehrere Sozialarbeiter gibt. Manche gehören fest zu Organisationseinheiten.
- Wer letztlich wann entlassen wird, entscheidet der Oberarzt
- Welche Akteure im engeren Kontakt mit dem EM stehen, hängt von der jeweiligen Abteilung ab, für die der Prozess durchgeführt wird.
- Patientenaufnahme ist zuständig dafür, Informationen zum Patienten zu erheben und dokumentieren

## Unterkategorie Interdisziplinäre Zusammenarbeit

### Aussagen nach Workshops

#### Workshop 1

- Patienten werden von med. Personal umfangreich bei diversen Nachversorgern angemeldet, um eigene Pflicht zu erfüllen
- Bedarf und pers. Situation des Patienten werden nicht durch med. P. erhoben, erst durch SD
- Wenn Anmeldung bei SD erfolgt, betrachtet med. P. die Angelegenheit als erledigt/übertragen
- Geringer Bedarf der Nachversorgung wird durch med. P. als wenig herausfordernd fürs EM betrachtet
- Regelmäßig Beratung/Aufklärung von Patient:innen und Angehörige aufgrund falsch geplanter Nachversorgung durch Ärzte
- Anmeldungen zum EM bleiben oft aus
- Anmeldungen zum EM erfolgen oft zu spät
- Großes Arbeitsaufkommen durch (manuelle) Bearbeitung der Anrufe

- EM nimmt Kontakt zu Ärzten und Pflege auf um Entlasstag zu erfragen und Ersteinschätzung zu erhalten. Barthel-Index ist sehr wichtig
- Kommunikationsmedien sind Telefon, persönlich, Mail und Fax
- Patienten können Termine bei SD ausmachen
- Patienten können Termin ausmachen.
- Patienten kommen unangekündigt bei SD vorbei
- Ambulanz schickt Pat. Ohne Termin zu SD. Diese blockieren dann Ressourcen und lassen vergebene Termine platze
- Bei planbaren Eingriffen kontaktiert SD Patienten im Vorfeld und klärt Antragstellung ab
- Planung vorab bei akuten Erkrankungen nicht möglich
- MA kennen zuständige SD und sind personenfixiert
- Pat. Wird für Nachsorge angemeldet, die nicht gebraucht wird, weil sie schon besteht
- SD erfährt nicht automatisch von Station, dass Pat. Verstorben ist. Bekommt Info tlw. Von Angehörigen
- Es besteht ein pflegerische und ärztl. Standard. Für EM benötigter Input durch andere Berufsgruppen ist größtenteils nicht gegeben, sodass EM Prozessablauf ändern und fehlende Prozessschritte kompensieren muss
- Patienten konnten früher mit Hol- oder Bringdienst zu SD gelangen. Aufgrund von Personalmangel geht das nicht mehr
- Barthel-Index wird meist nicht ohne Aufforderung erhoben, obwohl er Pflicht ist
- Dokumentation wird meist nicht gemacht
- Pflegegrad wird nicht dokumentiert, obwohl oft angesprochen. Sollte spätestens bei Aufnahme auf Station dokumentiert sein
- SD erfährt nicht automatisch von Station, dass Pat. Verstorben ist. Bekommt Info tlw. Von Angehörigen
- Ärzte informieren SD nicht, wenn Pat. Verstirbt oder verlegt wird
- Relevante Eingabefelder innerhalb der KLAU werden regelm. Mit Platzhaltern gefüllt. SD muss dann Informationen zur Kontaktaufnahme selber ausfindig machen. Kontaktieren sie dazu die Station, sind die MA dort genervt

**Workshop 2**

- Abhören des Abs tlw. Herausfordernd
- Unterschiedliche Arbeitsweise/Nutzung verschiedene Kommunikationskanäle durch MA
- Klinisches Personal sieht aus Sicht der TN den Prozess als Ganzes nicht. Es wäre keine Sensibilität dafür da, dass die Daten für die Entlassung das Patienten benötigt würden, sodass die Betten frei gemacht werden können. Diese Aufgabe wird ausschließlich bei SD/PÜ verortet
- Sinnvoll wäre aus ihrer Sicht, dass Akteurübergreifend digital gearbtet wird
- Unterschiedliche Arbeitsweise/Nutzung verschiedene Kommunikationskanäle durch MA
- Dokumentation wird oft nicht gelesen
- Klinisches Personal sieht aus Sicht der TN den Prozess als ganzes nicht. Es wäre keine Sensibilität dafür da, dass die Daten für die Entlassung das Patienten benötigt würden, sodass die Betten frei gemacht werden können. Diese Aufgabe wird ausschließlich bei SD/PÜ verortet

- Mehrfachanmeldungen für den KLAU sind nervig. Sie entstehen, weil SD Befunde des Arztes braucht, um Anmeldung bei Nachversorger zu realisieren. Ärzte lesen aber die entsprechende Dokumentation des Pat. Nicht. Er oder die Pflege denken, dass der SD den EM nicht nachkommt und eröffnen einen neuen Auftrag.
- Herausforderungen beim KLAU sind, dass Patientendaten nicht vollständig oder inkorrekt sind oder dass unsinnige bzw. unklare Aufträge erteilt werden
- Mehrfachanmeldung zum EM infolge von Verlegung des Patienten
- Dokumentation im System ist teilweise sehr undurchsichtig. Einträge des SD werden teilweise durch MA unter älteren Einträgen fortgeführt. Andere legen neue Dokumente für aktuelles EM an. Besonders bei Patienten, die mehrere Aufenthalte habe, kann das zu Verwirrung führen
- Bei Anliegen in Zusammenhang mit IT geben die zuständigen Kollegen keine zuverlässige Auskunft bzw. Hilfestellung. Man muss wissen, welcher Ansprechpartner Kompetenzen im jeweiligen Bereich hat.
- Ärzte füllen notwendige Unterlagen spät, falsch oder gar nicht aus.
- Ärzte behaupten manchmal, dass Nachrichten oder Dokumente sie nicht erreicht haben oder ihre Nachricht abgesandt wurde, obwohl sie nie beim SD ankam.
- Information von Pat. Und Station erfolgt per Mail oder Telefon
- Wenn in der Cirurgie keine Tagesvisite durch SD gemacht wird, werden Patienten (durch die Ärzte?) gar nicht angemeldet. SD muss proaktiv auf Ärzte zugehen, damit Pat. Nachsorge vermittelt bekommt
- Vor Entlassung wird dem Patienten oder dessen Angehörigen eine Mail mit Information zum Entlassungsprozess gesendet. Damit werden sie auf wichtige Aspekte zur Vorbereitung hingewiesen
- Ärzte behaupten manchmal, dass Nachrichten oder Dokumente sie nicht erreicht haben oder ihre Nachricht abgesandt wurde, obwohl sie nie beim SD ankam.
- Rezepte werden oft falsch ausgestellt. Ärzte wissen oft nicht, dass nur ein Hilfsmittel pro Rezept von Sanitätshäusern akzeptiert wird. Eine KI könnte hier auf diesen Fehler im Erstellungsprozess hinweisen
- Bedarf des EM/der Nachsorge muss auf Grundlage der Anamnese angezeigt werden. In diesem Kontext muss die SD-MA in den persönlichen Dialog mit den Ärzten gehen und jeden Pat. der Abteilung mit ihm besprechen, um einen Bedarf zu ermitteln und aktiv werden zu können.
- Bei manchen KK erfährt man garnicht, ob die Kostenzusage erfolgt, andere müssen erneut telefonisch kontaktiert werden
- Wenn Kostenzusage nicht da ist, wird entweder Reha-Termin verschoben oder Reha ruft nochmal an

**Workshop 3**

- Weder Ärzte noch Pflege machen Anamnese, obwohl diese Standard ist. TN muss dann selbst alle Informationen erheben.
- Klinisches Personal gibt regelmäßig an, dass Pat. nicht befragt werden kann, obwohl er adäquat ist.
- Patienten werden wahllos zu allen möglichen Nachsorgemöglichkeiten angemeldet. Ein konkreter Bedarf wird nicht ermittelt, das wird vom EM verlangt

- Tafelvisite ist sehr zeitintensiv
- Alle Einrichtungen arbeiten mit Barthel-Index. Pflegekräften ist nicht bewusst, wie wichtig er ist.
- Andere Berufsgruppen interessieren sich nicht wirklich für Arbeitsweise des SD. Sie denken, sobald der Patient angemeldet ist, sei der Prozess abgeschlossen. Sie verstehen nicht, dass SD dann erst beginnen kann, die Tätigkeit aufzunehmen und die Zusammenarbeit mit vielen Berufsgruppen erforderlich ist
- Dokumentation wird von klinischem Personal nicht gelesen.
- Informationen zu Kontaktdaten warden nicht eingetragen. Im Diagnosefeld wird auf den Arztbrief verwiesen. Das führt dazu, dass MA sehr viel recherchieren müssen
- Angehörige kontaktieren SD und fragen, warum dieser sich nicht gemeldet hat. Sie haben ihre Nummer in der Akte auf der Station hinterlegt. Diese wurde von den Pflegekräften nicht ins System übertragen
- TN muss erst herausfinden, wer der zuständige Arzt ist
- Station meldet sämtliche verfügbaren Optionen zur Nachsorge bei PÜ an. Diese weiß dann anhand der Anmeldung nicht, welcher Bedarf tatsächlich besteh
- Vorschläge zur Umsetzung der Bedarfsermittlung von Nachsorgern
- Oft neue Anmeldung bereits gemeldeter Patienten bei Schichtwechsel
- Wenn Patient selbst nicht in der Lage ist, zu telefonieren, müssen Ärzte eine gesetzliche Betreuung in die Wege leiten. Das wollen sie oft nicht machen
- Mobiltelefon funktioniert auf der Station nicht, weil der Sendebereich unzureichend ist
- MA der PÜ hat sich Textbausteine für ärztliche Verordnungen angelegt, damit sie Ärzten eine Vorlage zukommen lassen kann.
- Als zuständiger Arzt ist meist Stationsarzt eingetragen
- Pflege liest Dokumentation der PÜ nicht
- PÜ erhält keine Information, wenn ein Patient verlegt wurde. Muss diese Information täglich selbst suchen
- Eine Herausforderung ist, dass die PÜ/SD sich erstmal jedes angemeldeten Falls annehmen (müssen?). Durch die fehlende Bedarfsprüfung zu Beginn des Prozesses wird garnicht erhoben, welche Ressourcen der Patient und sein Umfeld innehaben. Würde die Einstellung seitens Krankenhaus als Institution von dieser Servicementatlität abrücken und die Patienten eher in die Selbstverantwortung nehmen, wäre das Arbeitsaufkommen um ein Vielfaches geringer

**Workshop 4**

- Station muss oft zum Erstellen des Barthel-Index aufgefordert werden
- Bei Fragen zu Patienten ist klinisches Personal für TN schwer erreichbar
- TN erfährt meist nur durch aktive Recherche in der Dokumentation von für sie relevanten Änderungen beim Patienten
- Bei Fragen zu Patienten ist klinisches Personal für TN schwer erreichbar
- In Notaufnahme werden fast nie Patientendaten dokumentiert
- Es kam in der Vergangenheit vor, dass Faxe nicht bei der Krankenkasse ankamen und eine Bewilligung in der Folge nicht rechtzeitig erfolgt

- Wenn TN keine Kontaktdaten von Angehörigen haben oder auffinden können, legen sie ihre Kontaktdaten ins Patientenzimmer und bitten um Anruf

**Workshop 5**

- SD ruft bei Kostenträgern an, um eine Beschleunigung in laufenden Verfahren zu erreichen. Tlw. Werden Kostenzusagen sehr dringend benötigt
- Zahl der Anrufe sehr hoch, erfordert viel Personalressourcen zur Koordinierung
- Ärzte haben Möglichkeit, dringende Anforderungen an den SD zu stellen.
- Möglichkeit der Markierung der hohen Dringlichkeit wird häufiger genutzt, als es nach Ansicht des SD erforderlich wäre
- Meiste Herausforderungen gibt es in der Zusammenarbeit mit Ärzten
- Herausforderung in der Interd. Zusammenarbeit. Professionen verfolgen eigene Ziele. Hier stehen sie in direktem Konflikt
- Führt dazu, dass falsche Auskünfte gegeben und Versprechen gemacht werden. Daraus resultieren Diskussionen zwischen Pat. Und SD
- Patienten werden oft entlassen, weil deren Pflegebett benötigt wird. Dies geschieht, ohne das mit zuständiger SD-MA abzusprechen. Diese kann ihre Beratung nicht vollständig durchführen.
- Großes Problem ist Erreichbarkeit von Ärzten
- Kommunikation mit Kostenträgern herausfordrend. Konkrete Ansprechpartner sind nicht leicht ausfindig zu machen. Nicht ausreichend besetzte Hotlines erschweren die Recherche
- i.d.R. kein fester Kontakt bei KK für SD
- Klinikinterne Prozesse sind nicht aufeinander abgestimmt. Prozess der OP bedarf eigentlich der Abstimmung mit Prozess des EM, wird aber nicht vorgenommen. So kommt es Herausforderungen bei der Nachsorge und Versorgungsbruch.
- Im Kern entsteht ein Problem daraus, dass Patienten vor Eingriffen nicht umfangreich aufgeklärt/beraten werden
- Resultat des oben beschriebenen Vorgehens ist, dass Pat. Sich einer geplanten OP unterziehen und danach verwundert sind, dass nicht sofort eine Nachsorge gewährleistet ist
- Problem ist oft, dass EM geplant ist, der Patient dann aber verlegt wird und SD darüber nicht informiert wird. KI wäre hier hilfreich
- Wenn unzureichende Informationen zum Pat. vorliegen, können Mitarbeitende nicht einschätzen, welche Profession tätig werden muss
- EM wird oft nicht über Verlegung des Patienten informiert und muss ihn dann suchen
- Problem ist, dass Nachversorger nicht die gleiche Software nutzen. Über einzelne Kontaktanfragen per Mail oder Telefon geben sie zuverlässig Auskunft
- SD wird nicht regelmäßig über Änderungen der OP bei Pat. Informiert. Wünscht sich automatische Information, damit Nachversorgung angepasst werden kann
- Mit Rehas wird hier nicht zusammengearbeitet
- Kommunikation mit den Ärzten wird als unzureichend empfunden. Absprachen werden manchmal nicht eingehalten

- Fax ist der vorrangige Kommunikationsweg zwischen den Einrichtungen
- SD macht mit Angehörigen Termin aus, wenn diese in den Prozess involviert werden müssen
- Bedarfserhebung umfasst auch Rücksprache mit anderen Disziplinen
- SD muss oft um Erstellung der ärztlichen Befunde bitten
- Im Bereich der Reha-Vermittlung kommt es oft vor, dass Formulare vom EM an Ärzte zur Bearbeitung gesendet werden, die dann aber nur teilweise bis gar nicht ausgefüllt oder falsch bearbeitet sind. Oft muss auch mehrmals auf die Bearbeitung hingewiesen werden, bis sie vorgenommen wird. Angesprochene Ärzte sind oft nicht in der Zuständigkeit

## Gesamtdarstellung aller Punkte

- Patienten werden von med. Personal umfangreich bei diversen Nachversorgern angemeldet, um eigene Pflicht zu erfüllen
- Bedarf und pers. Situation des Patienten werden nicht durch med. P. erhoben, erst durch SD
- Wenn Anmeldung bei SD erfolgt, betrachtet med. P. die Angelegenheit als erledigt/übertragen
- Geringer Bedarf der Nachversorgung wird durch med. P. als wenig herausfordernd fürs EM betrachtet
- Regelmäßig Beratung/Aufklärung von Patient:innen und Angehörige aufgrund falsch geplanter Nachversorgung durch Ärzte
- Anmeldungen zum EM bleiben oft aus
- Anmeldungen zum EM erfolgen oft zu spät
- Großes Arbeitsaufkommen durch (manuelle) Bearbeitung der Anrufe
- EM nimmt Kontakt zu Ärzten und Pflege auf um Entlasstag zu erfragen und Ersteinschätzung zu erhalten. Barthel-Index ist sehr wichtig
- Kommunikationsmedien sind Telefon, persönlich, Mail und Fax
- Patienten können Termine bei SD ausmachen
- Patienten können Termin ausmachen.
- Patienten kommen unangekündigt bei SD vorbei
- Ambulanz schickt Pat. Ohne Termin zu SD. Diese blockieren dann Ressourcen und lassen vergebene Termine platze
- Bei planbaren Eingriffen kontaktiert SD Patienten im Vorfeld und klärt Antragstellung ab
- Planung vorab bei akuten Erkrankungen nicht möglich
- MA kennen zuständige SD und sind personenfixiert
- Pat. Wird für Nachsorge angemeldet, die nicht gebraucht wird, weil sie schon besteht
- SD erfährt nicht automatisch von Station, dass Pat. Verstorben ist. Bekommt Info tlw. Von Angehörigen
- Es besteht ein pflegerische und ärztl. Standard. Für EM benötigter Input durch andere Berufsgruppen ist größtenteils nicht gegeben, sodass EM Prozessablauf ändern und fehlende Prozessschritte kompensieren muss
- Patienten konnten früher mit Hol- oder Bringdienst zu SD gelangen. Aufgrund von Personalmangel geht das nicht mehr
- Barthel-Index wird meist nicht ohne Aufforderung erhoben, obwohl er Pflicht ist
- Dokumentation wird meist nicht gemacht

- Pflegegrad wird nicht dokumentiert, obwohl oft angesprochen. Sollte spätestens bei Aufnahme auf Station dokumentiert sein
- SD erfährt nicht automatisch von Station, dass Pat. Verstorben ist. Bekommt Info tlw. Von Angehörigen
- Ärzte informieren SD nicht, wenn Pat. Verstirbt oder verlegt wird
- Relevante Eingabefelder innerhalb der KLAU werden regelm. Mit Platzhaltern gefüllt. SD muss dann Informationen zur Kontaktaufnahme selber ausfindig machen. Kontaktieren sie dazu die Station, sind die MA dort genervt
- Abhören des Abs tlw. Herausfordernd
- Unterschiedliche Arbeitsweise/Nutzung verschiedene Kommunikationskanäle durch MA
- Klinisches Personal sieht aus Sicht der TN den Prozess als Ganzes nicht. Es wäre keine Sensibilität dafür da, dass die Daten für die Entlassung das Patienten benötigt würden, sodass die Betten frei gemacht werden können. Diese Aufgabe wird ausschließlich bei SD/PÜ verortet
- Sinnvoll wäre aus ihrer Sicht, dass Akteurübergreifend digital gearbtet wird
- Unterschiedliche Arbeitsweise/Nutzung verschiedene Kommunikationskanäle durch MA
- Dokumentation wird oft nicht gelesen
- Klinisches Personal sieht aus Sicht der TN den Prozess als ganzes nicht. Es wäre keine Sensibilität dafür da, dass die Daten für die Entlassung das Patienten benötigt würden, sodass die Betten frei gemacht werden können. Diese Aufgabe wird ausschließlich bei SD/PÜ verortet
- Mehrfachanmeldungen für den KLAU sind nervig. Sie entstehen, weil SD Befunde des Arztes braucht, um Anmeldung bei Nachversorger zu realisieren. Ärzte lesen aber die entsprechende Dokumentation des Pat. Nicht. Er oder die Pflege denken, dass der SD den EM nicht nachkommt und eröffnen einen neuen Auftrag.
- Herausforderungen beim KLAU sind, dass Patientendaten nicht vollständig oder inkorrekt sind oder dass unsinnige bzw. unklare Aufträge erteilt werden
- Mehrfachanmeldung zum EM infolge von Verlegung des Patienten
- Dokumentation im System ist teilweise sehr undurchsichtig. Einträge des SD werden teilweise durch MA unter älteren Einträgen fortgeführt. Andere legen neue Dokumente für aktuelles EM an. Besonders bei Patienten, die mehrere Aufenthalte habe, kann das zu Verwirrung führen
- Bei Anliegen in Zusammenhang mit IT geben die zuständigen Kollegen keine zuverlässige Auskunft bzw. Hilfestellung. Man muss wissen, welcher Ansprechpartner Kompetenzen im jeweiligen Bereich hat.
- Ärzte füllen notwendige Unterlagen spät, falsch oder gar nicht aus.
- Ärzte behaupten manchmal, dass Nachrichten oder Dokumente sie nicht erreicht haben oder ihre Nachricht abgesandt wurde, obwohl sie nie beim SD ankam.
- Information von Pat. Und Station erfolgt per Mail oder Telefon
- Wenn in der Cirurgie keine Tagesvisite durch SD gemacht wird, werden Patienten (durch die Ärzte?) gar nicht angemeldet. SD muss proaktiv auf Ärzte zugehen, damit Pat. Nachsorge vermittelt bekommt
- Vor Entlassung wird dem Patienten oder dessen Angehörigen eine Mail mit Information zum Entlassungsprozess gesendet. Damit werden sie auf wichtige Aspekte zur Vorbereitung hingewiesen

- Ärzte behaupten manchmal, dass Nachrichten oder Dokumente sie nicht erreicht haben oder ihre Nachricht abgesandt wurde, obwohl sie nie beim SD ankam.
- Rezepte werden oft falsch ausgestellt. Ärzte wissen oft nicht, dass nur ein Hilfsmittel pro Rezept von Sanitätshäusern akzeptiert wird. Eine KI könnte hier auf diesen Fehler im Erstellungsprozess hinweisen
- Bedarf des EM/der Nachsorge muss auf Grundlage der Anamnese angezeigt werden. In diesem Kontext muss die SD-MA in den persönlichen Dialog mit den Ärzten gehen und jeden Pat. der Abteilung mit ihm besprechen, um einen Bedarf zu ermitteln und aktiv werden zu können.
- Bei manchen KK erfährt man garnicht, ob die Kostenzusage erfolgt, andere müssen erneut telefonisch kontaktiert werden
- Wenn Kostenzusage nicht da ist, wird entweder Reha-Termin verschoben oder Reha ruft nochmal an
- Weder Ärzte noch Pflege machen Anamnese, obwohl diese Standard ist. TN muss dann selbst alle Informationen erheben.
- Klinisches Personal gibt regelmäßig an, dass Pat. nicht befragt werden kann, obwohl er adäquat ist.
- Patienten werden wahllos zu allen möglichen Nachsorgemöglichkeiten angemeldet. Ein konkreter Bedarf wird nicht ermittelt, das wird vom EM verlangt
- Tafelvisite ist sehr zeitintensiv
- Alle Einrichtungen arbeiten mit Barthel-Index. Pflegekräften ist nicht bewusst, wie wichtig er ist.
- Andere Berufsgruppen interessieren sich nicht wirklich für Arbeitsweise des SD. Sie denken, sobald der Patient angemeldet ist, sei der Prozess abgeschlossen. Sie verstehen nicht, dass SD dann erst beginnen kann, die Tätigkeit aufzunehmen und die Zusammenarbeit mit vielen Berufsgruppen erforderlich ist
- Dokumentation wird von klinischem Personal nicht gelesen.
- Informationen zu Kontaktdaten warden nicht eingetragen. Im Diagnosefeld wird auf den Arztbrief verwiesen. Das führt dazu, dass MA sehr viel recherchieren müssen
- Angehörige kontaktieren SD und fragen, warum dieser sich nicht gemeldet hat. Sie haben ihre Nummer in der Akte auf der Station hinterlegt. Diese wurde von den Pflegekräften nicht ins System übertragen
- TN muss erst herausfinden, wer der zuständige Arzt ist
- Station meldet sämtliche verfügbaren Optionen zur Nachsorge bei PÜ an. Diese weiß dann anhand der Anmeldung nicht, welcher Bedarf tatsächlich besteh
- Vorschläge zur Umsetzung der Bedarfsermittlung von Nachsorgern
- Oft neue Anmeldung bereits gemeldeter Patienten bei Schichtwechsel
- Wenn Patient selbst nicht in der Lage ist, zu telefonieren, müssen Ärzte eine gesetzliche Betreuung in die Wege leiten. Das wollen sie oft nicht machen
- Mobiltelefon funktioniert auf der Station nicht, weil der Sendebereich unzureichend ist
- MA der PÜ hat sich Textbausteine für ärztliche Verordnungen angelegt, damit sie Ärzten eine Vorlage zukommen lassen kann.
- Als zuständiger Arzt ist meist Stationsarzt eingetragen

- Pflege liest Dokumentation der PÜ nicht
- PÜ erhält keine Information, wenn ein Patient verlegt wurde. Muss diese Information täglich selbst suchen
- Eine Herausforderung ist, dass die PÜ/SD sich erstmal jedes angemeldeten Falls annehmen (müssen?). Durch die fehlende Bedarfsprüfung zu Beginn des Prozesses wird garnicht erhoben, welche Ressourcen der Patient und sein Umfeld innehaben. Würde die Einstellung seitens Krankenhaus als Institution von dieser Servicementatlität abrücken und die Patienten eher in die Selbstverantwortung nehmen, wäre das Arbeitsaufkommen um ei Vielfaches geringer
- Station muss oft zum Erstellen des Barthel-Index aufgefordert werden
- Bei Fragen zu Patienten ist klinisches Personal für TN schwer erreichbar
- TN erfährt meist nur durch aktive Recherche in der Dokumentation von für sie relevanten Änderungen beim Patienten
- Bei Fragen zu Patienten ist klinisches Personal für TN schwer erreichbar
- In Notaufnahme werden fast nie Patientendaten dokumentiert
- Es kam in der Vergangenheit vor, dass Faxe nicht bei der Krankenkasse ankamen und eine Bewilligung in der Folge nicht rechtzeitig erfolgt
- Wenn TN keine Kontaktdaten von Angehörigen haben oder auffinden können, legen sie ihre Kontaktdaten ins Patientenzimmer und bitten um Anruf
- SD ruft bei Kostenträgern an, um eine Beschleunigung in laufenden Verfahren zu erreichen. Tlw. Werden Kostenzusagen sehr dringend benötigt
- Zahl der Anrufe sehr hoch, erfordert viel Personalressourcen zur Koordinierung
- Ärzte haben Möglichkeit, dringende Anforderungen an den SD zu stellen.
- Möglichkeit der Markierung der hohen Dringlichkeit wird häufiger genutzt, als es nach Ansicht des SD erforderlich wäre
- Meiste Herausforderungen gibt es in der Zusammenarbeit mit Ärzten
- Herausforderung in der Interd. Zusammenarbeit. Professionen verfolgen eigene Ziele. Hier stehen sie in direktem Konflikt
- Führt dazu, dass falsche Auskünfte gegeben und Versprechen gemacht werden. Daraus resultieren Diskussionen zwischen Pat. Und SD
- Patienten werden oft entlassen, weil deren Pflegebett benötigt wird. Dies geschieht, ohne das mit zuständiger SD-MA abzusprechen. Diese kann ihre Beratung nicht vollständig durchführen.
- Großes Problem ist Erreichbarkeit von Ärzten
- Kommunikation mit Kostenträgern herausfordrend. Konkrete Ansprechpartner sind nicht leicht ausfindig zu machen. Nicht ausreichend besetzte Hotlines erschweren die Recherche
- i.d.R. kein fester Kontakt bei KK für SD
- Klinikinterne Prozesse sind nicht aufeinander abgestimmt. Prozess der OP bedarf eigentlich der Abstimmung mit Prozess des EM, wird aber nicht vorgenommen. So kommt es Herausforderungen bei der Nachsorge und Versorgungsbruch.
- Im Kern entsteht ein Problem daraus, dass Patienten vor Eingriffen nicht umfangreich aufgeklärt/beraten werden
- Resultat des oben beschriebenen Vorgehens ist, dass Pat. Sich einer geplanten OP unterziehen und danach verwundert sind, dass nicht sofort eine Nachsorge gewährleistet ist

- Problem ist oft, dass EM geplant ist, der Patient dann aber verlegt wird und SD darüber nicht informiert wird. KI wäre hier hilfreich
- Wenn unzureichende Informationen zum Pat. vorliegen, können Mitarbeitende nicht einschätzen, welche Profession tätig werden muss
- EM wird oft nicht über Verlegung des Patienten informiert und muss ihn dann suchen
- Problem ist, dass Nachversorger nicht die gleiche Software nutzen. Über einzelne Kontaktanfragen per Mail oder Telefon geben sie zuverlässig Auskunft
- SD wird nicht regelmäßig über Änderungen der OP bei Pat. Informiert. Wünscht sich automatische Information, damit Nachversorgung angepasst werden kann
- Mit Rehas wird hier nicht zusammengearbeitet
- Kommunikation mit den Ärzten wird als unzureichend empfunden. Absprachen werden manchmal nicht eingehalten
- Fax ist der vorrangige Kommunikationsweg zwischen den Einrichtungen
- SD macht mit Angehörigen Termin aus, wenn diese in den Prozess involviert werden müssen
- Bedarfserhebung umfasst auch Rücksprache mit anderen Disziplinen
- SD muss oft um Erstellung der ärztlichen Befunde bitten
- Im Bereich der Reha-Vermittlung kommt es oft vor, dass Formulare vom EM an Ärzte zur Bearbeitung gesendet werden, die dann aber nur teilweise bis gar nicht ausgefüllt oder falsch bearbeitet sind. Oft muss auch mehrmals auf die Bearbeitung hingewiesen werden, bis sie vorgenommen wird. Angesprochene Ärzte sind oft nicht in der Zuständigkeit

## Kategorie Patientenzentrierte Versorgungsbedarfe

### Unterkategorie Medizinische und pflegerische Bedarfe

#### Aussagen nach Workshops

**Workshop 1**

- Pfleg. Einschätzung als Assessment nicht ausreichend für eine vollständige Darstellung des Zustands
- Teilweise sind häusl./persönl. Situationen der Patienten herausfordernd, sodass ethische Bedenken seitens der Pflege bestehen, ob dieser Pat. Entlassen werden kann. Aus rechtl. Aspekt steht der Entlassung nichts im Wege. Spannungsfeld für SD
- Nicht jeder Patient hat Wunsch nach EM
- Zu Beginn benötigt SD Information über kognitiven Status des Pat. Und etwaige Betreuer
- Tatsächlicher Hilfebedarf einer Person fällt insbesondere bei Patienten für Geriatrie erst im Verlauf auf
- Einschätzung der Ressourcen der Patient:innen bereits früh möglich (Reha-Fähgkeit)
- Erstkontakt dient der Bedarfserhebung
- Verweildauer ist abhängig von Erkrankung
- Verweildauer mancher Patienten teilweise Jahre. Kommen ein bis zwei mal pro Monat. Bei ihnen wir Zeit abgekappt.

- Barthel-Index wird benötigt, um Verlauf der Krankheit/Genesung zu ermitteln. Dient auch als Argument für Kostenübernahme durch KK
- SD muss sich selbst vor Ort ein Bild des Patienten machen
- Ist Pflegebedarf erhoben, wird persönlich mit Pat./Angehörigen gesprochen und Setting erfragt
- Wenn Pflegedienst als Nachsorger eintritt, müssen benötigte Hilfsmittel ermittelt und beschafft werden
- Teilweise sind häusl./persönl. Situationen der Patienten herausfordernd, sodass ethische Bedenken seitens der Pflege bestehen, ob dieser Pat. Entlassen werden kann. Aus rechtl. Aspekt steht der Entlassung nichts im Wege. Spannungsfeld für SD
- Pat. Wird für Nachsorge angemeldet, die nicht gebraucht wird, weil sie schon besteht
- Pat. Sieht keinen Beratungsbedarf, wenn von ärztl. Seite kommuniziert wird, das der Eingriff erfolgreich war
- Negative Vorerfahrung der SD-MA, weil Pat. Nicht von Arzt erfuhr, dass er Palliativstatus hat.

**Workshop 2**

- Bei onkologischen Patienten in der Chirurgie findet die Besprechung erst nach der Entlassung statt.

**Workshop 3**

- Eine Herausforderung ist, dass die PÜ/SD sich erstmal jedes angemeldeten Falls annehmen (müssen?). Durch die fehlende Bedarfsprüfung zu Beginn des Prozesses wird garnicht erhoben, welche Ressourcen der Patient und sein Umfeld innehaben. Würde die Einstellung seitens Krankenhaus als Institution von dieser Servicementatlität abrücken und die Patienten eher in die Selbstverantwortung nehmen, wäre das Arbeitsaufkommen um ei Vielfaches geringer

**Workshop 4**

- Betreuungsverfahren als Folge von geschäftsunfähigkeit und fehlender Betreuungsverfügung
- Manchmal wird erst kurz vor der Entlassung festgestellt, dass ein Patient nicht geschäftstüchtig ist.

**Workshop 5**

- Häufig können Patienten ihre eigenen Bedarfe nicht nennen. SD nimmt dann Kontakt zu Angehörigen auf, um mit diesen die Bedarfe zu besprechen. Dies geschieht tlw. Gemeinsam mit Pat.
- Resultat des oben beschriebenen Vorgehens ist, dass Pat. Sich einer geplanten OP unterziehen und danach verwundert sind, dass nicht sofort eine Nachsorge gewährleistet ist
- Bedeutung der beteiligte Akteure hängt von Bedarf der Patienten ab
- Bei bestimmten Krankheitsbildern oder Nachsorgebedrafen kommt es vor, dass SD große Schwierigkeiten hat, einen geeigneten Nachversorger mit Aufnahmekapazität zu finden. Dann wenden se sich an Kostenträger mit Bitte

um Unterstützung. Es wäre eine Erleichterung, wenn KI bei solchen Verfahren automatisiert eine

- Patient muss im Zweifel nach Hause, wenn er keinen Nachversorger hat
- Wenn MA keine Kenntnis über demenz. Erkrankung eines Pat. Haben verlängert und erschwert das den Prozess

**Workshop 6:**

- Nächster Prozessschritt ist das Ermitteln des Bedarfes. Das erfolgt oft auch gemeinsam mit Angehörigen
- Zur Ermittlung des Bedarfs gehört, je nach Abteilung, auch die Rücksprache mit den behandelnden Ärzten. Im Idealfall sind diese Informationen schon mit in der Anforderung hinterlegt

## Gesamtdarstellung aller Punkte

- Pfleg. Einschätzung als Assessment nicht ausreichend für eine vollständige Darstellung des Zustands
- Teilweise sind häusl./persönl. Situationen der Patienten herausfordernd, sodass ethische Bedenken seitens der Pflege bestehen, ob dieser Pat. Entlassen werden kann. Aus rechtl. Aspekt steht der Entlassung nichts im Wege. Spannungsfeld für SD
- Nicht jeder Patient hat Wunsch nach EM
- Zu Beginn benötigt SD Information über kognitiven Status des Pat. Und etwaige Betreuer
- Tatsächlicher Hilfebedarf einer Person fällt insbesondere bei Patienten für Geriatrie erst im Verlauf auf
- Einschätzung der Ressourcen der Patient:innen bereits früh möglich (Reha-Fähgkeit)
- Erstkontakt dient der Bedarfserhebung
- Verweildauer ist abhängig von Erkrankung
- Verweildauer mancher Patienten teilweise Jahre. Kommen ein bis zwei mal pro Monat. Bei ihnen wir Zeit abgekappt.
- Barthel-Index wird benötigt, um Verlauf der Krankheit/Genesung zu ermitteln. Dient auch als Argument für Kostenübernahme durch KK
- SD muss sich selbst vor Ort ein Bild des Patienten machen
- Ist Pflegebedarf erhoben, wird persönlich mit Pat./Angehörigen gesprochen und Setting erfragt
- Wenn Pflegedienst als Nachsorger eintritt, müssen benötigte Hilfsmittel ermittelt und beschafft werden
- Teilweise sind häusl./persönl. Situationen der Patienten herausfordernd, sodass ethische Bedenken seitens der Pflege bestehen, ob dieser Pat. Entlassen werden kann. Aus rechtl. Aspekt steht der Entlassung nichts im Wege. Spannungsfeld für SD
- Pat. Wird für Nachsorge angemeldet, die nicht gebraucht wird, weil sie schon besteht
- Pat. Sieht keinen Beratungsbedarf, wenn von ärztl. Seite kommuniziert wird, das der Eingriff erfolgreich war
- Negative Vorerfahrung der SD-MA, weil Pat. Nicht von Arzt erfuhr, dass er Palliativstatus hat.

- Bei onkologischen Patienten in der Chirurgie findet die Besprechung erst nach der Entlassung statt.
- Eine Herausforderung ist, dass die PÜ/SD sich erstmal jedes angemeldeten Falls annehmen (müssen?). Durch die fehlende Bedarfsprüfung zu Beginn des Prozesses wird garnicht erhoben, welche Ressourcen der Patient und sein Umfeld innehaben. Würde die Einstellung seitens Krankenhaus als Institution von dieser Servicementatlität abrücken und die Patienten eher in die Selbstverantwortung nehmen, wäre das Arbeitsaufkommen um ei Vielfaches geringer
- Betreuungsverfahren als Folge von geschäftsunfähigkeit und fehlender Betreuungsverfügung
- Manchmal wird erst kurz vor der Entlassung festgestellt, dass ein Patient nicht geschäftstüchtig ist.
- Häufig können Patienten ihre eigenen Bedarfe nicht nennen. SD nimmt dann Kontakt zu Angehörigen auf, um mit diesen die Bedarfe zu besprechen. Dies geschieht tlw. Gemeinsam mit Pat.
- Resultat des oben beschriebenen Vorgehens ist, dass Pat. Sich einer geplanten OP unterziehen und danach verwundert sind, dass nicht sofort eine Nachsorge gewährleistet ist
- Bedeutung der beteiligte Akteure hängt von Bedarf der Patienten ab
- Bei bestimmten Krankheitsbildern oder Nachsorgebedrafen kommt es vor, dass SD große Schwierigkeiten hat, einen geeigneten Nachversorger mit Aufnahmekapazität zu finden. Dann wenden se sich an Kostenträger mit Bitte um Unterstützung. Es wäre eine Erleichterung, wenn KI bei solchen Verfahren automatisiert eine
- Patient muss im Zweifel nach Hause, wenn er keinen Nachversorger hat
- Wenn MA keine Kenntnis über demenz. Erkrankung eines Pat. Haben verlängert und erschwert das den Prozess
- Nächster Prozessschritt ist das Ermitteln des Bedarfes. Das erfolgt oft auch gemeinsam mit Angehörigen
- Zur Ermittlung des Bedarfs gehört, je nach Abteilung, auch die Rücksprache mit den behandelnden Ärzten. Im Idealfall sind diese Informationen schon mit in der Anforderung hinterlegt

## Unterkategorie Gesundheitskompetenz und Beratungsbedarf

### Aussagen nach Workshops

#### Workshop 1

- Beratungsgespräche sehr ressourcenintensiv
- Großes Arbeitsaufkommen durch (manuelle) Bearbeitung der Anrufe
- Viele Menschen setzen sich nicht mit der Thematik „Vorsorge“ auseinander. Ein/e langjährige/r Partner/in wird nicht automatisch als Betreuer/in anerkannt
- Ältere Personen befassen sich oft nicht mit dem Tod und Sterben
- Patienten haben unterschiedliche Bedarfe an Beratung
- SD muss sich selbst vor Ort ein Bild des Patienten machen
- Pat. Sieht keinen Beratungsbedarf, wenn von ärztl. Seite kommuniziert wird, das der Eingriff erfolgreich war

**Workshop 2**

- Immer wieder gestellte Fragen hemmen Arbeitsfortschritt des SD
- Komplexität und Anspruch an die Versorgung der Pat. Steigt seit Jahren. Diese werden im durchschnitt immer älter und morbider. Angehörige als Ressource (für Pat. + SD) entfällt zunehmend. Daher höhere Arbeitsaufwand pro Patient
- Bei onkologischen Patienten in der Chirurgie findet die Besprechung erst nach der Entlassung statt.

**Workshop 3**

- Eine der Pflegeüberleitung häufig gestellt Frage ist, wie ein Pflegegrad beantragt wird
- Eine de Pflegeüberleitung häufig gestellt Frage ist, wie jemand Unterstützung zu Hause erhalten kann
- Eine de Pflegeüberleitung häufig gestellt Frage ist, wie man an ein Rezept für ein Hilfsmittel gelangt
- Eine de Pflegeüberleitung häufig gestellt Frage ist, wie finanzielle Unterstützung erhalten werden kann
- Eine de Pflegeüberleitung häufig gestellt Frage ist, wie eine Reha beantragt werden kann
- Eine de Pflegeüberleitung häufig gestellt Frage ist, wie ein Grad der Behinderung beantragt wird
- Eine de Pflegeüberleitung häufig gestellt Frage ist, ob ein Anspruch geltend gemacht werden kann
- Eine de Pflegeüberleitung häufig gestellt Frage ist, wie eine vollmacht erteilt werden kann
- Allgemein wird fast immer beraten zu Möglichkeiten der Versorgung zu Hause, Leistungen der Kranken- und Pflegeversicherung, Pflegegrad, Hilfsmittelversorgung, Hausnotrufsysteme und Hilfe zur Pflege

**Workshop 4**

- Wenn TN Patienten auffordert, einen Pflegeantrag auszufüllen, tun das nur zwei von zehn. Die anderen acht möchten, dass die TN es macht
- Mache Patienten/Angehörige wissen nicht, dass sie einen Pflegegrad selber beantragen können. Sie hinterlegen das Dokument beim Stationsarzt und warten darauf, dass dieser auf sie zukommt
- Beratung zu Versorgungsleistungen muss auf jeweilige Person und Situation abgestimmt sein. Faktoren wie Art der Versicherung, gewünschtes Pflegesetting und vorhandene Ressourcen bestimmen den Prozess maßgeblich
- Mache Patienten/Angehörige wissen nicht, dass sie einen Pflegegrad selber beantragen können. Sie hinterlegen das Dokument beim Stationsarzt und warten darauf, dass dieser auf sie zukommt
- Patienten, die zu geplanten OPs kommen, stellen erst nach dem Eingriff fest, dass sie Hilfe bei der Haushaltsführung benötigen. Ein Antrag dafür kann auch vor der OP aufgesetzt und bei der Krankenkasse eingereicht werden. Dann ist die Nachsorge auch zum Entlassungszeitpunkt abgesichert
- Menschen beschäftigen sich mit Pflegebedürftigkeit und Vorsorge nicht, solange sie nicht unmittelbar davon betroffen sind

- Infolge eines Informationsdefizits beziehen die Personen u.a. Leistungen von Sanitätshäusern, bei denen die Kosten nicht von der Krankenkasse übernommen werden

**Workshop 5**

- SD berät und empfiehlt Patienten zu der Versorgungsform, die er bedarf und die auch faktisch umsetzbar ist
- Ärzte versprechen Pat. Dinge, die gesetzlich nicht bestand haben
- Ärzte führen eine vermeintliche Beratung der Patienten durch bzw. stellen diesen Versorgungsmaßnahmen in Aussicht, die so nicht umsetzbar sind
- Im Kern entsteht ein Problem daraus, dass Patienten vor Eingriffen nicht umfangreich aufgeklärt/beraten werden
- Resultat des oben beschriebenen Vorgehens ist, dass Pat. Sich einer geplanten OP unterziehen und danach verwundert sind, dass nicht sofort eine Nachsorge gewährleistet ist
- Häufig können Patienten ihre eigenen Bedarfe nicht nennen. SD nimmt dann Kontakt zu Angehörigen auf, um mit diesen die Bedarfe zu besprechen. Dies geschieht tlw. Gemeinsam mit Pat.

## Gesamtdarstellung aller Punkte

- Beratungsgespräche sehr ressourcenintensiv, Beratung zu Versorgungsleistungen muss auf jeweilige Person und Situation abgestimmt sein. Faktoren wie Art der Versicherung, gewünschtes Pflegesetting und vorhandene Ressourcen bestimmen den Prozess maßgeblich
- Großes Arbeitsaufkommen durch (manuelle) Bearbeitung der Anrufe. Dabei hemmen insbesondere Immer wieder gestellte Fragen den Arbeitsfortschritt des SD
- Viele Menschen setzen sich nicht mit der Thematik „Vorsorge“ auseinander. Ein/e langjährige/r Partner/in wird nicht automatisch als Betreuer/in anerkannt, insbesondere ältere Personen befassen sich selten mit dem Tod und Sterben
- Patienten haben unterschiedliche Bedarfe an Beratung
- SD muss sich selbst vor Ort ein Bild des Patienten machen, um vorhandene Ressourcen und Bedarfe zu erfassen
- Pat. Sieht keinen Beratungsbedarf, wenn von ärztl. Seite kommuniziert wird, das der Eingriff erfolgreich war
- Komplexität und Anspruch an die Versorgung der Pat. Steigt seit Jahren. Diese werden im durchschnitt immer älter und morbider. Angehörige als Ressource (für Pat. + SD) entfällt zunehmend. Daher höhere Arbeitsaufwand pro Patient
- Bei onkologischen Patienten in der Chirurgie findet die Besprechung erst nach der Entlassung statt.
- Eine der Pflegeüberleitung häufig gestellt Frage ist, wie ein Pflegegrad beantragt wird
- Eine de Pflegeüberleitung häufig gestellt Frage ist, wie jemand Unterstützung zu Hause erhalten kann

- Eine de Pflegeüberleitung häufig gestellt Frage ist, wie man an ein Rezept für ein Hilfsmittel gelangt
- Eine de Pflegeüberleitung häufig gestellt Frage ist, wie finanzielle Unterstützung erhalten werden kann
- Eine de Pflegeüberleitung häufig gestellt Frage ist, wie eine Reha beantragt werden kann
- Eine de Pflegeüberleitung häufig gestellt Frage ist, wie ein Grad der Behinderung beantragt wird
- Eine de Pflegeüberleitung häufig gestellt Frage ist, ob ein Anspruch geltend gemacht werden kann
- Eine de Pflegeüberleitung häufig gestellt Frage ist, wie eine vollmacht erteilt werden kann
- Allgemein wird fast immer beraten zu Möglichkeiten der Versorgung zu Hause, Leistungen der Kranken- und Pflegeversicherung, Pflegegrad, Hilfsmittelversorgung, Hausnotrufsysteme und Hilfe zur Pflege
- Wenn TN Patienten auffordert, einen Pflegeantrag auszufüllen, tun das nur zwei von zehn. Die anderen acht möchten, dass die TN es macht
- Mache Patienten/Angehörige wissen nicht, dass sie einen Pflegegrad selber beantragen können. Sie hinterlegen das Dokument beim Stationsarzt und warten darauf, dass dieser auf sie zukommt
- Beratung zu Versorgungsleistungen muss auf jeweilige Person und Situation abgestimmt sein. Faktoren wie Art der Versicherung, gewünschtes Pflegesetting und vorhandene Ressourcen bestimmen den Prozess maßgeblich
- Mache Patienten/Angehörige wissen nicht, dass sie einen Pflegegrad selber beantragen können. Sie hinterlegen das Dokument beim Stationsarzt und warten darauf, dass dieser auf sie zukommt
- Patienten, die zu geplanten OPs kommen, stellen erst nach dem Eingriff fest, dass sie Hilfe bei der Haushaltsführung benötigen. Ein Antrag dafür kann auch vor der OP aufgesetzt und bei der Krankenkasse eingereicht werden. Dann ist die Nachsorge auch zum Entlassungszeitpunkt abgesichert
- Menschen beschäftigen sich mit Pflegebedürftigkeit und Vorsorge nicht, solange sie nicht unmittelbar davon betroffen sind
- Infolge eines Informationsdefizits beziehen die Personen u.a. Leistungen von Sanitätshäusern, bei denen die Kosten nicht von der Krankenkasse übernommen werden
- SD berät und empfiehlt Patienten zu der Versorgungsform, die er bedarf und die auch faktisch umsetzbar ist
- Ärzte versprechen Pat. Dinge, die gesetzlich nicht bestand haben
- Ärzte führen eine vermeintliche Beratung der Patienten durch bzw. stellen diesen Versorgungsmaßnahmen in Aussicht, die so nicht umsetzbar sind
- Im Kern entsteht ein Problem daraus, dass Patienten vor Eingriffen nicht umfangreich aufgeklärt/beraten werden
- Resultat des oben beschriebenen Vorgehens ist, dass Pat. Sich einer geplanten OP unterziehen und danach verwundert sind, dass nicht sofort eine Nachsorge gewährleistet ist
- Häufig können Patienten ihre eigenen Bedarfe nicht nennen. SD nimmt dann Kontakt zu Angehörigen auf, um mit diesen die Bedarfe zu besprechen. Dies geschieht tlw. Gemeinsam mit Pat.

## Kategorie Herausforderungen und Verbesserungspotentiale

### Unterkategorie Ressourcenbedarf

#### Aussagen nach Workshops

**Workshop 1**

- SD soll gemäß Prozessdefinition und möchte ausführliche Beratung und psychosoziale Intervention durchführen. Kann dies aber nicht umsetzen, da Ressourcen für fachfremde Tätigkeiten gebraucht werden.

**Workshop 2**

- Wunsch nach mobilen Endgeräten zur Dokumentation
- Es gibt nur einen Bildschirm am Arbeitsplatz, das behindert die Tätigkeit, da es viele Daten zum Kopieen und Einfügen gibt.. Eine TN hat sich einen Bildschirm von zuhause mitgebracht.

**Workshop 3**

- Technische Ausstattung ist mangelhaft, Headsets werden nicht genehmigt.
- Headset wird nicht genehmigt. Dieses wird aber benötigt, da TN nicht telefonieren und dokumentieren gleichzeitig kann

**Workshop 5**

- MA hoffen, dass mobile Endgeräte zur Dokumentation am Patientenbett bald kommen

#### Gesamtdarstellung aller Punkte

- SD soll gemäß Prozessdefinition und möchte ausführliche Beratung und psychosoziale Intervention durchführen. Kann dies aber nicht umsetzen, da Ressourcen für fachfremde Tätigkeiten gebraucht werden.
- Wunsch nach mobilen Endgeräten zur Dokumentation
- Es gibt nur einen Bildschirm am Arbeitsplatz, das behindert die Tätigkeit, da es viele Daten zum Kopieen und Einfügen gibt.. Eine TN hat sich einen Bildschirm von zuhause mitgebracht.
- Technische Ausstattung ist mangelhaft, Headset wird nicht genehmigt. Dieses wird aber benötigt, da TN nicht telefonieren und dokumentieren gleichzeitig kann
- MA hoffen, dass mobile Endgeräte zur Dokumentation am Patientenbett bald kommen

### Unterkategorie Strukturelle Herausforderungen

#### Aussagen nach Workshops

**Workshop 1**

- Standard wird nicht eingehalten
- Viele KK als beteiligte Akteure

**Workshop 2**

- Sinnvoll wäre aus ihrer Sicht, dass Akteurübergreifend digital gearbtet wird
- Anamnesebögen schrecken ab, weil sie so groß sind
- Wunsch nach mobilen Endgeräten zur Dokumentation
- Immer wieder gestellte Fragen hemmen Arbeitsfortschritt des SD
- Komplexität und Anspruch an die Versorgung der Pat. Steigt seit Jahren. Diese werden im durchschnitt immer älter und morbider. Angehörige als Ressource (für Pat. + SD) entfällt zunehmend. Daher höhere Arbeitsaufwand pro Patient
- Teilnehmende Sieht grundlegenden Klärungsbedarf innerhalb der Organisation
- Alle Berufsgruppen haben Zeitprobleme und Personalmangel
- Es müssen dutzende Pflegeheime kontaktiert werden, bis ein Pflegeplatz zu finden ist. Der Prozess erfordert auch viel Wartezeit
- Bei Pflegeüberleitung müssen Verträge vor Entlassung geschlossen sein, da die Finanzierung nicht gewährleistet ist und der Nachversorger die Übernahme des Patienten nicht antritt
- Keine Aufnahme zur KZP ohne Vertrag
- Alle haben Zeitprobleme und Personalmangel
- Prozesse im Haus wurden angepasst, weil Assessments nicht zuverlässig durchgeführt werden und Kommunikation zum SD durch med. P. nicht autonom vorgenommen wird. Folge ist, dass SD nun regelmäßigen Termin mit Ärzten hat, um alle Patienten hinsichtlich eines Nachsorgebedarfes zu besprechen.

**Workshop 3**

- Angehörige sind oft in emotionalen Ausnahmesituationen. Dadurch sinkt ihre Konzentrations- und Merkfähigkeit
- Es fehlt einfach an Manpower
- 
- Obwohl Teile der Dokumentation schon vor Jahren digital angelegt wurden, wird immer noch vorrangig mit Papier gearbeitet und auf analoge Patientenakte verwiesen
- Mobiltelefon funktioniert auf der Station nicht, weil der Sendebereich unzureichend ist
- Im Patientenorganizer kann man einsehen, ob ein Patient bereits angemeldet ist

**Workshop 5**

- Hohe Auslastung des Gesundheitssystems und dadurch knappes Angebot als strukturelle Herausforderung für EM
- Plätze zur Nachversorgung im Pflegeheim sind schwer zu beschaffen

**Workshop 6:**

- Bei manchen Patienten kann die Bedarfserhebung mehrere Termine benötigen

- Pflegeheim-Ampel zur Symoblisierung der Auslastung von Einrichtungen gibt es schon, hilft aber nicht, da es allgemein kaum Kapazitäten gibt

### Gesamtdarstellung aller Punkte

- Standard wird nicht eingehalten
- Viele KK als beteiligte Akteure
- Akteure des Gesundheitswesens arbeiten mit unterschiedlichen Dokumentenmanagementsystemen und sind unterschiedlich digitalisiert
- Anamnesebögen schrecken ab, weil sie so groß sind
- Geringe Gesundheitskompetenz bzw. geringer Wissensstand seitens der Versicherten und deren Angehörigen erfordern sehr viel Beratung
- Komplexität und Anspruch an die Versorgung der Pat. Steigt seit Jahren. Diese werden im durchschnitt immer älter und morbider. Angehörige als Ressource (für Pat. + SD) entfällt zunehmend. Daher höhere Arbeitsaufwand pro Patient
- Alle Berufsgruppen haben Zeitprobleme und Personalmangel (Fachkräftemangel)
- Geringe Verfügbarkeit von Plätzen in der stationären Pflege
- Bei Pflegeüberleitung müssen Verträge vor Entlassung geschlossen sein, da die Finanzierung nicht gewährleistet ist und der Nachversorger die Übernahme des Patienten nicht antritt
- Angehörige sind oft in emotionalen Ausnahmesituationen. Dadurch sinkt ihre Konzentrations- und Merkfähigkeit
- Nicht ausreichend Personal für geforderte Tätigkeiten vorhanden
- Niedriger Grad der Digitalisierung: Obwohl Teile der Dokumentation schon vor Jahren digital angelegt wurden, wird immer noch vorrangig mit Papier gearbeitet und auf analoge Patientenakte verwiesen
- Mobiltelefon funktioniert auf der Station nicht, weil der Sendebereich unzureichend ist
- Hohe Auslastung des Gesundheitssystems und dadurch knappes Angebot als strukturelle Herausforderung für EM
- Plätze zur Nachversorgung im Pflegeheim sind schwer zu beschaffen
- Bei manchen Patienten kann die Bedarfserhebung mehrere Termine benötigen
- Pflegeheim-Ampel zur Symoblisierung der Auslastung von Einrichtungen gibt es schon, hilft aber nicht, da es allgemein kaum Kapazitäten gibt

## Unterkategorie Verbesserungspotentiale

### Aussagen nach Workshops

#### Workshop 1

- Wunsch nach zentraler Dokumentation von Kundenkontakt/-kommunikation
- Falsche Planung führt zu Mehrarbeit
- Anmeldungen zum EM bleiben oft aus oder erfolgen oft zu spät
- Wunsch nach transparenten Zuständigkeiten

**Workshop 2**

- Wenn SD Einsicht in Informationen der Derma hätte, würde er sich viele Telefonate sparen
- Anamnesebögen schrecken ab, weil sie so groß sind
- Mehrfachanmeldungen für den KLAU sind nervig. Sie entstehen, weil SD Befunde des Arztes braucht, um Anmeldung bei Nachversorger zu realisieren. Ärzte lesen aber die entsprechende Dokumentation des Pat. Nicht. Er oder die Pflege denken, dass der SD den EM nicht nachkommt und eröffnen einen neuen Auftrag. Mehrfachanmeldung kommen aber auch infolge von Verlegung des Patienten innerhalb der Einrichtung vor.
- Vorschlag zu Herausforderungen mit KLAU ist, dass ein Auftrag erst freigegeben wird, wenn alle erforderlichen Daten vorliegen und eine objektiv nachvollziehbare Notwendigkeit des EM gegeben ist
- Dokumentation im System ist teilweise sehr undurchsichtig. Einträge des SD werden teilweise durch MA unter älteren Einträgen fortgeführt. Andere legen neue Dokumente für aktuelles EM an. Besonders bei Patienten, die mehrere Aufenthalte habe, kann das zu Verwirrung führen
- Herausforderung liegt im vorhandenen SAP. TN sind mit den Funktionen des bestehenden Systems nicht zufrieden
- Rezepte werden oft falsch ausgestellt. Ärzte wissen oft nicht, dass nur ein Hilfsmittel pro Rezept von Sanitätshäusern akzeptiert wird. Eine KI könnte hier auf diesen Fehler im Erstellungsprozess hinweisen

**Workshop 3**

- Bei OP-Verschiebung erfolgt oft keine Benachrichtigung des EM. Nachsorge kann dann nicht angepasst werden.
- Wunsch, dass durch KI Zuständigkeit im EM für andere Berufsgruppen und Patienten/Angehörige transparenter ist. Dadurch können Fehler vermieden und Zeit gespart werden
- Sinnvoll wäre, wenn Angehörige oder Patienten durch Chatbot eine Liste mit Pflegediensten, Pflegestützpunkte in der häuslichen Umgebung erhalten

**Workshop 5**

- Sinnvoll könnte sein, dass KI den OP-Plan beobachtet und lernt, welche Eingriffe sehr Nachsorgeintensiv sind. Außerdem könnte dadurch die Nachversorgung besser abgestimmt werden.
- MA hoffen, dass mobile Endgeräte zur Dokumentation am Patientenbett bald kommen

**Workshop 6:**

- SD nehmen ausgedruckte Fälle, weil sie beim Patienten vor Ort dokumentieren müssen und die bestehenden Informationen vor Ort brauchen. Tablets gibt es nicht
- Vor dem Gespräch ist noch nicht bekannt, ob eine Anschlussbehandlung/Nachsorge benötigt oder gewünscht ist. Hilfreich wäre, wenn SD beim Patientenkontakt vor Ort spontan Zugriff auf Anträge hätte.

### Gesamtdarstellung aller Punkte

- zentrale Dokumentation von Kundenkontakt/-kommunikation
- Wenn SD Einsicht in Informationen der Derma hätte, würde er sich viele Telefonate sparen
- Anamnesebögen schrecken ab, weil sie so groß sind
- Mehrfachanmeldungen für den KLAU sind nervig. Sie entstehen, weil SD Befunde des Arztes braucht, um Anmeldung bei Nachversorger zu realisieren. Ärzte lesen aber die entsprechende Dokumentation des Pat. Nicht. Er oder die Pflege denken, dass der SD den EM nicht nachkommt und eröffnen einen neuen Auftrag.
- Mehrfachanmeldung zum EM infolge von Verlegung des Patienten
- Vorschlag zu Herausforderungen mit KLAU ist, dass ein Auftrag erst freigegeben wird, wenn alle erforderlichen Daten vorliegen und eine objektiv nachvollziehbare Notwendigkeit des EM gegeben ist
- Dokumentation im System ist teilweise sehr undurchsichtig. Einträge des SD werden teilweise durch MA unter älteren Einträgen fortgeführt. Andere legen neue Dokumente für aktuelles EM an. Besonders bei Patienten, die mehrere Aufenthalte habe, kann das zu Verwirrung führen
- Herausforderung liegt im vorhandenen SAP. TN sind mit den Funktionen des bestehenden Systems nicht zufrieden
- Rezepte werden oft falsch ausgestellt. Ärzte wissen oft nicht, dass nur ein Hilfsmittel pro Rezept von Sanitätshäusern akzeptiert wird. Eine KI könnte hier auf diesen Fehler im Erstellungsprozess hinweisen
- Bei OP-Verschiebung erfolgt oft keine Benachrichtigung des EM. Nachsorge kann dann nicht angepasst werden.
- Wunsch, dass durch KI Zuständigkeit im EM für andere Berufsgruppen und Patienten/Angehörige transparenter ist. Dadurch können Fehler vermieden und Zeit gespart werden
- Sinnvoll wäre, wenn Angehörige oder Patienten durch Chatbot eine Liste mit Pflegediensten, Pflegestützpunkte in der häuslichen Umgebung erhalten
- Sinnvoll könnte sein, dass KI den OP-Plan beobachtet und lernt, welche Eingriffe sehr Nachsorgeintensiv sind. Außerdem könnte dadurch die Nachversorgung besser abgestimmt werden.
- MA hoffen, dass mobile Endgeräte zur Dokumentation am Patientenbett bald kommen
- SD nehmen ausgedruckte Fälle, weil sie beim Patienten vor Ort dokumentieren müssen und die bestehenden Informationen vor Ort brauchen. Tablets gibt es nicht
- Vor dem Gespräch ist noch nicht bekannt, ob eine Anschlussbehandlung/Nachsorge benötigt oder gewünscht ist. Hilfreich wäre, wenn SD beim Patientenkontakt vor Ort spontan Zugriff auf Anträge hätte.

## Unterkategorie Herausforderungen und Barrieren

### Aussage nach Workshops

#### Workshop 2

- Immer wieder gestellte Fragen hemmen Arbeitsfortschritt des SD
- Herausfordernd sind Patienten ohne klaren Versicherungsschutz, Obdachlose ohne festen Wohnsitz, Geflüchtete und Ausländer. Hier besteht oft Unklarheit bzgl. Kostenübernahme

**Workshop 3**

- Pflege gibt Angehörigen die Telefonnummer des Sozialdienstes, weil sie dessen Zuständigkeit vermutet. Die ist aber im Bereich der KZP nicht gegeben, also muss TN den Angehörigen erklären, dass sie die falsche Ansprechpartnerin für ihn ist. Das ist zeitintensiv für MA und Angehörigen
- Folge des nicht korrekt angelegten KLAUs ist, dass weniger Zeit für die Organisation der Nachversorgung besteht und es zur Versorgungslücke kommen kann
- Informationen zu Kontaktdaten warden nicht eingetragen. Im Diagnosefeld wird auf den Arztbrief verwiesen. Das führt dazu, dass MA sehr viel recherchieren müssen
- Durch spärliche Dokumentation und Verweis auf Stationsakte müssen MA viel zu den entsprechenden Lagerorten laufen. Diese Wegzeit nimmt ihnen Ressourcen für andere Tätigkeiten
- Es kommt oft vor, dass KLAU bereits begonnen wird, aber nicht final bestätigt wird. In der Folge erreicht der die MA des SD nicht. Außerdem werden durch das Ausbleiben des EM weitere KLAUs angelegt
- Private Krankenkassen geben grundsätzliche keine Informationen an SD
- Station meldet sämtliche verfügbaren Optionen zur Nachsorge bei PÜ an. Diese weiß dann anhand der Anmeldung nicht, welcher Bedarf tatsächlich besteh
- Headset wird nicht genehmigt. Dieses wird aber benötigt, da TN nicht telefonieren und dokumentieren gleichzeitig kann

**Workshop 4**

- Faxe und Telefonate sind aufwändig
- TN erfährt meist nur durch aktive Recherche in der Dokumentation von für sie relevanten Änderungen beim Patienten
- Bei Fragen zu Patienten ist klinisches Personal für TN schwer erreichbar
- TN müssen Kontaktdaten suchen, weil sie nicht im System hinterlegt sind
- Es kommt vor, dass sich erst zum Ende des EM-Prozesses herausstellt, dass der/Die Patient:in einen Betreuer hat.
- Manchmal wird erst kurz vor der Entlassung festgestellt, dass ein Patient nicht geschäftstüchtig ist.
- Bei manchen Patieten sind Kontakdaten hinterlegt, die nicht mehr aktuell sind
- Bestehende Rahmenverträge zwischen Krankenkassen und Leistungserbringern wie Transportdiensten sind nur begrenzt gültig. Diese Daten werden nicht automatisch aktualisiert
- Patienten, die zu geplanten OPs kommen, stellen erst nach dem Eingriff fest, dass sie Hilfe bei der Haushaltsführung benötigen. Ein Antrag dafür kann auch vor der OP aufgesetzt und bei der Krankenkasse eingereicht werden. Dann ist die Nachsorge auch zum Entlassungszeitpunkt abgesichert

- Menschen beschäftigen sich mit Pflegebedürftigkeit und Vorsorge nicht, solange sie nicht unmittelbar davon betroffen sind
- Die Zuständigkeit für das jeweilige Anliegen der Versicherten ist für diese nicht immer nachvollziehbar. Sie wenden sich oft an den Leistungserbringer, mit dem sie zuletzt in Kontakt waren
- SD kann nicht alle Angelegenheiten selbst übernehmen. Gerade bei privat Versicherten haben sie keinen Einblick in die Vertragsbestandteile
- Besondere Konstellationen der Versicherungen erschweren die Versorgung (Bsp. Krankenversicherung gesetzlich, Pflegeversicherung privat)
- Aktuell werden analoge Daten in Papierakte hinterlegt und sind beim nächsten Aufenthalt des Patienten nicht mehr zielsicher auffindbar

**Workshop 5**

- Patienten werden oft entlassen, weil deren Pflegebett benötigt wird. Dies geschieht, ohne das mit zuständiger SD-MA abzusprechen. Diese kann ihre Beratung nicht vollständig durchführen.
- Oft kommt es dazu, dass die Anforderungen an den falschen Ansprechpartner gesandt werden. Das liegt daran, dass es mehrere Sozialarbeiter gibt. Manche gehören fest zu Organisationseinheiten.
- Unklarheit darüber, welche Berufsgruppe für den Bedarf des Pat. zuständig ist führt dazu, dass falscher Mitarbeitende eingeschaltet wird
- Innerhalb der Anforderung des EM wird der neue Aufenthaltsort des Pat. Nicht aktualisiert
- Manche Pat. Sprechen kein Deutsch, dann muss ein Dolmetscher gesucht werden
- Problem ist, dass Nachversorger nicht die gleiche Software nutzen. Über einzelne Kontaktanfragen per Mail oder Telefon geben sie zuverlässig Auskunft
- Bei entlassenen Patienten werden offene Einträge oft nicht vervollständigt. Ärzte schauen nicht mehr in die digitale Akte und der Eintrag des Befundes bleibt aus. KI sollte daran erinnern
- Auftrag ist bei SD noch offen, erscheint für den Arzt aber nicht
- Großes Problem ist Erreichbarkeit von Ärzten
- SD hat keine festen zuständigen Ansprechpartner im Bereich der Ärzte. Verantwortlichkeiten wechseln mitunter sehr schnell. Ärzte verweisen auf andere Zuständigkeit, Prozess des EM wird dadurch insgesamt negativ beeinflusst
- Zahl der Anrufe sehr hoch, erfordert viel Personalressourcen zur Koordinierung
- Privatkassen und Beihilfen sind zu 99% nicht zu erreichen. Sprechen auch nicht mit SD, nur mit Patienten. Kostenübernahmebescheide senden sie ausschließlich an Anschrift des Patient
- Patienten/Angeh. Sind unwissend über den Prozess des EM und die Arbeitsweise. Folge sind hohe Anzahl an Kontaktversuchen zu SD. Das wiederum belastet diesen.
- Klinikinterne Prozesse sind nicht aufeinander abgestimmt. Prozess der OP bedarf eigentlich der Abstimmung mit Prozess des EM, wird aber nicht vorgenommen. So kommt es Herausforderungen bei der Nachsorge und Versorgungsbruch.

- Möglichkeit der Markierung der hohen Dringlichkeit wird häufiger genutzt, als es  nach Ansicht des SD erforderlich wäre

**Workshop 6:**

- Wenn die Patientendaten falsch oder unvollständig durch die Patientenaufnahme erhoben wurden, hat TN Probleme beim Prozess
- Herausfordernd sind falsch eingetragene Telefonnummern oder Kontaktpersonen, die nicht mehr aktuell sind.
- Kommunikation mit den Ärzten wird als unzureichend empfunden. Absprachen werden mitunter nicht eingehalten
- Im Bereich der Reha-Vermittlung kommt es oft vor, dass Formulare vom EM an Ärzte zur Bearbeitung gesendet werden, die dann aber nur teilweise bis gar nicht ausgefüllt oder falsch bearbeitet sind. Oft muss auch mehrmals auf die Bearbeitung hingewiesen werden, bis sie vorgenommen wird. Angesprochene Ärzte sind oft nicht in der Zuständigkeit
- TN erhält nicht automatisch Rückmeldung, wenn sich entlassrelevante Aspekte beim Patienten ändern
- Wenn ein Übergang eines Patienten vom KH ist die Reha aus Sicht der Ärzte zu lange dauert, wird manchmal eine weitere Anmeldung zur KZP vorgenommen.
- Verschiedene Ärzte melden selben Patienten mehrmals und für unterschiedliche Nachsorger an
- Ohne Überleitbogen kann kein Platz beim Pflegedienst oder im Pflegeheim zustande kommen. Dieser muss von der Pflege erstellt werden, das dauert aber sehr lange. Gründe dafür sind, dass in der Akte nicht ausreichend dokumentiert wurde und angesprochene Pflegekräfte den Patienten oft noch nicht kennen
- Herausforderungen im Zusammenhang mit Kostenträgern sind, dass diese schwer erreichbar sind und Anträge, die per Fax gesandt werden, lange Bearbeitungszeiten haben oder verloren gehen
- Herausfordernd ist Beschaffung von Hilfsmittel bei Sanitätshäusern. Da nicht jedes Sanitätshaus einen Rahmenvertrag mit jeder KK hat, können nicht alle Hilfsmittel bei einem Sanitätshaus bezogen werden. Das führt dazu, dass ein teil der Hilfsmittel bei einem bestellt werden kann, der Rest aber von einem anderen geliefert werden muss und kostet den SD viel Zeit
- Teilweise ist sehr intransparent, welche Hilfsmittel bei den Sanitätshäusern für Angehöriger einer Krankenkasse übernommen werden
- Herausforderung bei Prozessbeginn ist, dass Inhalte der Anforderungen unklar, nicht vorhanden oder widersprüchlich sind.
- Problem ist auch, wenn Anmeldung zum EM zu spät erfolgt
- Herausforderung zu Prozessbeginn ist, wenn keine direkte Telefonnummer zum Arzt hinterlegt ist, der SD angefordert hat. Oft muss SD bei diesem noch Informationen einholen, weil sie nicht eindeutig sind
- Nachsorgebedarf wird bei Anforderung des EM nicht korrekt angegeben.
- Mangelnde Einarbeitung der Ärzte, insbesondere der Assistenzärzte ist ein Problem
- Bei dieser Gruppe von TN ist das Auffinden der Patienten weniger ein Problem. Schwierig ist es u.U. den Patienten zum richtigen Zeitpunkt anzutreffen, da SD keine Einsicht in seine Termine hat

- Herausforderung liegt darin, dass manche Termine spontan frei werden und Patient ohne Vorankündigung zur Untersuchung geht

## Gesamtdarstellung aller Punkte

- Immer wieder gestellte Fragen hemmen Arbeitsfortschritt des SD
- Herausfordernd sind Patienten ohne klaren Versicherungsschutz, Obdachlose ohne festen Wohnsitz, Geflüchtete und Ausländer. Hier besteht oft Unklarheit bzgl. Kostenübernahme
- Pflege gibt Angehörigen die Telefonnummer des Sozialdienstes, weil sie dessen Zuständigkeit vermutet. Die ist aber im Bereich der KZP nicht gegeben, also muss TN den Angehörigen erklären, dass sie die falsche Ansprechpartnerin für ihn ist. Das ist zeitintensiv für MA und Angehörigen
- Folge des nicht korrekt angelegten KLAUs ist, dass weniger Zeit für die Organisation der Nachversorgung besteht und es zur Versorgungslücke kommen kann
- Kontaktdaten von Angehörigen werden selten eingetragen. Im Diagnosefeld wird oft auf den Arztbrief als Informationsquelle verwiesen. Das führt dazu, dass MA sehr viel recherchieren müssen
- Durch spärliche Dokumentation und Verweis auf Stationsakte als Informationsquelle müssen MA viel zu den entsprechenden Lagerorten laufen. Diese Wegzeit nimmt ihnen Ressourcen für andere Tätigkeiten
- Es kommt oft vor, dass KLAU bereits begonnen wird, aber nicht final bestätigt wird. In der Folge erreicht der die MA des SD nicht. Außerdem werden durch das Ausbleiben des EM weitere KLAUs angelegt
- Private Krankenkassen geben grundsätzliche keine Informationen an SD
- Station meldet sämtliche verfügbaren Optionen zur Nachsorge bei PÜ an. Diese weiß dann anhand der Anmeldung nicht, welcher Bedarf tatsächlich besteh
- Headset wird nicht genehmigt. Dieses wird aber benötigt, da TN nicht telefonieren und dokumentieren gleichzeitig kann
- Faxe und Telefonate sind aufwändig
- TN erfährt meist nur durch aktive Recherche in der Dokumentation von für sie relevanten Änderungen beim Patienten
- Bei Fragen zu Patienten ist klinisches Personal für TN schwer erreichbar
- TN müssen Kontaktdaten suchen, weil sie nicht im System hinterlegt sind
- Es kommt vor, dass sich erst zum Ende des EM-Prozesses herausstellt, dass der/Die Patient:in einen Betreuer hat.
- Manchmal wird erst kurz vor der Entlassung festgestellt, dass ein Patient nicht geschäftstüchtig ist.
- Bei manchen Patieten sind Kontakdaten hinterlegt, die nicht mehr aktuell sind
- Bestehende Rahmenverträge zwischen Krankenkassen und Leistungserbringern wie Transportdiensten sind nur begrenzt gültig. Diese Daten werden nicht automatisch aktualisiert
- Patienten, die zu geplanten OPs kommen, stellen erst nach dem Eingriff fest, dass sie Hilfe bei der Haushaltsführung benötigen. Ein Antrag dafür kann auch

vor der OP aufgesetzt und bei der Krankenkasse eingereicht werden. Dann ist die Nachsorge auch zum Entlassungszeitpunkt abgesichert

- Menschen beschäftigen sich mit Pflegebedürftigkeit und Vorsorge nicht, solange sie nicht unmittelbar davon betroffen sind
- Die Zuständigkeit für das jeweilige Anliegen der Versicherten ist für diese nicht immer nachvollziehbar. Sie wenden sich oft an den Leistungserbringer, mit dem sie zuletzt in Kontakt waren
- SD kann nicht alle Angelegenheiten für die Versicherten übernehmen. Gerade bei privat Versicherten haben sie keinen Einblick in die Vertragsbestandteile
- Besondere Konstellationen der Versicherungen erschweren die Versorgung (Bsp. Krankenversicherung gesetzlich, Pflegeversicherung privat)
- Aktuell werden analoge Daten in Papierakte hinterlegt und sind beim nächsten Aufenthalt des Patienten nicht mehr zielsicher auffindbar
- Patienten werden oft entlassen, weil deren Pflegebett benötigt wird. Dies geschieht, ohne das mit zuständiger SD-MA abzusprechen. Diese kann ihre Beratung nicht vollständig durchführen.
- Oft kommt es dazu, dass die Anforderungen an den falschen Ansprechpartner gesandt werden. Das liegt daran, dass es mehrere Sozialarbeiter gibt. Manche gehören fest zu Organisationseinheiten.
- Unklarheit darüber, welche Berufsgruppe für den Bedarf des Pat. zuständig ist führt dazu, dass falscher Mitarbeitende eingeschaltet wird
- Innerhalb der Anforderung des EM wird der neue Aufenthaltsort des Pat. Nicht aktualisiert
- Manche Pat. Sprechen kein Deutsch, dann muss ein Dolmetscher gesucht werden
- Problem ist, dass Nachversorger nicht die gleiche Software nutzen. Über einzelne Kontaktanfragen per Mail oder Telefon geben sie zuverlässig Auskunft
- Bei entlassenen Patienten werden offene Einträge oft nicht vervollständigt. Ärzte schauen nicht mehr in die digitale Akte und der Eintrag des Befundes bleibt aus. KI sollte daran erinnern
- Auftrag ist bei SD noch offen, erscheint für den Arzt aber nicht
- Großes Problem ist Erreichbarkeit von Ärzten, die Kommunikation mit ihnen wird allgemein als unzureichend empfunden. Absprachen werden mitunter nicht eingehalten
- SD hat keine festen zuständigen Ansprechpartner im Bereich der Ärzte. Verantwortlichkeiten wechseln mitunter sehr schnell. Ärzte verweisen auf andere Zuständigkeit, Prozess des EM wird dadurch insgesamt negativ beeinflusst
- Zahl der Anrufe sehr hoch, erfordert viel Personalressourcen zur Koordinierung
- Privatkassen und Beihilfen sind zu 99% nicht zu erreichen. Sprechen auch nicht mit SD, nur mit Patienten. Kostenübernahmebescheide senden sie ausschließlich an Anschrift des Patient
- Patienten/Angeh. Sind unwissend über den Prozess des EM und die Arbeitsweise. Folge sind hohe Anzahl an Kontaktversuchen zu SD. Das wiederum belastet diesen.
- Klinikinterne Prozesse sind nicht aufeinander abgestimmt. Prozess der OP bedarf eigentlich der Abstimmung mit Prozess des EM, wird aber nicht

vorgenommen. So kommt es Herausforderungen bei der Nachsorge und Versorgungsbruch.

- Möglichkeit der Markierung der hohen Dringlichkeit wird häufiger genutzt, als es  nach Ansicht des SD erforderlich wäre
- Wenn die Patientendaten falsch oder unvollständig durch die Patientenaufnahme erhoben wurden, hat TN Probleme beim Prozess
- Herausfordernd sind falsch eingetragene Telefonnummern oder Kontaktpersonen, die nicht mehr aktuell sind.
- Im Bereich der Reha-Vermittlung kommt es oft vor, dass Formulare vom EM an Ärzte zur Bearbeitung gesendet werden, die dann aber nur teilweise bis gar nicht ausgefüllt oder falsch bearbeitet sind. Oft muss auch mehrmals auf die Bearbeitung hingewiesen werden, bis sie vorgenommen wird. Angesprochene Ärzte sind oft nicht in der Zuständigkeit
- TN erhält nicht automatisch Rückmeldung, wenn sich entlassrelevante Aspekte beim Patienten ändern
- Wenn ein Übergang eines Patienten vom KH ist die Reha aus Sicht der Ärzte zu lange dauert, wird manchmal eine weitere Anmeldung zur KZP vorgenommen.
- Verschiedene Ärzte melden selben Patienten mehrmals und für unterschiedliche Nachsorger an
- Ohne Überleitbogen kann kein Platz beim Pflegedienst oder im Pflegeheim zustande kommen. Dieser muss von der Pflege erstellt werden, das dauert aber sehr lange. Gründe dafür sind, dass in der Akte nicht ausreichend dokumentiert wurde und angesprochene Pflegekräfte den Patienten oft noch nicht kennen
- Herausforderungen im Zusammenhang mit Kostenträgern sind, dass diese schwer erreichbar sind und Anträge, die per Fax gesandt werden, lange Bearbeitungszeiten haben oder verloren gehen
- Herausfordernd ist Beschaffung von Hilfsmittel bei Sanitätshäusern. Da nicht jedes Sanitätshaus einen Rahmenvertrag mit jeder KK hat, können nicht alle Hilfsmittel bei einem Sanitätshaus bezogen werden. Das führt dazu, dass ein teil der Hilfsmittel bei einem bestellt werden kann, der Rest aber von einem anderen geliefert werden muss und kostet den SD viel Zeit. Außerdem ist teilweise sehr intransparent, welche Hilfsmittel bei den Sanitätshäusern für Angehöriger einer Krankenkasse übernommen werden
- Herausforderung bei Prozessbeginn ist, dass Inhalte der Anforderungen des Entlassmanagements durch das medizinische Personal unklar, nicht vorhanden oder widersprüchlich sind. Nachsorgebedarf wird bei Anforderung des EM nicht korrekt angegeben.
- Problem ist auch, wenn Anmeldung zum EM zu spät erfolgt
- Herausforderung zu Prozessbeginn ist, wenn keine direkte Telefonnummer zum Arzt hinterlegt ist, der SD angefordert hat. Oft muss SD bei diesem noch Informationen einholen, weil sie nicht eindeutig sind
- Mangelnde Einarbeitung der Ärzte, insbesondere der Assistenzärzte ist ein Problem
- Bei dieser Gruppe von TN ist das Auffinden der Patienten weniger ein Problem. Schwierig ist es u.U. den Patienten zum richtigen Zeitpunkt anzutreffen, da SD keine Einsicht in seine Termine hat. Herausforderung liegt darin, dass manche Termine spontan frei werden und Patient ohne Vorankündigung zur Untersuchung geht
