# Supplementary figures and images for: Optimizing Hospital Discharge Planning: Empirical Insights and Requirements of AI-Based Technologies From an Explorative Mixed Methods Field Study
Source: JMIR Form Res. 2026 Mar 24;10:e81824. doi: 10.2196/81824 (PMC13012232; doi:10.2196/81824)

Patient Discharge Process Flowchart

Hospital\_Staff

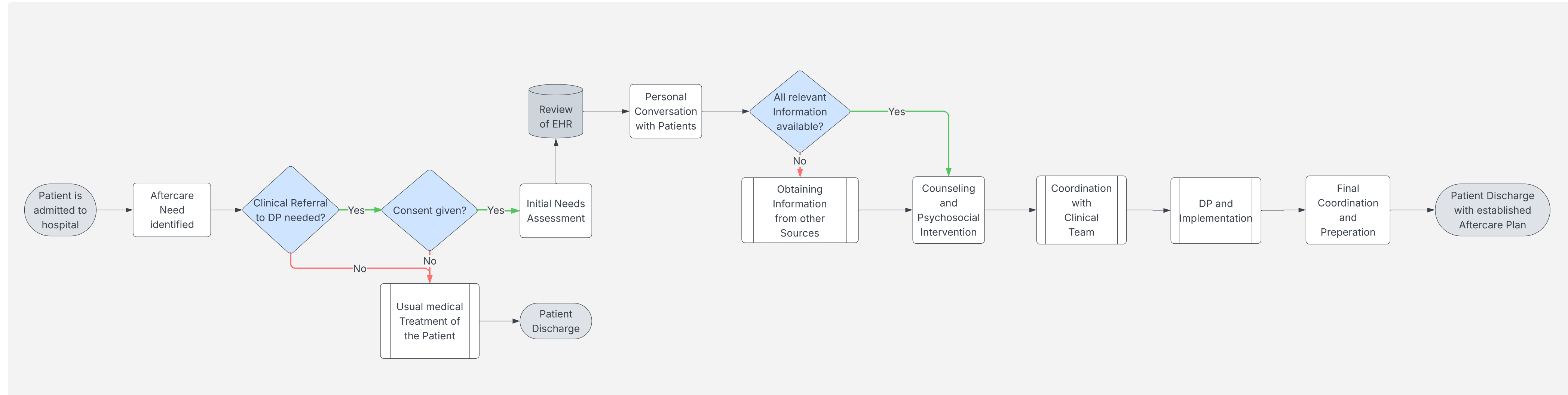

Supplement: Multimedia Appendix 9 [file formative-v10-e81824-s009.pdf]
