# Supplementary material for: Optimizing Hospital Discharge Planning: Empirical Insights and Requirements of AI-Based Technologies From an Explorative Mixed Methods Field Study
Source: JMIR Form Res. 2026 Mar 24;10:e81824. doi: 10.2196/81824 (PMC13012232; doi:10.2196/81824)
Supplement: Checklist 1 [file formative-v10-e81824-s010.pdf]

**Good Reporting of a Mixed Methods Study (GRAMMS) Guideline**

| **Good Reporting of a Mixed Methods Study (GRAMMS) Guideline**<br><br>(O'Cathain et al., 2008) | **Section: Page** |
|---|---|
| Describe the justification for using a mixed methods approach to the research question | Background and related work: p.4<br>Methodology: p.5 |
| Describe the design in terms of the purpose, priority and sequence of methods | Methodology: p.5; pp. 7-8 |
| Describe each method in terms of sampling, data collection and analysis | Methodology: pp.5-8 |
| Describe where integration has occurred, how it has occurred and who has participated in it<br><br>*Beschreiben Sie, wo Integration stattgefunden hat, wie sie stattgefunden hat und wer daran teilgenommen hat* | Data analysis: p.8 |
| Describe any limitation of one method associated with the present of the other method | Limitations: pp.20-21 |
| Describe any insights gained from mixing or integrating methods | Data analysis: p.8<br>Discussion: p.18; p.20 |

O'Cathain, A., Murphy, E., & Nicholl, J. (2008). The quality of mixed methods studies in health services research. *Journal of Health Services Research & Policy*, *13*(2), 92–98. https://doi.org/10.1258/jhsrp.2007.007074
